# Supplementary material for: National survey of attitudes towards and intentions to vaccinate against COVID-19: implications for communications
Source: BMJ Open. 2021 Oct 27;11(10):e055085. doi: 10.1136/bmjopen-2021-055085 (PMC8557244; doi:10.1136/bmjopen-2021-055085)
Supplement: Supplementary data [file bmjopen-2021-055085supp001.pdf]

Supplementary material to 'A national survey of attitudes towards and intentions to vaccinate against COVID-19: implications for communications'.

Contents

- Methods S1: Questionnaire for the OPTIMising general public Uptake of a COVID-19 vaccine (OPTIMUM) study.
- Table S1 Overall response rate calculation accounting for recruitment onto original panel and panel attrition.
- Methods S2: Non-response weights.
- Table S2 Doctors, nurses or other healthcare professionals – Association between trust in sources of information about COVID-19 vaccine and socio-demographic variables – (a) bivariate results and (b) multivariate logistic regression.
- Table S3 Pharmacists – Association between trust in sources of information about COVID-19 vaccine and socio-demographic variables – (a) bivariate results and (b) multivariate logistic regression.
- Table S4 The NHS – Association between trust in sources of information about COVID-19 vaccine and socio-demographic variables – (a) bivariate results and (b) multivariate logistic regression.
- Table S5 Drug companies who manufacture vaccines – Association between trust in sources of information about COVID-19 vaccine and socio-demographic variables – (a) bivariate results and (b) multivariate logistic regression.
- Table S6 The UK Government – Association between trust in sources of information about COVID-19 vaccine and socio-demographic variables – (a) bivariate results and (b) multivariate logistic regression.
- Table S7 The Scottish/Welsh Government – Association between trust in sources of information about COVID-19 vaccine and socio-demographic variables – (a) bivariate results and (b) multivariate logistic regression.
- Table S8 Scientific and medical advisers – Association between trust in sources of information about COVID-19 vaccine and socio-demographic variables – (a) bivariate results and (b) multivariate logistic regression.
- Table S9 The World Health Organisation (WHO) – Association between trust in sources of information about COVID-19 vaccine and socio-demographic variables – (a) bivariate results and (b) multivariate logistic regression.
- Table S10 The media (e.g. newspapers, magazines, television, radio) – Association between trust in sources of information about COVID-19 vaccine and socio-demographic variables – (a) bivariate results and (b) multivariate logistic regression.
- Table S11 Social media – Association between trust in sources of information about COVID-19 vaccine and socio-demographic variables – (a) bivariate results and (b) multivariate logistic regression.
- Table S12 Celebrities and social media influencers – Association between trust in sources of information about COVID-19 vaccine and socio-demographic variables – (a) bivariate results and (b) multivariate logistic regression.
- Table S13 Family and friends – Association between trust in sources of information about COVID-19 vaccine and socio-demographic variables – (a) bivariate results and (b) multivariate logistic regression.
- Table S14 Faith or community leaders – Association between trust in sources of information about COVID-19 vaccine and socio-demographic variables – (a) bivariate results and (b) multivariate logistic regression.

Stead M, *et al. BMJ Open* 2022; 11:e055085. doi: 10.1136/bmjopen-2021-055085

Methods S1: Questionnaire for the OPTIMising general public Uptake of a COVID-19 vaccine (OPTIMUM) study

Socio-demographic questions
{ASK IF DemogUpd = 0}
EconAct
Which of these descriptions applied to what you spent the **most** time doing last week, that is the seven days ending last Sunday?

_WEB: "Please select one answer on every row"
_TEL: "INTERVIEWER: READ OUT EACH STATEMENT AND THE ANSWER CODES. REPEAT ANSWER CODES AS REQUIRED."

1. In full-time education (including on vacation)
2. On government training/employment programme
3. In paid work (or away temporarily, including furlough) for at least 10 hours in week
4. Waiting to take up paid work already accepted
5. Unemployed
6. Permanently sick or disabled
7. Wholly retired from work
8. Looking after your home or family
9. Doing something else

{ASK ALL}
C19HiRsk
Since the start of the COVID-19 outbreak, have you ever been contacted by your GP or Healthcare Provider to say that you are at severe risk from COVID-19 and advised to shield?

1. Yes
2. No
3. Don't know

{ASK ALL}
C19HiRskHH
And since the start of the COVID-19 outbreak, has anyone else in your household ever been contacted by their GP or Healthcare Provider to say that they are at severe risk from COVID-19 and advised to shield?

1. Yes
2. No
3. Don't know

{ASK IF FF_Sex = 2 AND FF_Age LT 50}
Preg
"Are you currently pregnant?"

1. Yes
2. No

{ASK IF Cur_EconAct = 3 OR EconAct = 3}
EmpCond [MULTICODE: RANDOMISE 1…3]
"Which, if any, of the following apply to you?

_WEB: "Please select all that apply"
_TEL: "INTERVIEWER: READ OUT EACH OPTION AND CODE ALL THAT APPLY"

1. In my current job I'm required to work in close proximity with other people
2. I work in social care and have direct contact with patients or members of the public
3. I work in health care and have direct contact with patients or members of the public
4. None of these [EXCLUSIVE]

Vaccines
{ASK ALL}
VaccQInt
“The next set of questions will ask you about your views on vaccines in general.”

DISPLAY

{ASK ALL}
VaccSafe [FLIP SCALE]
In general, how often do you think vaccines cause serious side effects?

_WEB: “Please select one answer on every row”
_TEL: “INTERVIEWER: READ OUT EACH STATEMENT AND THE ANSWER CODES. REPEAT ANSWER CODES AS REQUIRED.”

1. Always
2. Frequently
3. Sometimes
4. Rarely
5. Never

{ASK ALL}
VaccMildSE [FLIP SCALE]
In general, how likely would you be to accept a vaccine that caused mild side effects?

By mild side effects we mean things like a mild fever, pain or swelling at the injection site, or feeling a bit unwell for a few days

_WEB: “Please select one answer on every row”
_TEL: “INTERVIEWER: READ OUT EACH STATEMENT AND THE ANSWER CODES. REPEAT ANSWER CODES AS REQUIRED.”

1. Very likely
2. Quite likely
3. Neither likely nor unlikely
4. Quite unlikely
5. Very unlikely

{ASK ALL}
VaccEffec [FLIP SCALE]
How much protection do you think the flu vaccine provides against flu?

INTERVIEWER: READ OUT

1. Complete protection
2. A lot of protection
3. Some protection
4. A little protection
5. No protection at all

{ASK ALL}
VaccAtt [GRID; FLIP SCALE; RANDOMISE ROWS]
“{WEB: “Below are”; TEL: “I will now read out”} some statements about vaccines in general.

To what extent do you agree or disagree with each of the following?

_WEB: “Please select one answer on every row”
_TEL: “INTERVIEWER: READ OUT EACH STATEMENT AND THE ANSWER CODES. REPEAT ANSWER CODES AS REQUIRED.”

Stead M, *et al. BMJ Open* 2022; 11:e055085. doi: 10.1136/bmjopen-2021-055085

GRID ROWS
1. Government decisions about vaccines are made in people’s best interests
2. My immune system is strong enough that I don’t need most vaccines
3. The illnesses that vaccines prevent are not severe enough for me to get vaccinated
4. I get vaccinated because it helps to protect other people as well as me
5. I follow the recommendation of healthcare professionals when deciding whether or not to get a vaccine

GRID COLS
1. Strongly agree
2. Agree
3. Neither agree nor disagree
4. Disagree
5. Strongly disagree

{ASK ALL}
VaccAccepCh [FLIP SCALE 1…4]
Thinking about times a healthcare professional has recommended your children get a vaccine, how often have you followed that recommendation (that is, if you have any children)?

_WEB: “Please select one answer on every row”
_TEL: “INTERVIEWER: READ OUT EACH STATEMENT AND THE ANSWER CODES. REPEAT ANSWER CODES AS REQUIRED.”

1. All of the time
2. Most of the time
3. Some of the time
4. None of the time
5. They have never been offered a vaccine
6. I do not have any children

{ASK ALL}
VaccAccep [FLIP SCALE 1…4]
And thinking about times a health-care professional has recommended you get a vaccine, how often have you followed that recommendation?

_WEB: “Please select one answer on every row”
_TEL: “INTERVIEWER: READ OUT EACH STATEMENT AND THE ANSWER CODES. REPEAT ANSWER CODES AS REQUIRED.”

1. All of the time
2. Most of the time
3. Some of the time
4. None of the time
5. I have never been offered a vaccine

Covid-19 Vaccine
{ASK ALL}
C19VaccQInt
“The next set of questions will ask you about your views on a vaccine for COVID-19.”

DISPLAY

{ASK ALL}
C19VaccOff
“Have you been offered a vaccine for COVID-19?

1. Yes
2. No

{IF C19VaccOff = 1}

C19VaccAcc1
"And have you had that vaccine?"

{WEB: "Please select 'Yes' if you have only had one of multiple doses"}
INTERVIEWER: "Please include if you have only had one of multiple doses"

1. Yes
2. No

{IF C19VaccAcc1 = 2}
C19VaccInt
"And do you intend to have that vaccine?"

_WEB: "Please select one answer on every row"
_TEL: "INTERVIEWER: READ OUT EACH STATEMENT AND THE ANSWER CODES. REPEAT ANSWER CODES AS REQUIRED."

1. Yes
2. No
3. Not sure

{IF C19VaccOff <> 1}
C19VaccAcc2
Would you accept the vaccine for yourself if it is offered to you?

_WEB: "Please select one answer on every row"
_TEL: "INTERVIEWER: READ OUT EACH STATEMENT AND THE ANSWER CODES. REPEAT ANSWER CODES AS REQUIRED."

1. Yes
2. No
3. Not sure

{IF C19VaccAcc2 = 3 or -8}
C19VaccAcc3
"Thank you for your response.

We would really like to know your opinion on this, even if you are unsure or don't feel you know enough.

If you had to choose, if a COVID-19 vaccine became publicly available and you were offered it, would you accept the vaccine for yourself?

_WEB: "Please select one answer on every row"
_TEL: "INTERVIEWER: READ OUT EACH STATEMENT AND THE ANSWER CODES. REPEAT ANSWER CODES AS REQUIRED."

1. Yes
2. No
3. I'm really not sure

{IF C19VaccInt = 2,3, -8 OR C19VaccAcc2 = 2 OR C19VaccAcc3 = 2,3, -8}
C19VaccWhyNo [MULTICODE: RANDOMISE 1…12]
For which, if any, of the following reasons {IF C19VaccInt = 2: "did you not"; IF C19VaccAcc2 = 2 OR C19VaccAcc3 = 2: "would you not"; IF C19VaccAcc3 = 3 or -8 or C19VaccInt =3 or -8: "are you unsure if you would"} accept a vaccine for COVID-19?

_WEB: "Please select all that apply"
_TEL: "INTERVIEWER: READ OUT EACH OPTION AND CODE ALL THAT APPLY"

1. I don't think COVID-19 is severe enough

2. I am concerned that vaccines are being rushed in
3. I am concerned that the vaccines have not been properly tested
4. I am frightened of needles
5. I don’t feel that I have enough information about the vaccines
6. I don’t think that the vaccines would be effective
7. I am worried about ingredients in the vaccines
8. I am worried that I would have a bad reaction or be allergic to it
9. I don’t trust the motives of those involved in developing COVID-19 vaccines (governments, pharmaceutical companies etc.).
10. I do not believe in vaccines
11. I feel I don’t need a vaccine
12. It would be inconvenient for me to get vaccinated
13. Other reason (Please describe)
14. None of these (EXCLUSIVE]

{ASK ALL}
C19VaccDec [GRID; FLIP SCALE 1…5; RANDOMISE ROWS]
How much {IF C19VaccOff = 1: “did”; IF C19VaccOff <> 1: “would”} your decision to get a COVID-19 vaccine depend on each of the following?

_WEB: “Please select one answer on every row”
_TEL: “INTERVIEWER: READ OUT EACH STATEMENT AND THE ANSWER CODES. REPEAT ANSWER CODES AS REQUIRED.”

GRID ROWS
1. The country in which the vaccine is developed
2. Whether or not the vaccine is recommended by my GP/healthcare professional
3. Whether or not the vaccine is recommended by the NHS
4. Whether or not the vaccine has been tested in large trials
5. Whether or not the vaccine has been in use for a few months with no serious side-effects
6. Whether or not people I know had already had the vaccine
7. Whether or not my GP/healthcare professional had already had the vaccine
8. Whether or not my local faith leader had recommended it
9. How easy or difficult it is to get the vaccine
10. Whether or not more than one injection was needed to provide adequate protection
11. Whether or not it would allow me to get my life back (be able to go out socialising, get back to work etc)
12. Whether or not it would help to protect members of my family who are vulnerable to COVID-19

GRID COLS
1. Completely
2. A great deal
3. Somewhat
4. Very little
5. Not at all

{ASK ALL}
C19VaccAccFF [FLIP SCALE 1…5]
“Thinking about your family and friends, how many do you think would get vaccinated against COVID-19 if a vaccine was offered to them?

_WEB: “Please select one answer on every row”
_TEL: “INTERVIEWER: READ OUT EACH STATEMENT AND THE ANSWER CODES. REPEAT ANSWER CODES AS REQUIRED.”

1. All of them
2. Most of them
3. About half of them
4. Some of them
5. None of them
6. Not applicable

{ASK ALL}
C19VaccSupFF [FLIP SCALE 1…5]
"To what extent do you think your family and friends support or oppose you getting vaccinated against COVID-19?"

_WEB: "Please select one answer on every row"
_TEL: "INTERVIEWER: READ OUT EACH STATEMENT AND THE ANSWER CODES. REPEAT ANSWER CODES AS REQUIRED."

1. Strongly oppose
2. Oppose
3. Neither oppose nor support
4. Support
5. Strongly support
6. Not applicable

{ASK ALL}
C19VaccTrstInf [GRID; FLIP SCALE; RANDOMISE ROWS]
"Thinking about {IF C19VaccOff = 1: "when"; IF C19VaccOff <> 1: "if"} you had to make a decision on whether or not to get a COVID-19 vaccine…

To what extent, if at all, would you trust information about a COVID-19 vaccine from each of the following sources?

_WEB: "Please select one answer on every row"
_TEL: "INTERVIEWER: READ OUT EACH STATEMENT AND THE ANSWER CODES. REPEAT ANSWER CODES AS REQUIRED."

GRID ROWS
1. Doctors, nurses, or other healthcare professionals
2. Pharmacists
3. The NHS
4. Drug companies who manufacture vaccines
5. The UK Government
6. {IF Cur_Country = 2: "The Scottish government"; IF Cur_Country = 3: "The Welsh Assembly"}
7. Scientific and medical advisers
8. The World Health Organisation (WHO)
9. The media (e.g. newspapers, magazines, television, radio)
10. Social media (e.g. Twitter, Facebook, Instagram etc)
11. Celebrities and social media influencers
12. Family and friends
13. Faith or community leaders

GRID COLS
1. Completely
2. A great deal
3. Somewhat
4. Very little
5. Not at all

{ASK ALL}
C19VaccPriAccep [GRID; FLIP SCALE; RANDOMISE ROWS]
A COVID-19 vaccine will be offered to some groups of people before other groups, and it is possible that not everyone in the population will be offered a COVID-19 vaccine.

How acceptable or unacceptable do you think each of the following are?

_WEB: "Please select one answer on every row"
_TEL: "INTERVIEWER: READ OUT EACH STATEMENT AND THE ANSWER CODES. REPEAT ANSWER CODES AS REQUIRED."

GRID ROWS
1. Some people being offered a COVID-19 vaccine before others
2. Some people not being offered a COVID-19 vaccine at all

GRID COLS
1. Very acceptable
2. Somewhat acceptable
3. Neither acceptable nor unacceptable
4. Somewhat unacceptable
5. Very unacceptable

{ASK ALL}
C19VaccPri [GRID; FLIP SCALE 1…5; RANDOMISE ROWS]
"{WEB: "Below are"; TEL: "I will now read out"} some groups that some people say should be the first to be offered a COVID-19 vaccine. For each one, how high a priority do you think it is that they get a COVID-19 vaccine, or do you not think they should be offered the vaccine at all?

Please answer on a scale of 1 to 5 where 1 means you think they should be one of the first groups to be offered the vaccine, and 5 means you think they should be one of the last groups to be offered the vaccine.

_WEB: "Please select one answer on every row"
_TEL: "INTERVIEWER: READ OUT EACH STATEMENT AND THE ANSWER CODES. REPEAT ANSWER CODES AS REQUIRED."

GRID ROWS
1. Doctors, nurses, and other healthcare professionals
2. People aged 18 to 30
3. Social care workers
4. People aged under 18
5. People with serious health conditions which mean they are vulnerable to COVID-19
6. Residents in a care home
7. Care home workers
8. People aged 80 or over
9. People aged 31-50
10. People with jobs that involve direct contact with members of the public
11. Schoolteachers

GRID COLS
1. 1 – One of the first
2. 2
3. 3
4. 4
5. 5 – One of the last
6. They should not be offered a vaccine

{ASK ALL}
C19VaccDoseImp [FLIP SCALE 1…4]
"How important, if at all, do you think it is for people to get the second injection of the COVID-19 vaccine?"

_WEB: "Please select one answer on every row"
_TEL: "INTERVIEWER: READ OUT EACH STATEMENT AND THE ANSWER CODES. REPEAT ANSWER CODES AS REQUIRED."

1. Very important
2. Fairly important
3. Not very important
4. Not at all important

Covid-19 attitudes
{ASK ALL}

C19VaccAttQInt
"The next set of questions will ask you about your views and experiences of COVID-19.

Some people may find these questions sensitive. Remember, you do not have to answer any questions you would prefer not to."

DISPLAY

{ASK ALL}
C19InfoEas [GRID; FLIP SCALE 1…5; RANDOMISE ROWS]
"How easy or difficult do you find each of the following?

_WEB: "Please select one answer on every row"
_TEL: "INTERVIEWER: READ OUT EACH STATEMENT AND THE ANSWER CODES. REPEAT ANSWER CODES AS REQUIRED."

GRID ROWS
1. Finding information to help you make decisions about your health
2. Finding information about how to protect yourself and others from COVID-19
3. Finding information on what to do if you have symptoms of COVID-19
4. Understanding the current instructions and guidance on how to protect yourself and others from COVID-19

GRID COLS
1. Very easy
2. Fairly easy
3. Neither easy nor difficult
4. Fairly difficult
5. Very difficult
6. Not applicable

{ASK ALL}
C19Diag
"Have you been officially diagnosed with the coronavirus (COVID-19)?"

1. Yes
2. No
3. Don't know

{IF C19Diag <> 1}
C19Had [FLIP SCALE 1…4]
"Do you think you have ever had the coronavirus (COVID-19)?"

1. Yes – definitely
2. Yes - probably
3. No – probably not
4. No – definitely not
5. Don't know

{ASK ALL}
C19Symp
"Since January 2020, have you had coronavirus (COVID-19) symptoms?

Symptoms can include a high temperature, a new continuous cough, or a loss of sense of smell or taste"

1. Yes
2. No
3. Don't know

{IF C19Symp = 1}

Stead M, *et al. BMJ Open* 2022; 11:e055085. doi: 10.1136/bmjopen-2021-055085

C19SympSev [FLIP SCALE]
"Would you say your symptoms were mild or severe?"

1. Mild
2. Severe

{ASK ALL}
C19Oth
"Do you have any friends or family who have had the coronavirus (COVID-19)?"

1. Yes
2. No

{IF C19Oth = 1}
C19OthHosp
"Have any of your friends or family had to go to hospital as a result of having the coronavirus (COVID-19)?"

1. Yes
2. No
3. Prefer not to say

{IF C19Oth = 1}
C19OthDied
"Have any of your friends or family died as a result of having the coronavirus (COVID-19)?"

1. Yes
2. No
3. Prefer not to say

{ASK ALL}
C19Imp [FLIP SCALE]
"Thinking about the impact the COVID-19 pandemic has had on different areas of your life…

How much of a negative impact, if any, would you say the COVID-19 pandemic has had on your life?"

_WEB: "Please select one answer on every row"
_TEL: "INTERVIEWER: READ OUT EACH STATEMENT AND THE ANSWER CODES. REPEAT ANSWER CODES AS REQUIRED."

1. An extremely negative impact
2. A very negative impact
3. A somewhat negative impact
4. A slightly negative impact
5. It has not had a negative impact

{ASK ALL}
C19Fut1 [FLIP SCALE 1-5]
"How likely or unlikely do you think you are to get COVID-19 in the next 6 months?

_WEB: "Please select one answer on every row"
_TEL: "INTERVIEWER: READ OUT EACH STATEMENT AND THE ANSWER CODES. REPEAT ANSWER CODES AS REQUIRED."

1. Very likely
2. Quite likely
3. Neither likely nor unlikely
4. Quite unlikely
5. Very unlikely
6. Don't know

Stead M, *et al. BMJ Open* 2022; 11:e055085. doi: 10.1136/bmjopen-2021-055085

{ASK IF C19Fut = 6}
C19Fut1DK [FLIP SCALE 1-5]
Thank you for your response.

We would really like to know your opinion on this, even if you are unsure or don't feel you have enough information.

If you had to decide, how likely or unlikely do you think you are to get COVID-19 in the next 6 months?

_WEB: "Please select one answer on every row"
_TEL: "INTERVIEWER: READ OUT EACH STATEMENT AND THE ANSWER CODES. REPEAT ANSWER CODES AS REQUIRED."

1. Very likely
2. Quite likely
3. Neither likely nor unlikely
4. Quite unlikely
5. Very unlikely
6. I really don't know

{ASK ALL}
C19Fut2 [FLIP SCALE 1-5]
If you did get COVID-19 in the next 6 months, how likely or unlikely do you think you would be to become seriously ill as a result of it?

_WEB: "Please select one answer on every row"
_TEL: "INTERVIEWER: READ OUT EACH STATEMENT AND THE ANSWER CODES. REPEAT ANSWER CODES AS REQUIRED."

1. Very likely
2. Quite likely
3. Neither likely nor unlikely
4. Quite unlikely
5. Very unlikely
6. Don't know

{ASK IF C19Fut2=6}
C19Fut2DK [FLIP SCALE 1-5]
Thank you for your response.

We would really like to know your opinion on this, even if you are unsure or don't feel you have enough information.

If you had to decide, if you did get COVID-19 in the next 6 months, how likely or unlikely do you think you would be to become seriously ill as a result of it?

_WEB: "Please select one answer on every row"
_TEL: "INTERVIEWER: READ OUT EACH STATEMENT AND THE ANSWER CODES. REPEAT ANSWER CODES AS REQUIRED."

1. Very likely
2. Quite likely
3. Neither likely nor unlikely
4. Quite unlikely
5. Very unlikely
6. I really don't know

{ASK ALL}

C19PrvDon [GRID: RANDOMISE ROWS; FLIP SCALE 1…5]
"How often do you currently do each of the following to help prevent the spread of COVID-19?"

_WEB: "Please select one answer on every row"
_TEL: "INTERVIEWER: READ OUT EACH STATEMENT AND THE ANSWER CODES. REPEAT ANSWER CODES AS REQUIRED."

GRID ROWS
1. Try to stay physically distant from other people when I am out in public
2. Avoid crowded public places
3. Wash my hands with soap and water for at least 20 seconds
4. Wear a face covering whenever in shops or on public transport
5. Obey the rules about how many people from different households can meet indoors
6. Obey the rules about how many people from different households can meet outdoors
7. Register my contact details when I visit cafes, restaurants or bars

GRID COLS
1. Always
2. Often
3. Sometimes
4. Rarely
5. Never
6. Not applicable

{ASK ALL}
C19Att [GRID: RANDOMISE ROWS; FLIP SCALE]
To what extent do you agree or disagree with each of the following statements about COVID-19?

_WEB: "Please select one answer on every row"
_TEL: "INTERVIEWER: READ OUT EACH STATEMENT AND THE ANSWER CODES. REPEAT ANSWER CODES AS REQUIRED."

GRID ROWS
1. Thinking about COVID-19 makes me feel worried
2. I am worried that I or people I care about will get sick from COVID-19
3. In general, the seriousness of COVID-19 is being exaggerated
4. COVID-19 feels like something far away from me
5. The {IF Cur_Country = -1,1: "UK"; IF Cur_Country = 2: "Scottish"; IF Cur_Country = 3: "Welsh Assembly"} Government's response to COVID-19 is doing more harm than the disease itself
6. COVID-19 is a hoax

GRID COLS
1. Strongly agree
2. Agree
3. Neither agree nor disagree
4. Disagree
5. Strongly disagree

{ASK ALL}
C19Acc [GRID: RANDOMISE ROWS; FLIP SCALE]
"How acceptable or unacceptable do you find each of the following options for addressing COVID-19 in the next 12 months?

_WEB: "Please select one answer on every row"
_TEL: "INTERVIEWER: READ OUT EACH STATEMENT AND THE ANSWER CODES. REPEAT ANSWER CODES AS REQUIRED."

GRID ROWS
1. Encouraging the general public to get vaccinated against COVID-19
2. Bringing in restrictions from time to time to stop the spread of COVID-19

Stead M, *et al. BMJ Open* 2022; 11:e055085. doi: 10.1136/bmjopen-2021-055085

3. Using test and trace systems to control the spread of COVID-19
4. Letting COVID-19 run its course through the population
5. Modifying our behaviour to live with COVID-19
6. Shielding of vulnerable people and letting everyone else get on with their lives

GRID COLS

1. Very acceptable
2. Somewhat acceptable
3. Neither acceptable nor unacceptable
4. Somewhat unacceptable
5. Very unacceptable

Table S1 Overall response rate calculation accounting for recruitment onto original panel and panel attrition.

	Wave of British Social Attitudes Survey (BSA) from which panel was recruited			
**Response to initial BSA survey**	**2018**	**2019**	**2020**	**Total 2018 to 2020**
BSA issued	10,270	7,956	42066	60,292
BSA deadwood	1,023	684	4207	5,914
BSA productive	3,879	3,224	3964	11,067
BSA response rate	42%	44%	10%	20%
**Overall response for panel recruitment**				
BSA productive	3,879	3,224	3964	11,067
Recruited to panel	2,412	2,104	3086	7,602
Panel recruitment rate	62%	65%	78%	69%
Panel deadwood	19	7	0	26
Panel lost to attrition/inactivity prior to vaccine survey	969	673	3	1645
**Panel's response to vaccine survey**				
Issued	1,424	1,424	3,083	5,931
Deadwood	1	5	0	6
Achieved	1,242	1,181	2,555	4,978
**Vaccine survey response rate**	**87%**	**83%**	**83%**	**84%**
**Overall survey response rate[a]**	**13%**	**16%**	**7%**	**9%**

[a] Response rate accounting for non-response at original point of recruitment (British Social Attitudes Survey 2018, 2019 or 2020; http://bsa.natcen.ac.uk) and panel attrition thereafter.

Stead M, et al. BMJ Open 2022; 11:e055085. doi: 10.1136/bmjopen-2021-055085

Methods S2: Non-response weights

Non-response to NatCen's probability panel surveys can occur at any one of three stages: the survey used for recruitment to the panel (the British Social Attitudes survey), the invitation to join the panel (at the end of the BSA interview) and the survey of panel members itself. The BSA survey is already weighted to adjust for non-response and we compute further weights to take account of non-response at each of the two subsequent stages. The final weights are the product of these three weights. This three-stage approach is ideal because the correlates of non-response can be different at each stage. With this system we also can optimise the use of the data available from the British Social Attitudes Survey (BSA).

These are the three weights we have computed:

1. **BSA survey weight:** the panel members were recruited from BSA 2018, 2019 and 2020. The weighting process for BSA 2020 was a little different from the other years due to the change in methodology due to the COVID-19 pandemic (using a 'push-to-web' methodology, with up to two participants in a household allowed to take part). All three years required weights to adjust for differential selection probabilities (design weights), non-response at household level (non-response weights) and weights to adjust the profile of respondents to match population estimates (calibration weights). We now describe in more detail the approaches used in 2018/19 and 2020.

   For 2018/2019, a non-response model was estimated to adjust for household level non-response. The model included region, dwelling type, percentage of owner-occupied properties in the postcode sector (grouped) and population density. The model produced a non-response weight, which was combined with the design weights (which accounted for unequal selection probabilities of households and individuals within households) to produce a composite weight. This weight was then adjusted using calibration weighting so that the profile of BSA respondents matches the British population in terms of age, sex and region.[a]

   As above, the weighting process for BSA 2020 was a little different from previous years due to the methodology used. Two non-response models were created: one to adjust for household level non-response (as in previous years), and another to account for differential response within households. The first model included (grouped) census variables measuring percentage of owner occupied properties, percentage of adults with a degree and percentage of BAME individuals in the postcode sector, plus region and the geo-demographic Output Area Classification. The second model included region, household tenure, household income (grouped), number of eligible adults and IMD tertiles. Each model produced a non-response weight and these were combined to produce a composite weight. This weight was then adjusted using calibration weighting so that the profile of BSA respondents matches the GB population in terms of age, gender, highest educational qualification, tenure and region.

2. **Panel weight:** this weight accounts for non-response at the panel recruitment stage where some people interviewed as part of the BSA survey chose not to join the panel. A logistic regression model has been used to derive the probability of response of each panel member; the panel weight is computed as the inverse of the probabilities of response. This weight adjusts the panel for non-response using the following variables: age and sex groups, region, BSA year, household type, household income, education level, internet access, ethnicity, tenure, social class group, economic activity, political party identification, and interest in politics.[b,c] The resulting panel weight has been multiplied by the BSA weights, so the panel is representative of the population.

3. **Survey weight:** this weight is to adjust the bias caused by non-response to this particular panel survey. A logistic regression model has been used to compute the probabilities of response of each participant. The panel survey weight is equal to the inverse of the probabilities of response. The initial set of predictors used to build the model was the same as for the panel weight; and at this wave the final set of variables used was also the same. Unlike the model used to calculate the panel weight, no interaction term between BSA survey year and internet access was used. As this wave of data collection was web-only, there were only a very small number of panellists (from each year of BSA) who took part in the survey but did not have access to the internet when they were interviewed for BSA. It was therefore deemed inappropriate to include the interaction term in the model.

   The final survey weight is the result of multiplying the survey weight by the compounded panel weight.

Notes:

a. More details on the BSA weight can be found at http://bsa.natcen.ac.uk/

b. The characteristics that are likely to change with time for an individual and whose distribution differed between 2018 and 2020 BSA sample have been entered into the model in interaction with BSA year.

c. More details about these variables, the question wording and the full dataset can be found at http://bsa.natcen.ac.uk/

Table S2 Doctors, nurses or other healthcare professionals – Association between trust in sources of information about COVID-19 vaccine and socio-demographic variables – (a) bivariate results and (b) multivariate logistic regression.

	**(a) Bivariate associations between socio-demographics and trusting COVID-19 vaccine info from Doctors, nurses or other healthcare professionals** % Trust completely or a great deal (weighted) χ^2 test for differences by demographics				**(b) Logistic regression of trust in COVID-19 vaccine info from Doctors, nurses or other healthcare professionals** 1 = Trust completely or a great deal (4104), 0 = Trust somewhat, very little or not at all (786)				
	**n**	**%**	**χ^2 (df)**	**P**	**N**	**AOR***	**95% CI Lower**	**95% CI Upper**	**P**
**Gender**									
Male	1957	81.7	3.020 (2)	.221	2097	ref			.526
Female	2046	79.9			2784	0.92	0.79	1.08	.331
Other	5	71.4			9	0.59	0.12	2.94	.518
**Age**									
18-29	649	78.8	53.883 (6)	<.001	459	ref			<.001
30-39 *v 18-29*	642	75.7			758	0.83	0.61	1.12	.219
40-49 *v 18-39*	629	78.4			835	0.96	0.76	1.22	.750
50-59 *v 18-49*	701	80.9			896	1.18	0.95	1.47	.133
60-69 *v 18-59*	599	84.2			1004	1.40	1.12	1.74	.003
70-79 *v 18-69*	582	89.0			761	1.72	1.32	2.24	<.001
80+ *v 18-79*	181	83.0			177	1.32	0.83	2.11	.240
**Education/Highest qualification**									
No qualifications	440	71.7	91.917 (4)	<.001	408	ref			<.001
Degree or equivalent and above	1775	85.7			2454	2.64	2.00	3.48	<.001
A levels / Vocational level 3 or equivalent	906	80.0			990	1.87	1.39	2.51	<.001
Other qual'ns below A level / Voc level 3	673	80.5			783	1.70	1.25	2.29	.001
Other qualification	210	69.1			255	0.97	0.67	1.40	.872
**Financial Status**									
Living comfortably	1121	87.2	124.251 (4)	<.001	1533	ref			<.001
Doing alright	1699	83.7			1995	0.90	0.73	1.10	.296
Just about getting by	824	72.9			959	0.61	0.48	0.77	<.001
Finding it quite difficult	247	73.3			266	0.60	0.42	0.84	.003
Finding it very difficult	116	66.7			137	0.51	0.33	0.78	.002
**Country**									
England	3499	81.7	21.523 (2)	<.001	4299	ref			.128
Scotland	321	73.0			383	0.77	0.59	1.02	.068
Wales	183	77.2			208	0.82	0.56	1.20	.308
**Urban/rural**									
Urban	3201	80.1	4.443 (1)	.035	3725	ref			
Rural	802	83.1			1165	0.97	0.80	1.18	.778
**Ethnicity**									
White British	3314	83.0	63.871 (5)	<.001	4224	ref			<.001
Any other white background	242	72.7			317	0.54	0.41	0.72	<.001
Mixed or multiple ethnic groups	64	66.0			62	0.38	0.22	0.67	<.001
Asian or Asian British	223	73.4			160	0.53	0.36	0.77	<.001
Black or Black British	70	69.3			67	0.41	0.24	0.70	.001
Other	58	71.6			60	0.56	0.30	1.03	.060
					Hosmer & Lemeshow χ^2= 10.236, df=8, p=0.249.<br>Final model χ^2=220.263, df=24, p<0.001<br>Nagelkerke = 0.075<br>Cases correctly classified: 84.0%.<br>88 cases excluded due to missing data on one or more independent variables.				

* adjusted for all other variables in the model, AOR, adjusted odds ratio; ref, reference category; 95% CI, 95% confidence interval.

Table S3 Pharmacists – Association between trust in sources of information about COVID-19 vaccine and socio-demographic variables – (a) bivariate results and (b) multivariate logistic regression.

	**(a) Bivariate associations between socio-demographics and trusting COVID-19 vaccine info from Pharmacists** % Trust completely or a great deal (weighted) χ^2 test for differences by demographics					**(b) Logistic regression of trust in COVID-19 vaccine info from Pharmacists** 1 = Trust completely or a great deal (3107), 0 = Trust somewhat, very little or not at all (1786)				
	**n**	**%**		**χ^2 (df)**	**P**	**N**	**AOR***	**95% CI Lower**	**95% CI Upper**	**P**
**Gender**										
Male	1420	59.2		1.597 [#]	.474	2098	ref			.620
Female	1548	60.4				2786	1.06	0.94	1.19	.376
Other	3	42.9				9	0.78	0.21	2.95	.715
**Age**										
18-29	461	55.9		29.783 (6)	<.001	459	ref			.092
30-39 *v 18-29*	486	57.2				759	0.98	0.77	1.24	.848
40-49 *v 18-39*	455	56.7				835	0.96	0.80	1.16	.662
50-59 *v 18-49*	518	59.7				896	1.18	0.99	1.39	.062
60-69 *v 18-59*	462	65.0				1004	1.24	1.06	1.46	.009
70-79 *v 18-69*	428	65.1				763	1.00	0.84	1.19	.972
80+ *v 18-79*	144	65.8				177	1.12	0.80	1.55	.514
**Education/Highest qualification**										
No qualifications	349	56.7		25.123 (4)	<.001	410	ref			<.001
Degree or equivalent and above	1305	63.0				2454	1.34	1.07	1.67	.012
A levels / Vocational level 3 or equivalent	680	60.1				990	1.20	0.94	1.53	.146
Other qual'ns below A level / Voc level 3	485	57.9				784	1.02	0.79	1.31	.890
Other qualification	151	49.8				255	0.82	0.59	1.13	.216
**Financial Status**										
Living comfortably	883	68.7		78.993 (4)	<.001	1533	ref			<.001
Doing alright	1220	60.0				1997	0.77	0.66	0.89	<.001
Just about getting by	607	53.6				960	0.63	0.53	0.75	<.001
Finding it quite difficult	178	52.8				266	0.62	0.47	0.82	.001
Finding it very difficult	81	46.8				137	0.51	0.35	0.73	<.001
**Country**										
England	2589	60.4		7.095 (2)	.029	4301	ref			.673
Scotland	239	54.1				384	0.91	0.73	1.13	.403
Wales	137	57.8				208	0.95	0.71	1.27	.721
**Urban/rural**										
Urban	2349	58.7		8.096 (1)	.004	3728				
Rural	615	63.7				1165	1.04	0.90	1.20	.599
**Ethnicity**										
White British	2516	62.9		91.005 (5)	<.001	4226	ref			<.001
Any other white background	157	46.7				318	0.57	0.45	0.72	<.001
Mixed or multiple ethnic groups	39	40.2				62	0.42	0.25	0.70	<.001
Asian or Asian British	142	46.6				160	0.47	0.34	0.65	<.001
Black or Black British	51	50.5				67	0.63	0.39	1.03	.067
Other	35	43.2				60	0.48	0.29	0.81	.006
						Hosmer & Lemeshow χ^2= 11.453, df=8, p=0.177.<br>Final model χ^2=157.815, df=24, p<0.001<br>Nagelkerke = .043<br>Cases correctly classified: 64.5%.<br>85 cases excluded due to missing data on one or more independent variables.				

* adjusted for all other variables in the model, AOR, adjusted odds ratio; ref, reference category; 95% CI, 95% confidence interval. [#] Fisher-Freeman-Halton Exact Test.

Table S4 The NHS – Association between trust in sources of information about COVID-19 vaccine and socio-demographic variables – (a) bivariate results and (b) multivariate logistic regression.

	**(a) Bivariate associations between socio-demographics and trusting COVID-19 vaccine info from the NHS**<br>% Trust completely or a great deal (weighted)<br>χ 2 test for differences by demographics					**(b) Logistic regression of trust in COVID-19 vaccine info from the NHS**<br>1 = Trust completely or a great deal (4115), 0 = Trust somewhat, very little or not at all (775)				
	**n**	**%**		**χ^2 (df)**	**P**	**N**	**AOR***	**95% CI Lower**	**95% CI Upper**	**P**
**Gender**										
Male	1926	80.3		1.598 (2)	.450	2097	ref			.461
Female	2055	80.2				2784	0.97	0.82	1.14	.718
Other	5	62.5				9	0.41	0.10	1.74	.225
**Age**										
18-29	609	73.9		106.785 (6)	<.001	459	ref			<.001
30-39 *v 18-29*	632	74.4				759	0.94	0.70	1.26	.672
40-49 *v 18-39*	618	77.1				834	1.05	0.84	1.32	.672
50-59 *v 18-49*	710	81.9				895	1.45	1.16	1.81	.001
60-69 *v 18-59*	609	85.7				1004	1.62	1.29	2.03	<.001
70-79 *v 18-69*	593	90.5				762	1.99	1.50	2.63	<.001
80+ *v 18-79*	186	85.3				177	1.66	0.99	2.79	.056
**Education/Highest qualification**										
No qualifications	447	72.6		60.407 (4)	<.001	410	ref			<.001
Degree or equivalent and above	1733	83.7				2452	2.39	1.80	3.16	<.001
A levels / Vocational level 3 or equivalent	909	80.4				990	1.95	1.44	2.64	<.001
Other qual'ns below A level / Voc level 3	679	81.2				783	1.78	1.30	2.43	<.001
Other qualification	211	69.6				255	0.95	0.65	1.39	.805
**Financial Status**										
Living comfortably	1136	88.3		167.221 (4)	<.001	1533	ref			<.001
Doing alright	1675	82.4				1996	0.74	0.59	0.92	.006
Just about getting by	823	72.8				958	0.47	0.37	0.60	<.001
Finding it quite difficult	248	73.6				266	0.51	0.36	0.72	<.001
Finding it very difficult	99	56.9				137	0.31	0.21	0.47	<.001
**Country**										
England	3459	80.8		6.736 (2)	.034	4298	ref			.505
Scotland	345	78.1				384	0.98	0.73	1.32	.900
Wales	177	74.7				208	0.80	0.54	1.17	.243
**Urban/rural**										
Urban	3165	79.2		14.722 (1)	<.001	3725				
Rural	817	84.7				1165	1.03	0.84	1.26	.791
**Ethnicity**										
White British	3331	83.3		126.307 (5)	<.001	4225	ref			<.001
Any other white background	224	67.3				317	0.47	0.36	0.63	<.001
Mixed or multiple ethnic groups	67	69.1				62	0.48	0.27	0.86	.014
Asian or Asian British	207	68.1				160	0.57	0.39	0.84	.004
Black or Black British	60	60.0				66	0.38	0.22	0.65	<.001
Other	57	70.4				60	0.45	0.25	0.81	.008
						Hosmer & Lemeshow χ^2= 8.677, df=8, p=0.370.<br>Final model χ^2=291.002, df=24, p<0.001<br>Nagelkerke = .099<br>Cases correctly classified: 84.3%.<br>88 cases excluded due to missing data on one or more independent variables.				

* adjusted for all other variables in the model, AOR, adjusted odds ratio; ref, reference category; 95% CI, 95% confidence interval.

Table S5 Drug companies who manufacture vaccines – Association between trust in sources of information about COVID-19 vaccine and socio-demographic variables – (a) bivariate results and (b) multivariate logistic regression.

	**(a) Bivariate associations between socio-demographics and trusting COVID-19 vaccine info from drug companies who manufacture vaccines** % Trust completely or a great deal (weighted) χ^2 test for differences by demographics					**(b) Logistic regression of trust in COVID-19 vaccine info from drug companies who manufacture vaccines** 1 = Trust completely or a great deal (1416), 0 = Trust somewhat, very little or not at all (3473)				
	**n**	**%**		**χ^2 (df)**	**P**	**N**	**AOR***	**95% CI Lower**	**95% CI Upper**	**P**
**Gender**										
Male	652	27.2		16.276[#]	<.001	2096	ref			<.001
Female	819	32.0				2784	1.28	1.12	1.45	<.001
Other	0	0.0				9	0.00	0.00	.	.999
**Age**										
18-29	264	32.0		32.180 (6)	<.001	459	ref			.030
30-39 *v 18-29*	249	29.3				759	0.91	0.71	1.18	.480
40-49 *v 18-39*	196	24.5				834	0.72	0.59	0.89	.002
50-59 *v 18-49*	226	26.1				896	0.87	0.72	1.04	.120
60-69 *v 18-59*	219	30.8				1004	0.96	0.81	1.13	.613
70-79 *v 18-69*	233	35.6				760	1.12	0.94	1.34	.204
80+ *v 18-79*	76	34.7				177	1.02	0.73	1.42	.905
**Education/Highest qualification**										
No qualifications	217	35.3		17.807 (4)	.001	408	ref			.004
Degree or equivalent and above	569	27.5				2454	0.72	0.57	0.92	.007
A levels / Vocational level 3 or equivalent	321	28.4				990	0.78	0.60	1.01	.057
Other qual'ns below A level / Voc level 3	267	31.9				783	0.97	0.75	1.26	.844
Other qualification	97	32.1				254	0.94	0.67	1.32	.732
**Financial Status**										
Living comfortably	444	34.5		20.183 (4)	<.001	1533	ref			<.001
Doing alright	565	27.8				1995	0.72	0.62	0.83	<.001
Just about getting by	313	27.7				958	0.66	0.55	0.80	<.001
Finding it quite difficult	98	29.0				266	0.74	0.55	1.00	.054
Finding it very difficult	50	29.1				137	0.55	0.36	0.85	.007
**Country**										
England	1259	29.4		2.325 (2)	.313	4297	ref			.842
Scotland	130	29.3				384	1.00	0.79	1.26	.996
Wales	81	34.0				208	1.10	0.81	1.49	.559
**Urban/rural**										
Urban	1187	29.7		.092 (1)	.762	3724				
Rural	282	29.2				1165	0.99	0.85	1.15	.868
**Ethnicity**										
White British	1234	30.9		29.028 (5)	<.001	4223	ref			.012
Any other white background	70	20.9				318	0.63	0.48	0.84	.002
Mixed or multiple ethnic groups	20	20.6				62	0.92	0.52	1.62	.769
Asian or Asian British	83	27.2				160	0.74	0.50	1.08	.119
Black or Black British	35	34.7				67	1.11	0.64	1.91	.714
Other	12	15.2				59	0.55	0.28	1.07	.078
						Hosmer & Lemeshow χ^2= 9.180, df=8, p=0.327. Final model χ^2=96.401, df=24, p<0.001 Nagelkerke = .028 Cases correctly classified: 71.0%. 89 cases excluded due to missing data on one or more independent variables.				

* adjusted for all other variables in the model, AOR, adjusted odds ratio; ref, reference category; 95% CI, 95% confidence interval. [#] Fisher-Freeman-Halton Exact Test.

Table S6 The UK Government – Association between trust in sources of information about COVID-19 vaccine and socio-demographic variables – (a) bivariate results and (b) multivariate logistic regression.

	**(a) Bivariate associations between socio-demographics and trusting COVID-19 vaccine info from the UK Government** % Trust completely or a great deal (weighted) χ^2 test for differences by demographics				**(b) Logistic regression of trust in COVID-19 vaccine info from the UK Government** 1 = Trust completely or a great deal (2279), 0 = Trust somewhat, very little or not at all (2607)				
	**n**	**%**	**χ^2 (df)**	**P**	**N**	**AOR***	**95% CI Lower**	**95% CI Upper**	**P**
**Gender**									
Male	1114	46.6	10.393#	.005	2096	ref			.012
Female	1080	42.3			2781	0.84	0.75	0.95	.004
Other	2	25.0			9	0.47	0.09	2.37	.363
**Age**									
18-29	261	31.9	182.080 (6)	<.001	458	ref			<.001
30-39 *v 18-29*	290	34.2			758	1.14	0.89	1.47	.289
40-49 *v 18-39*	332	41.5			834	1.37	1.14	1.65	<.001
50-59 *v 18-49*	416	48.0			895	1.67	1.41	1.97	<.001
60-69 *v 18-59*	373	52.8			1003	1.46	1.25	1.70	<.001
70-79 *v 18-69*	383	58.6			762	1.65	1.40	1.96	<.001
80+ *v 18-79*	124	57.1			176	1.54	1.13	2.11	.007
**Education/Highest qualification**									
No qualifications	269	43.7	2.856 (4)	.582	410	ref			.439
Degree or equivalent and above	924	44.8			2450	0.95	0.76	1.18	.622
A levels / Vocational level 3 or equivalent	482	42.6			990	1.04	0.82	1.33	.733
Other qual'ns below A level / Voc level 3	386	46.2			783	1.07	0.83	1.37	.601
Other qualification	134	45.1			253	1.14	0.83	1.58	.422
**Financial Status**									
Living comfortably	704	54.8	94.512 (4)	<.001	1533	ref			<.001
Doing alright	891	43.9			1994	0.76	0.66	0.87	<.001
Just about getting by	415	36.9			957	0.54	0.45	0.64	<.001
Finding it quite difficult	124	36.9			266	0.55	0.42	0.73	<.001
Finding it very difficult	60	36.1			136	0.40	0.27	0.59	<.001
**Country**									
England	1928	45.1	19.887 (2)	<.001	4295	ref			.003
Scotland	151	34.5			383	0.70	0.56	0.88	.002
Wales	114	48.3			208	1.18	0.89	1.58	.253
**Urban/rural**									
Urban	1741	43.8	2.886 (1)	.089	3721				
Rural	451	46.8			1165	1.00	0.87	1.15	.969
**Ethnicity**									
White British	1832	45.9	35.180 (5)	<.001	4224	ref			.074
Any other white background	104	31.2			317	0.70	0.55	0.90	.005
Mixed or multiple ethnic groups	31	32.0			62	0.73	0.43	1.26	.258
Asian or Asian British	126	42.6			158	1.14	0.82	1.59	.430
Black or Black British	40	40.0			66	1.07	0.64	1.77	.803
Other	31	39.2			59	1.03	0.60	1.76	.910
					Hosmer & Lemeshow χ^2= 9.412, df=8, p=0.309.<br>Final model χ^2=258.301, df=24, p<0.001<br>Nagelkerke = .069<br>Cases correctly classified: 60.3%.<br>92 cases excluded due to missing data on one or more independent variables.				

* adjusted for all other variables in the model, AOR, adjusted odds ratio; ref, reference category; 95% CI, 95% confidence interval. # Fisher-Freeman-Halton Exact Test.

Table S7 The Scottish/Welsh Government – Association between trust in sources of information about COVID-19 vaccine and socio-demographic variables – (a) bivariate results and (b) multivariate logistic regression.

	**(a) Bivariate associations between socio-demographics and trusting COVID-19 vaccine info from the Scottish/Welsh Government** % Trust completely or a great deal (weighted) χ^2 test for differences by demographics					**(b) Logistic regression of trust in COVID-19 vaccine info from the Scottish/Welsh Government** 1 = Trust completely or a great deal (289), 0 = Trust somewhat, very little or not at all (297)				
	**n**	**%**		**χ^2 (df)**	**P**	**N**	**AOR***	**95% CI Lower**	**95% CI Upper**	**P**
**Gender**										
Male	173	49.0		3.935 (1)	.047	274	ref			
Female	132	41.4				312	0.89	0.63	1.25	.491
**Age**										
18-29	61	50.8		8.885 (6)	.180	63	ref			.249
30-39 *v 18-29*	45	44.1				93	1.27	0.65	2.48	.485
40-49 *v 18-39*	36	36.7				95	0.87	0.51	1.49	.617
50-59 *v 18-49*	75	52.1				117	1.66	1.03	2.67	.037
60-69 *v 18-59*	46	46.5				113	1.06	0.67	1.68	.790
70-79 *v 18-69*	32	43.8				81	0.78	0.46	1.32	.353
80+ *v 18-79*	10	33.3				24	0.63	0.26	1.50	.297
**Education/Highest qualification**										
No qualifications	29	27.9		31.212 (4)	<.001	52	ref			.042
Degree or equivalent and above	144	56.7				280	2.18	1.12	4.23	.021
A levels / Vocational level 3 or equivalent	79	47.3				135	1.95	0.97	3.95	.062
Other qual'ns below A level / Voc level 3	34	36.6				81	1.27	0.60	2.69	.538
Other qualification	18	35.3				38	1.03	0.42	2.53	.941
**Financial Status**										
Living comfortably	84	56.0		8.356 (4)	.079	167	ref			.153
Doing alright	118	42.4				256	0.69	0.46	1.05	.081
Just about getting by	71	43.3				114	0.62	0.37	1.05	.074
Finding it quite difficult	22	41.5				34	0.49	0.22	1.09	.078
Finding it very difficult	11	45.8				15	0.34	0.10	1.16	.083
**Country**										
Scotland	206	46.9		1.208 (1)	.272	381	ref			
Wales	99	42.5				205	1.09	0.76	1.57	.642
**Urban/rural**										
Urban	233	46.9		1.719 (1)	.190	409				
Rural	72	41.1				177	0.62	0.42	0.91	.015
**Ethnicity**										
White British	270	45.3		.147 (1)	.702	535	ref			
Other than white British	32	47.8				51	1.03	0.55	1.92	.932
						Hosmer & Lemeshow χ^2= 12.017, df=8, p=0.150.<br>Final model χ^2=35.151, df=18, p=0.009<br>Nagelkerke = .078<br>Cases correctly classified: 61.4%.<br>16 cases excluded due to missing data on one or more independent variables.				

* adjusted for all other variables in the model, AOR, adjusted odds ratio; ref, reference category; 95% CI, 95% confidence interval. [#] Fisher-Freeman-Halton Exact Test.

Table S8 Scientific and medical advisers – Association between trust in sources of information about COVID-19 vaccine and socio-demographic variables – (a) bivariate results and (b) multivariate logistic regression.

	**(a) Bivariate associations between socio-demographics and trusting COVID-19 vaccine info from scientific and medical advisers** % Trust completely or a great deal (weighted) χ^2 test for differences by demographics					**(b) Logistic regression of trust in COVID-19 vaccine info from scientific and medical advisers** 1 = Trust completely or a great deal (4008), 0 = Trust somewhat, very little or not at all (884)				
	**n**	**%**		**χ^2 (df)**	**P**	**N**	**AOR***	**95% CI Lower**	**95% CI Upper**	**P**
**Gender**										
Male	1885	78.6		0.302 (2)	.860	2097	ref			.544
Female	2006	78.2				2786	1.08	0.93	1.26	.320
Other	5	71.4				9	0.71	0.14	3.51	.674
**Age**										
18-29	644	78.2		11.885 (6)	.065	459	ref			.850
30-39 *v 18-29*	643	75.6				759	0.86	0.63	1.17	.323
40-49 *v 18-39*	631	78.6				835	1.06	0.84	1.34	.640
50-59 *v 18-49*	677	78.1				896	1.09	0.88	1.36	.426
60-69 *v 18-59*	572	80.5				1004	1.03	0.84	1.26	.785
70-79 *v 18-69*	540	82.2				763	1.05	0.84	1.32	.654
80+ *v 18-79*	166	76.1				176	1.10	0.72	1.68	.650
**Education/Highest qualification**										
No qualifications	389	63.1		147.739 (4)	<.001	410	ref			<.001
Degree or equivalent and above	1743	84.1				2453	3.21	2.50	4.13	<.001
A levels / Vocational level 3 or equivalent	905	80.0				990	2.70	2.04	3.55	<.001
Other qual'ns below A level / Voc level 3	653	77.8				784	1.98	1.51	2.61	<.001
Other qualification	205	67.7				255	1.23	0.87	1.73	.245
**Financial Status**										
Living comfortably	1110	86.4		154.081 (4)	<.001	1532	ref			<.001
Doing alright	1640	80.6				1997	0.65	0.53	0.80	<.001
Just about getting by	797	70.4				960	0.46	0.36	0.58	<.001
Finding it quite difficult	249	73.9				266	0.51	0.36	0.72	<.001
Finding it very difficult	97	55.7				137	0.32	0.21	0.48	<.001
**Country**										
England	3375	78.8		3.260 (2)	.196	4300	ref			.068
Scotland	341	77.0				384	1.03	0.77	1.37	.843
Wales	176	74.3				208	0.67	0.47	0.94	.022
**Urban/rural**										
Urban	3108	77.7		5.368 (1)	.021	3727				
Rural	783	81.1				1165	1.00	0.83	1.20	.978
**Ethnicity**										
White British	3236	80.9		87.036 (5)	<.001	4225	ref			<.001
Any other white background	244	72.8				318	0.56	0.42	0.75	<.001
Mixed or multiple ethnic groups	68	70.8				62	0.51	0.28	0.92	.025
Asian or Asian British	203	66.6				160	0.46	0.32	0.67	<.001
Black or Black British	57	56.4				67	0.31	0.19	0.53	<.001
Other	53	65.4				60	0.43	0.24	0.76	.004
						Hosmer & Lemeshow χ^2= 5.496, df=8, p=0.704.<br>Final model χ^2=268.594, df=24, p<0.001<br>Nagelkerke = .087<br>Cases correctly classified: 82.1%.<br>86 cases excluded due to missing data on one or more independent variables.				

* adjusted for all other variables in the model, AOR, adjusted odds ratio; ref, reference category; 95% CI, 95% confidence interval.

Stead M, *et al. BMJ Open* 2022; 11:e055085. doi: 10.1136/bmjopen-2021-055085

Table S9 The World Health Organisation (WHO) – Association between trust in sources of information about COVID-19 vaccine and socio-demographic variables – (a) bivariate results and (b) multivariate logistic regression.

	**(a) Bivariate associations between socio-demographics and trusting COVID-19 vaccine info from The World Health Organisation (WHO)**<br>% Trust completely or a great deal (weighted)<br>χ^2 test for differences by demographics					**(b) Logistic regression of trust in COVID-19 vaccine info from The World Health Organisation (WHO)**<br>1 = Trust completely or a great deal (3423), 0 = Trust somewhat, very little or not at all (1468)				
	**n**	**%**		**χ^2 (df)**	**P**	**N**	**AOR***	**95% CI Lower**	**95% CI Upper**	**P**
**Gender**										
Male	1523	63.7		23.303[#]	<.001	2097	ref			<.001
Female	1797	70.1				2785	1.49	1.31	1.69	<.001
Other	5	71.4				9	1.92	0.39	9.42	.420
**Age**										
18-29	556	68.1		5.005 (6)	.543	458	ref			.177
30-39 *v 18-29*	549	64.5				760	0.84	0.65	1.10	.203
40-49 *v 18-39*	538	67.1				835	0.98	0.81	1.20	.882
50-59 *v 18-49*	593	68.4				896	1.15	0.96	1.39	.126
60-69 *v 18-59*	484	68.1				1004	0.89	0.76	1.06	.189
70-79 *v 18-69*	450	68.6				762	0.87	0.73	1.04	.127
80+ *v 18-79*	140	64.8				176	0.86	0.62	1.20	.366
**Education/Highest qualification**										
No qualifications	351	56.9		75.592 (4)	<.001	410	ref			<.001
Degree or equivalent and above	1490	72.3				2452	1.73	1.38	2.18	<.001
A levels / Vocational level 3 or equivalent	758	67.0				990	1.39	1.08	1.77	.010
Other qual’ns below A level / Voc level 3	561	66.9				784	1.36	1.06	1.76	.017
Other qualification	166	54.6				255	0.88	0.64	1.21	.428
**Financial Status**										
Living comfortably	953	74.2		67.486 (4)	<.001	1533	ref			<.001
Doing alright	1384	68.2				1995	0.78	0.66	0.91	.002
Just about getting by	691	61.0				960	0.60	0.50	0.73	<.001
Finding it quite difficult	204	60.4				266	0.56	0.42	0.75	<.001
Finding it very difficult	92	54.8				137	0.45	0.31	0.65	<.001
**Country**										
England	2880	67.4		2.028 (2)	.363	-				
Scotland	283	64.0				-	-	-	-	-
Wales	160	67.5				-	-	-	-	-
**Urban/rural**										
Urban	2663	66.7		1.288 (1)	.256	3727				
Rural	660	68.6				1164	0.99	0.85	1.15	.897
**Ethnicity**										
White British	2737	68.5		30.713 (5)	<.001	4224	ref			<.001
Any other white background	223	66.4				318	0.75	0.58	0.96	.023
Mixed or multiple ethnic groups	59	61.5				62	0.63	0.37	1.07	.089
Asian or Asian British	171	57.2				160	0.64	0.46	0.90	.010
Black or Black British	55	53.9				67	0.44	0.27	0.72	.001
Other	45	56.3				60	0.61	0.36	1.04	.068
						Hosmer & Lemeshow χ^2= 14.933, df=8, p=0.060.<br>Final model χ^2=172.240, df=22, p<0.001<br>Nagelkerke = .049<br>Cases correctly classified: 70.0%.<br>87 cases excluded due to missing data on one or more independent variables.				

* adjusted for all other variables in the model, AOR, adjusted odds ratio; ref, reference category; 95% CI, 95% confidence interval. [#] Fisher-Freeman-Halton Exact Test. Country was excluded from the logistic regression to achieve model fit.

Table S10 The media (e.g. newspapers, magazines, television, radio) – Association between trust in sources of information about COVID-19 vaccine and socio-demographic variables – (a) bivariate results and (b) multivariate logistic regression.

	**(a) Bivariate associations between socio-demographics and trusting COVID-19 vaccine info from the media** % Trust completely or a great deal (weighted) χ^2 test for differences by demographics					**(b) Logistic regression of trust in COVID-19 vaccine info from the media** 1 = Trust completely or a great deal (361), 0 = Trust somewhat, very little or not at all (4530)				
	**n**	**%**		**χ^2 (df)**	**P**	**N**	**AOR***	**95% CI Lower**	**95% CI Upper**	**P**
**Gender**										
Male	176	7.3		2.193 (2)	.334	2097				.575
Female	213	8.3				2785	1.13	0.90	1.41	.293
Other	0	0.0				9	0.00	0.00		.999
**Age**										
18-29	57	6.9		39.450 (6)	<.001	459				.003
30-39 *v 18-29*	57	6.7				759	0.84	0.52	1.35	.462
40-49 *v 18-39*	52	6.5				834	1.04	0.72	1.49	.848
50-59 *v 18-49*	58	6.7				895	1.08	0.78	1.50	.629
60-69 *v 18-59*	49	6.9				1004	1.05	0.77	1.42	.761
70-79 *v 18-69*	85	12.9				763	1.75	1.32	2.33	<.001
80+ *v 18-79*	29	13.3				177	1.74	1.07	2.83	.024
**Education/Highest qualification**										
No qualifications	77	12.5		34.152 (4)	<.001	409				.005
Degree or equivalent and above	153	7.4				2454	0.63	0.44	0.90	.011
A levels / Vocational level 3 or equivalent	58	5.1				990	0.44	0.29	0.68	<.001
Other qual'ns below A level / Voc level 3	79	9.4				784	0.72	0.48	1.07	.102
Other qualification	21	7.0				254	0.56	0.31	1.00	.051
**Financial Status**										
Living comfortably	105	8.2		34.041 (4)	<.001	1533				.671
Doing alright	122	6.0				1996	0.90	0.69	1.16	.413
Just about getting by	99	8.8				959	1.01	0.73	1.39	.974
Finding it quite difficult	33	9.8				266	1.22	0.75	1.98	.430
Finding it very difficult	30	17.2				137	0.79	0.37	1.68	.535
**Country**										
England	336	7.8		.515 (2)	.773	4300				.457
Scotland	32	7.3				383	0.79	0.51	1.24	.313
Wales	21	8.8				208	1.19	0.71	2.00	.499
**Urban/rural**										
Urban	323	8.1		1.957 (1)	.162	3726				
Rural	65	6.7				1165	0.91	0.70	1.19	.486
**Ethnicity**										
White British	296	7.4		6.645 (5)	.248	4226				.073
Any other white background	30	9.0				318	1.55	1.03	2.32	.035
Mixed or multiple ethnic groups	6	6.2				62	1.31	0.52	3.33	.570
Asian or Asian British	30	9.9				160	1.73	1.02	2.94	.043
Black or Black British	12	11.9				66	1.84	0.82	4.12	.139
Other	8	10.3				59	1.62	0.68	3.85	.274
						Hosmer & Lemeshow χ^2= 2.359, df=8, p=0.968.<br>Final model χ^2=54.051, df=24, p<0.001<br>Nagelkerke = .027<br>Cases correctly classified: 92.6%.<br>87 cases excluded due to missing data on one or more independent variables.				

* adjusted for all other variables in the model, AOR, adjusted odds ratio; ref, reference category; 95% CI, 95% confidence interval.

Table S11 Social media – Association between trust in sources of information about COVID-19 vaccine and socio-demographic variables – (a) bivariate results and (b) multivariate logistic regression.

	**(a) Bivariate associations between socio-demographics and trusting COVID-19 vaccine info from social media**<br>% Trust completely or a great deal (weighted)<br>χ^2 test for differences by demographics					**(b) Logistic regression of trust in COVID-19 vaccine info from social media**<br>1 = Trust completely or a great deal (95), 0 = Trust somewhat, very little or not at all (4792)				
	**n**	**%**		**χ^2 (df)**	**P**	**N**	**AOR***	**95% CI Lower**	**95% CI Upper**	**P**
**Gender**										
Male	63	2.6		0.284 (2)	.868	2094	ref			.845
Female	71	2.8				2784	1.13	0.74	1.73	.561
Other	0	0.0				9	0.00	0.00		.999
**Age**										
18-29	31	3.8		12.626 (6)	.049	459	ref			.634
30-39 *v 18-29*	25	2.9				759	0.55	0.24	1.22	.139
40-49 *v 18-39*	22	2.7				835	0.89	0.47	1.67	.713
50-59 *v 18-49*	13	1.5				896	0.85	0.47	1.55	.596
60-69 *v 18-59*	13	1.8				1003	0.71	0.39	1.29	.259
70-79 *v 18-69*	19	2.9				761	1.11	0.62	1.97	.727
80+ *v 18-79*	9	4.2				174	1.26	0.49	3.25	.631
**Education/Highest qualification**										
No qualifications	31	5.1		24.978 (4)	<.001	409	ref			<.001
Degree or equivalent and above	44	2.1				2452	0.24	0.13	0.44	<.001
A levels / Vocational level 3 or equivalent	18	1.6				989	0.32	0.16	0.64	.001
Other qual'ns below A level / Voc level 3	32	3.8				782	0.50	0.26	0.94	.031
Other qualification	8	2.6				255	0.46	0.18	1.18	.106
**Financial Status**										
Living comfortably	34	2.6		26.413 (4)	<.001	1531	ref			.522
Doing alright	38	1.9				1994	0.73	0.43	1.24	.241
Just about getting by	35	3.1				959	0.99	0.55	1.79	.976
Finding it quite difficult	12	3.6				266	1.35	0.60	3.04	.473
Finding it very difficult	14	8.1				137	0.81	0.23	2.84	.746
**Country**										
England	118	2.8		.404(2)	.817	4295	ref			.215
Scotland	10	2.3				384	0.40	0.12	1.27	.120
Wales	6	2.5				208	1.42	0.56	3.58	.463
**Urban/rural**										
Urban	122	3.1		9.660(1)	.002	3724				
Rural	12	1.2				1163	0.57	0.31	1.05	.071
**Ethnicity**										
White British	94	2.4		17.781#	.002	4220	ref			.326
Any other white background	7	2.1				318	1.00	0.39	2.53	.994
Mixed or multiple ethnic groups	4	4.1				62	2.52	0.75	8.45	.134
Asian or Asian British	14	4.6				160	1.85	0.77	4.45	.172
Black or Black British	9	8.8				69	2.57	0.76	8.64	.128
Other	1	1.2				60	0.83	0.11	6.20	.855
						Hosmer & Lemeshow χ^2= 5.858, df=8, p=0.663.<br>Final model χ^2=46.839, df=24, p=0.004<br>Nagelkerke = .055<br>Cases correctly classified: 98.1%.<br>91 cases excluded due to missing data on one or more independent variables.				

* adjusted for all other variables in the model, AOR, adjusted odds ratio; ref, reference category; 95% CI, 95% confidence interval. # Fisher-Freeman-Halton Exact Test.

Table S12 Celebrities and social media influencers – Association between trust in sources of information about COVID-19 vaccine and socio-demographic variables – (a) bivariate results and (b) multivariate logistic regression.

	**(a) Bivariate associations between socio-demographics and trusting COVID-19 vaccine info from celebrities and social media influencers** % Trust completely or a great deal (weighted) χ^2 test for differences by demographics					**(b) Logistic regression of trust in COVID-19 vaccine info from celebrities and social media influencers** 1 = Trust completely or a great deal (95), 0 = Trust somewhat, very little or not at all (4795)				
	**n**	**%**		**χ^2 (df)**	**P**	**N**	**AOR***	**95% CI Lower**	**95% CI Upper**	**P**
**Gender**										
Male	57	2.4		1.430 (2)	.489	2097	ref			.996
Female	74	2.9				2784	0.98	0.65	1.49	.928
Other	0	0.0				9	0.00	0.00		.999
**Age**										
18-29	24	2.9		19.156 (6)	.004	459	ref			.348
30-39 *v 18-29*	25	3.0				758	0.90	0.37	2.21	.818
40-49 *v 18-39*	23	2.9				835	1.11	0.58	2.14	.744
50-59 *v 18-49*	18	2.1				895	1.28	0.73	2.25	.390
60-69 *v 18-59*	9	1.3				1004	0.65	0.34	1.25	.192
70-79 *v 18-69*	17	2.6				762	1.26	0.70	2.28	.441
80+ *v 18-79*	14	6.4				177	2.18	0.96	4.98	.064
**Education/Highest qualification**										
No qualifications	35	5.7		58.886 (4)	<.001	409	ref			.002
Degree or equivalent and above	32	1.5				2453	0.31	0.17	0.58	<.001
A levels / Vocational level 3 or equivalent	13	1.1				990	0.31	0.15	0.65	.002
Other qual'ns below A level / Voc level 3	41	4.9				783	0.59	0.31	1.11	.103
Other qualification	10	3.3				255	0.57	0.24	1.40	.221
**Financial Status**										
Living comfortably	23	1.8		53.820 (4)	<.001	1533	ref			.022
Doing alright	27	1.3				1995	1.01	0.56	1.82	.975
Just about getting by	59	5.2				959	2.08	1.13	3.80	.018
Finding it quite difficult	11	3.3				266	2.47	1.08	5.64	.032
Finding it very difficult	10	5.7				137	1.86	0.60	5.77	.284
**Country**										
England	117	2.7		11.948 (2)	.003	4299	ref			.028
Scotland	3	0.7				383	0.40	0.13	1.29	.127
Wales	12	5.0				208	2.26	1.06	4.82	.036
**Urban/rural**										
Urban	119	3.0		9.096 (1)	.003	3725				
Rural	12	1.2				1165	0.64	0.36	1.14	.133
**Ethnicity**										
White British	94	2.4		14.208#	.008	4224	ref			.574
Any other white background	11	3.3				318	1.62	0.75	3.47	.217
Mixed or multiple ethnic groups	1	1.0				62	0.84	0.11	6.27	.866
Asian or Asian British	10	3.3				160	1.58	0.61	4.09	.346
Black or Black British	9	8.9				66	2.36	0.70	7.94	.166
Other	1	1.2				60	0.94	0.13	7.04	.956
						Hosmer & Lemeshow χ^2= 9.111, df=8, p=0.333.<br>Final model χ^2=57.132, df=24, p<0.001<br>Nagelkerke = .067<br>Cases correctly classified: 98.1%.<br>88 cases excluded due to missing data on one or more independent variables.				

* adjusted for all other variables in the model, AOR, adjusted odds ratio; ref, reference category; 95% CI, 95% confidence interval.

Stead M, *et al. BMJ Open* 2022; 11:e055085. doi: 10.1136/bmjopen-2021-055085

Table S13 Family and friends – Association between trust in sources of information about COVID-19 vaccine and socio-demographic variables – (a) bivariate results and (b) multivariate logistic regression.

	**(a) Bivariate associations between socio-demographics and trusting COVID-19 vaccine info from family and friends**<br>% Trust completely or a great deal (weighted)<br>χ^2 test for differences by demographics				**(b) Logistic regression of trust in COVID-19 vaccine info from family and friends**<br>1 = Trust completely or a great deal (1139), 0 = Trust somewhat, very little or not at all (3752)				
	**n**	**%**	**χ^2 (df)**	**P**	**N**	**AOR***	**95% CI Lower**	**95% CI Upper**	**P**
**Gender**									
Male	557	23.2	4.455 (2)	.108	2098	ref			.053
Female	660	25.8			2784	1.17	1.01	1.34	.030
Other	2	28.6			9	2.39	0.58	9.85	.227
**Age**									
18-29	140	17.0	109.226 (6)	<.001	459	ref			<.001
30-39 *v 18-29*	194	22.8			759	1.28	0.94	1.73	.112
40-49 *v 18-39*	186	23.3			834	1.18	0.94	1.47	.157
50-59 *v 18-49*	182	21.0			896	0.93	0.76	1.15	.504
60-69 *v 18-59*	176	24.8			1004	1.11	0.92	1.33	.283
70-79 *v 18-69*	233	35.6			762	1.85	1.54	2.23	<.001
80+ *v 18-79*	90	41.1			177	2.33	1.69	3.20	<.001
**Education/Highest qualification**									<.001
No qualifications	203	33.0	70.692 (4)	<.001	410	ref			<.001
Degree or equivalent and above	403	19.4			2454	0.61	0.47	0.77	<.001
A levels / Vocational level 3 or equivalent	271	23.9			990	0.81	0.62	1.05	.112
Other qual'ns below A level / Voc level 3	254	30.4			783	0.99	0.76	1.29	.925
Other qualification	86	28.5			254	0.96	0.68	1.36	.812
**Financial Status**									
Living comfortably	330	25.7	6.383 (4)	.172	1533	ref			.667
Doing alright	470	23.1			1997	0.94	0.79	1.10	.424
Just about getting by	300	26.6			958	1.00	0.81	1.22	.976
Finding it quite difficult	81	24.0			266	1.03	0.75	1.42	.856
Finding it very difficult	38	21.8			137	0.75	0.47	1.18	.212
**Country**									
England	1051	24.6	13.592 (2)	.001	4299	ref			.012
Scotland	89	20.1			384	1.00	0.77	1.29	.976
Wales	78	32.9			208	1.59	1.17	2.17	.003
**Urban/rural**									
Urban	979	24.5	.029 (1)	.866	3726				
Rural	239	24.8			1165	0.91	0.77	1.07	.237
**Ethnicity**									
White British	1005	25.1	36.523 (5)	<.001	4226	ref			.029
Any other white background	50	15.0			317	0.79	0.58	1.07	.128
Mixed or multiple ethnic groups	15	15.5			62	0.76	0.38	1.51	.428
Asian or Asian British	100	32.8			160	1.52	1.06	2.18	.024
Black or Black British	27	26.7			67	1.39	0.79	2.46	.250
Other	12	15.2			59	0.53	0.24	1.18	.122
					Hosmer & Lemeshow χ^2= 6.067, df=8, p=0.640.<br>Final model χ^2=153.732, df=24, p<0.001<br>Nagelkerke = .047<br>Cases correctly classified: 76.7%.<br>87 cases excluded due to missing data on one or more independent variables.				

* adjusted for all other variables in the model, AOR, adjusted odds ratio; ref, reference category; 95% CI, 95% confidence interval.

Table S14 Faith or community leaders − Association between trust in sources of information about COVID-19 vaccine and socio-demographic variables – (a) bivariate results and (b) multivariate logistic regression.

	**(a) Bivariate associations between socio-demographics and trusting COVID-19 vaccine info from faith and community leaders** % Trust completely or a great deal (weighted) χ^2 test for differences by demographics					**(b) Logistic regression of trust in COVID-19 vaccine info from faith and community leaders** 1 = Trust completely or a great deal (161), 0 = Trust somewhat, very little or not at all (4724)				
	**n**	**%**		**χ^2 (df)**	**P**	**N**	**AOR***	**95% CI Lower**	**95% CI Upper**	**P**
**Gender**										
Male	118	4.9		17.452 (2)	<.001	2095	ref			.006
Female	135	5.3				2781	1.19	0.86	1.66	.294
Other	3	37.5				9	14.06	2.67	73.92	.002
**Age**										
18-29	47	5.7		20.879 (6)	.002	459	ref			.041
30-39 *v 18-29*	46	5.4				758	0.95	0.47	1.93	.880
40-49 *v 18-39*	31	3.9				834	1.11	0.65	1.89	.710
50-59 *v 18-49*	46	5.3				895	1.70	1.09	2.65	.020
60-69 *v 18-59*	20	2.8				1003	0.98	0.61	1.58	.933
70-79 *v 18-69*	42	6.4				760	1.62	1.03	2.55	.038
80+ *v 18-79*	20	9.2				176	2.28	1.15	4.56	.019
**Education/Highest qualification**										
No qualifications	57	9.3		37.137 (4)	<.001	407	ref			.011
Degree or equivalent and above	76	3.7				2451	0.42	0.25	0.71	<.001
A levels / Vocational level 3 or equivalent	51	4.5				990	0.57	0.33	0.99	.048
Other qual'ns below A level / Voc level 3	58	6.9				782	0.75	0.44	1.28	.290
Other qualification	15	4.9				255	0.50	0.22	1.14	.098
**Financial Status**										
Living comfortably	59	4.6		39.487 (4)	<.001	1530	ref			.042
Doing alright	71	3.5				1997	0.91	0.59	1.39	.663
Just about getting by	84	7.5				955	1.46	0.92	2.33	.110
Finding it quite difficult	20	5.9				266	2.05	1.09	3.84	.025
Finding it very difficult	20	11.5				137	1.44	0.60	3.44	.417
**Country**										
England	239	5.6		12.569 (2)	.002	4294	ref			.592
Scotland	11	2.5				383	0.69	0.33	1.43	.316
Wales	5	2.1				208	1.07	0.46	2.48	.877
**Urban/rural**										
Urban	229	5.7		13.640 (1)	<.001	3722				
Rural	27	2.8				1163	0.66	0.42	1.03	.068
**Ethnicity**										
White British	163	4.1		152.072(5)	<.001	4219	ref			<.001
Any other white background	12	3.6				318	1.22	0.60	2.46	.583
Mixed or multiple ethnic groups	3	3.1				62	2.59	0.90	7.42	.077
Asian or Asian British	59	19.6				159	4.82	2.76	8.42	<.001
Black or Black British	12	11.9				67	4.52	2.04	9.99	<.001
Other	2	2.5				60	1.37	0.32	5.77	.669
						Hosmer & Lemeshow χ^2= 11.202, df=8, p=0.191. Final model χ^2=87.282, df=24, p<0.001 Nagelkerke = .070 Cases correctly classified: 96.7%. 93 cases excluded due to missing data on one or more independent variables.				

* adjusted for all other variables in the model, AOR, adjusted odds ratio; ref, reference category; 95% CI, 95% confidence interval.

Stead M, *et al. BMJ Open* 2022; 11:e055085. doi: 10.1136/bmjopen-2021-055085
