# Supplementary material for: National survey of attitudes towards and intentions to vaccinate against COVID-19: implications for communications
Source: BMJ Open. 2021 Oct 27;11(10):e055085. doi: 10.1136/bmjopen-2021-055085 (PMC8557244; doi:10.1136/bmjopen-2021-055085)
Supplement: Author's manuscript [file bmjopen-2021-055085.draft_revisions.pdf]

BMJ Open is committed to open peer review. As part of this commitment we make the peer review history of every article we publish publicly available.

When an article is published we post the peer reviewers' comments and the authors' responses online. We also post the versions of the paper that were used during peer review. These are the versions that the peer review comments apply to.

The versions of the paper that follow are the versions that were submitted during the peer review process. They are not the versions of record or the final published versions. They should not be cited or distributed as the published version of this manuscript.

BMJ Open is an open access journal and the full, final, typeset and author-corrected version of record of the manuscript is available on our site with no access controls, subscription charges or pay-per-view fees (http://bmjopen.bmj.com).

If you have any questions on BMJ Open's open peer review process please email info.bmjopen@bmj.com

BMJ Open

A national survey of attitudes towards and intentions to vaccinate against COVID-19: implications for communications

Journal:	*BMJ Open*
Manuscript ID	bmjopen-2021-055085
Article Type:	Original research
Date Submitted by the Author:	01-Jul-2021
Complete List of Authors:	Stead, Martine; University of Stirling Institute for Social Marketing and Health<br>Jessop, Curtis; NatCen Social Research<br>Angus, Kathryn; University of Stirling Institute for Social Marketing and Health<br>Bedford, Helen; UCL Great Ormond Street Institute of Child Health Population Policy and Practice<br>Ussher, Michael; University of Stirling Institute for Social Marketing and Health; St George's University of London Population Health Research Institute<br>Ford, Allison; University of Stirling Institute for Social Marketing and Health<br>Eadie, Douglas; University of Stirling Institute for Social Marketing and Health<br>MacGregor, Andy; NatCen Social Research<br>Hunt, Kate; University of Stirling Institute for Social Marketing and Health<br>MacKintosh, Anne Marie; University of Stirling Institute for Social Marketing and Health
Keywords:	COVID-19, IMMUNOLOGY, Epidemiology < INFECTIOUS DISEASES, Public health < INFECTIOUS DISEASES, PUBLIC HEALTH

SCHOLARONE™
Manuscripts

I, the Submitting Author has the right to grant and does grant on behalf of all authors of the Work (as defined in the below author licence), an exclusive licence and/or a non-exclusive licence for contributions from authors who are: i) UK Crown employees; ii) where BMJ has agreed a CC-BY licence shall apply, and/or iii) in accordance with the terms applicable for US Federal Government officers or employees acting as part of their official duties; on a worldwide, perpetual, irrevocable, royalty-free basis to BMJ Publishing Group Ltd ("BMJ") its licensees and where the relevant Journal is co-owned by BMJ to the co-owners of the Journal, to publish the Work in this journal and any other BMJ products and to exploit all rights, as set out in our licence.

The Submitting Author accepts and understands that any supply made under these terms is made by BMJ to the Submitting Author unless you are acting as an employee on behalf of your employer or a postgraduate student of an affiliated institution which is paying any applicable article publishing charge ("APC") for Open Access articles. Where the Submitting Author wishes to make the Work available on an Open Access basis (and intends to pay the relevant APC), the terms of reuse of such Open Access shall be governed by a Creative Commons licence – details of these licences and which Creative Commons licence will apply to this Work are set out in our licence referred to above.

Other than as permitted in any relevant BMJ Author's Self Archiving Policies, I confirm this Work has not been accepted for publication elsewhere, is not being considered for publication elsewhere and does not duplicate material already published. I confirm all authors consent to publication of this Work and authorise the granting of this licence.

For peer review only

A national survey of attitudes towards and intentions to vaccinate against COVID-19: implications for communications

Martine Stead, Deputy Director[1*]
Curtis Jessop, Research Director for Longitudinal Surveys[2]
Kathryn Angus, Research Officer[1]
Helen Bedford, Professor of Children's Health[3]
Michael Ussher, Professor of Behavioural Medicine[1,4]
Allison Ford, Research Fellow[1]
Douglas Eadie, Research Fellow[1]
Andy MacGregor, Director of Policy Research[2]
Kate Hunt, Professor of Behavioural Sciences and Health[1]
Anne Marie MacKintosh, Senior Researcher[1]

[1]Institute for Social Marketing and Health, University of Stirling, Stirling, UK
[2]NatCen The National Centre for Social Research, London, UK
[3]Great Ormond Street Institute of Child Health, University College London, London, UK
[4]Population Health Research Institute, St George's University of London, London, UK

*Corresponding author: martine.stead@stir.ac.uk, +44(0)1786 467390, Institute for Social Marketing and Health, University of Stirling, Stirling, FK9 4LA, UK

For peer review only - http://bmjopen.bmj.com/site/about/guidelines.xhtml

Abstract

Objectives

To examine public views on COVID-19 vaccination and consider the implications for communications and targeted support.

Design

Cross-sectional study.

Setting

Online and telephone nationally representative survey in Great Britain, January to February 2021.

Participants

4,978 adults. Survey response rate was 84%, among the 5,931 panellists invited.

Main Outcome Measures

Sociodemographic characteristics (age, gender, ethnicity, education, financial status), COVID-19 status, vaccine acceptance, trust in COVID-19 vaccination information sources, perceptions of vaccination priority groups, and perceptions of importance of second dose.

Results

COVID-19 vaccine acceptance (83%) was associated with increasing age, higher level of education and having been invited for vaccination. Acceptance decreased with unconfirmed past COVID-19, greater financial hardship, and non-White British ethnicity; Black/Black British participants had lowest acceptance. Overall, healthcare and scientific sources of information were most trusted. Compared with White British participants, other ethnicities had lower trust in healthcare and scientific sources. Those with lower educational attainment or financial hardship had lower trust in healthcare and scientific sources. Those with no qualifications had higher trust in media and family/friends. While trust was low overall in community or faith leaders it was higher among those with Asian/Asian British and Black/Black British ethnicity compared with White British participants. Views of vaccine prioritisation were mostly consistent with UK official policy but there was support for prioritising additional groups. There was high support for having the second vaccine dose.

Conclusions

Targeted engagement is needed to address COVID-19 vaccine hesitancy in non-White British ethnic groups, in younger adults, and among those with lower education, greater financial hardship and unconfirmed past infection. Healthcare professionals and scientific advisors should play a central role in communications and tailored messaging is needed for hesitant groups. Careful communication around vaccination prioritisation continues to be required.

For peer review only

For peer review only - http://bmjopen.bmj.com/site/about/guidelines.xhtml

STRENGTHS AND LIMITATIONS OF THIS STUDY

- The survey was conducted at the start of vaccine rollout giving timely insight into COVID-19 vaccine acceptance/hesitancy and trusted information sources when individuals' decision-making was real rather than hypothetical.
- Results come from a large probability-based sample, representative of adults in Great Britain, which was sufficiently large to examine ethnicity in detail.
- The survey measured acceptance of which groups should be prioritised for vaccination and the importance of a second dose.
- The survey benefited from a rigorous design, with questionnaire development informed by cognitive interviews conducted with a broad range of individuals.
- A cross-sectional survey cannot infer causality; although variables likely to be important in vaccine acceptance were included, the results are exploratory.

For peer review only

For peer review only - http://bmjopen.bmj.com/site/about/guidelines.xhtml

Introduction

Widespread vaccination is likely to be one of the most effective ways of controlling the COVID-19 pandemic, and is central to the UK government's recovery strategy. The UK vaccine programme began in December 2020, prioritising older adults in care homes and their carers, those aged over 80, and frontline health and social-care workers.[1] Administration of first doses of vaccination to the adult population, by decade of age, is to be completed by July 2021. Uncertainty or unwillingness to accept vaccination – 'vaccine hesitancy'[2] – threatens comprehensive vaccination.[3,4] Before the introduction of a COVID-19 vaccine, UK surveys reported that 64% to 82% of adults were willing to be vaccinated.[5-12] Most of these studies used non-probability samples, introducing selection bias and limiting generalisability. Increased vaccine confidence has been reported since vaccination commenced;[13] possibly due to increased COVID-19 cases and deaths, a further UK lockdown in early 2021, and, increasingly, vaccination becoming the social norm. It is important to examine vaccine acceptance when people are making *active,* rather than *hypothetical,* decisions about vaccination. This also provides insight into potential acceptance of repeat COVID-19 vaccination and boosters.[14]

UK uptake has been high (94% of adults surveyed in April reported uptake or intention to accept vaccination),[13] but there remain concerns about uptake in subpopulations, such as younger adults and some ethnic minorities,[15] giving rise to initiatives such as social media campaigns featuring non-White celebrities.[16] Robust, timely data are needed to identify the characteristics of groups with lower acceptance and the information sources they trust, to inform targeted interventions. It is also important to assess whether attitudes towards COVID-19 vaccination have been affected by specific events and media coverage. Two issues in the UK merit particular attention. First, the government followed recommendations to offer the vaccine to priority groups.[1] If this approach is continued, it is important to examine its acceptability and any implications for communications. Secondly, the government decided, on 30th December 2020, to deviate from recommended protocols for the Pfizer-BioNTech vaccine by extending the interval between doses to up to 12 weeks;[1] this precipitated concerns that it may lead to reduced willingness to be vaccinated or to have a second dose.[17]

We conducted a survey in early 2021, using probability sampling, to examine public views on COVID-19 vaccination and consider the implications for communications. During this period most people aged over 80 had been invited to have a vaccine and invitations were being extended to those aged over 70, with other age groups advised they would be invited in the coming months.

Methods

For peer review only - http://bmjopen.bmj.com/site/about/guidelines.xhtml

We administered a cross-sectional survey with adults (aged 18+) in Great Britain (GB) in January and February 2021. This paper follows the STROBE Statement for reporting cross-sectional studies (see checklist in Supplementary Material).

Questionnaire development and testing

The questionnaire was informed by a review of studies on public attitudes towards and experiences of vaccines and COVID-19. Existing measures were adapted[5,18,19] and new questions developed. The questionnaire was cognitively tested to ensure understandability.[20] Interviews were conducted with 20 individuals with a mix of genders, ages, parental status, likelihood of accepting a COVID-19 vaccination, and experiences of shielding. The questionnaire was subsequently revised based on these interviews. Final revisions reflected changes in the UK's vaccine rollout. The questionnaire covered: vaccine acceptance, trust in vaccine information sources, perception of priority groups, COVID-19 status, and perceived importance of a second dose.

Sample and data collection

The survey was administered to the probability-based NatCen Panel,[21] recruited from the 2018, 2019, and 2020 waves of the British Social Attitudes survey, with participants randomly selected from England, Wales and Scotland. Data were collected through online and telephone interviews (conducted January 14th to February 7th 2021). Panellists were sent reminders and offered a small financial sum in recognition of their contribution. Among 5,931 panellists invited, the survey response rate was 84%, with 4,978 completing it (4,776 online, 202 by telephone). Supplementary Material, Table S1 details overall response rate, accounting for non-response at the panel recruitment stage and panel attrition. Data were weighted for non-response and to be representative of the GB adult population.

Measures

Sociodemographic and other characteristics

Data on age, gender, ethnicity, education, country, urban/rural status, and financial status were obtained from existing information on NatCen panellists. Full details of sub-groups of each variable are provided in Tables 1 and 2. Age was categorised into bands from 18-29 years then ten-year bands up to 80+. Self-assigned ethnicity was recorded in six categories, and education in five categories according to highest qualification. As indices of multiple deprivation were not available, self-reported financial status was used. COVID-19 status was derived from two items: 1) *"Have you officially been diagnosed with the coronavirus (COVID-19)?" (yes/no/don't know)*; those answering other than *'yes'* were asked: 2) *"Do you think you have ever had the coronavirus (COVID-19)?"* *(yes-definitely/yes-probably/no-probably not/no-definitely not/don't know)*.

Vaccine measures

Vaccine acceptance was derived from five items: 1) "*Have you been offered a vaccine for COVID-19?*" (*yes/no*). Those answering '*yes*' were asked: 2) "*And have you had that vaccine?*" (*yes/no*). Participants who had been offered but not yet had the vaccine were then asked: 3) "*And do you*

For peer review only - http://bmjopen.bmj.com/site/about/guidelines.xhtml

intend to have that vaccine?" (*yes/no/not sure*). Participants who had not yet been offered the vaccine were asked: 4) "*Would you accept the vaccine for yourself if it is offered to you?*" (*yes/no/not sure*). Those answering '*not sure*' were asked: 5) "*If you had to choose, if a COVID-19 vaccine became publicly available and you were offered it, would you accept the vaccine for yourself?*" (*yes/no/I'm really not sure*). Participants were classed as: 'Accepted/accepting' if they answered '*yes*' to any of items 2, 3, 4, or 5; 'Uncertain' if they answered '*not sure*' to item 3 or '*I'm really not sure*' to item 5; and 'Refused/refusing' if they answered '*no*' to items 3, 4, or 5.

Trust in information sources was assessed for 13 sources: "*To what extent, if at all, would you trust information about a COVID-19 vaccine from each of the following sources?*" (see Table 3): *completely* (1); *a great deal* (2); *somewhat* (3); *very little* (4); *not at all* (5).

Perceptions of vaccine priority groups were assessed across 11 groups (see Table 4): "*Below are some groups that some people say should be the first to be offered a COVID-19 vaccine. For each one, how high a priority do you think it is that they get a COVID-19 vaccine, or do you not think they should be offered the vaccine at all?*": 1 '*One of the first*', 5 '*One of the last*', with an additional option "*They should not be offered a vaccine*".

Perceived importance of receiving the second dose of the vaccine was assessed with: "*How important, if at all, do you think it is for people to get the second injection of the COVID-19 vaccine?*": *very important* (1); *fairly important* (2); *not very important* (3); *not at all important* (4).

Data analysis

Descriptive data, including bivariate analyses, were weighted to be representative of British adult population. Initial bivariate analyses, using chi-square tests, examined correlates of vaccine acceptance and trust in sources of information about COVID-19 vaccination. Multivariate logistic regression was conducted to examine differences in vaccine acceptance controlling for socio-demographic variables, vaccine offer, and COVID-19 status. The dependent variable dichotomised those classed as *accepted/intending to accept* vs *uncertain/refused/intend to refuse*. Age was entered as a categorical variable and the 'difference' contrast within SPSS logistic regression was used to test influence of each increasing age group, relative to younger ages (e.g., 30-39 vs 18-29; 80+ vs 18-79) (see Table 2). Sociodemographic variation in trust in information sources was examined using multivariate logistic regressions. For each information source, the dependent variable dichotomised the 5-point scale into trusting *completely or a great deal* vs *somewhat/very little/not at all*. Cases were excluded from the logistic regressions if they had missing data on the dependent or any independent variables. All logistic regressions were conducted on unweighted data as sociodemographic variables were included as control variables. For each information source, logistic regression analysis examined likelihood of trust (completely/a great deal v somewhat/very little/not at all) by sociodemographic characteristics (Supplementary Material, Tables S2-S14). Data were analysed using SPSS v27.

For peer review only - http://bmjopen.bmj.com/site/about/guidelines.xhtml

Results

Sample characteristics

The weighted sample comprised adults aged 18 and over (see Table 1). Over half (52%) were female and 81% were White British. Around two-thirds reported 'living comfortably'/'doing alright', while one in ten rated their financial status as 'quite' or 'very difficult'. Just over two-fifths were educated to degree level or above, while for almost a quarter their highest qualification was A level or equivalent. A minority (12%) had no qualifications. A minority indicated having been diagnosed with COVID-19 (6%); nearly two-thirds thought they probably or definitely had not had COVID-19; 11% were unsure.

Vaccine offer and acceptance

At the time of the survey, 14% (n=716) had been offered the vaccine. Of these, 92% (n=658) had accepted or intended to, 4% (n=29) were uncertain, and 4% (n=29) had refused or intended to refuse.

Among those not yet offered the vaccine, 82% (n=3479) intended to accept, while 11% (n=471) were uncertain and 7% (n=311) indicated they would refuse. Overall, the acceptance level was 83% (n=4137), with 10% (n=502) uncertain and 7% (n=340) refusing.

Multivariate logistic regression, with vaccine acceptance as the outcome variable (accepted/accepting v refused/refusing/uncertain), indicated likelihood of acceptance increased with age (Table 2). For example, those aged 40-49 were more likely than 18-39-year-olds to indicate acceptance (AOR=1.43, 95%CI (1.12, 1.83, p=0.004) as were 70-79-year-olds compared with 18-69-year-olds (AOR=3.31, 95%CI (2.22, 4.95), p<0.001). Acceptance was also positively associated with education. Those with at least a degree were three times as likely to indicate acceptance (AOR=3.03, 95%CI (2.17, 4.23), p<0.001) and those educated to A level or equivalent nearly twice as likely (AOR=1.80, 95%CI (1.27, 2.55), p<0.001), compared with people without qualifications. Lower acceptance was also associated with financial hardship and ethnicity. For example, compared with those 'living comfortably', people 'finding it very difficult' were much less likely to accept the vaccine (AOR=0.35, 95%CI (0.22, 0.55), p<0.001). Compared with White British participants, those from other ethnic groups were less likely to accept the vaccine. Black/Black British participants had the lowest likelihood of accepting (AOR=0.25, 95%CI (0.14, 0.43), p<0.001). This is illustrated in the descriptive data too, with 87% of White British participants indicating vaccine acceptance compared with 58% among Black/Black British, 61% among mixed/multiple ethnic groups and 61% among Asian/Asian British.

After controlling for demographic variables, vaccine acceptance was positively associated with having been invited for vaccination (AOR=1.73, 95%CI (1.24, 2.43), p=0.001), but negatively associated with COVID-19 status. Compared with those who had 'probably not' or 'definitely not' had COVID-19, those who thought they had 'definitely' or 'probably' had COVID-19 were less likely

For peer review only - http://bmjopen.bmj.com/site/about/guidelines.xhtml

to indicate acceptance (AOR = 0.40, 95%CI (0.26, 0.60), p<0.001 and AOR=0.71, 95%CI (0.56, 0.91), p=0.006 respectively). Confirmed diagnosis with COVID-19 was not significantly associated with vaccine acceptance, after controlling for demographic variables.

Trust in information sources

The three most trusted information sources were: the NHS; doctors/nurses/other healthcare professionals; and scientific and medical advisers. These groups were trusted ‘completely/a great deal’ by around 80% of participants (Table 3). Only 44% trusted the UK government ‘completely/a great deal’. The three least trusted sources were celebrities and social media influencers, social media, and faith or community leaders; around two-thirds indicated they would have no trust in each. A majority (61%) indicated they had very little/no trust in the media (e.g., newspapers/magazines/television/radio).

Trust did not differ by gender except for drug companies and the WHO, with females more likely to indicate trust in these sources (Tables S5 and S9 respectively).

Trust was higher among older participants for five sources (doctors/nurses/other healthcare professionals, NHS, UK government, media, and family/friends; Tables S2, S4, S6, S10, S13). For example, trust in the UK government was higher among those aged 50-59 than 18-49-year-olds (Table S6).

Trust varied by education. Compared with those without qualifications, other participants were more likely to trust five sources (doctors/nurses/other healthcare professionals, pharmacists, NHS, scientists, WHO; Tables S2-S4, S8, S9) and less likely to trust another five (drug companies, media, social media, celebrities/social media influencers, family/friends; Tables S5, S10-S13). Compared with those ‘living comfortably’ participants in more difficult financial situations were less likely to trust the seven sources most closely aligned with scientific or clinical expertise (doctors/nurses/other healthcare professionals, pharmacists, NHS, drug companies, UK government, scientists, WHO; Tables S2-S6, S8, S9). Similarly, participants from minority ethnic groups were less likely to trust scientific or clinical sources than White British participants (Tables S2-S4, S8, S9). Whilst lack of trust in faith or community leaders was low overall, Asian/Asian British participants were more likely than White British to trust faith/community leaders (AOR=4.82, 95%CI (2.76, 8.42), p<0.001) as were Black/Black British participants (AOR=4.52, 95%CI (2.04, 9.99), p<0.001).

Views on prioritisation

Nine in ten participants rated healthcare professionals as highest priority for vaccination. Over 70% indicated those with serious health conditions/heightened vulnerability to COVID-19, care home workers and residents, and over 80s should be ‘one of the first’ to be vaccinated (Table 4). Priority was also given to social care workers, schoolteachers, and those directly working with the public. Over a third considered each of these groups should be ‘one of the first’ to be vaccinated, and 70% or more rated them in the top two priority levels. People aged under 18 were rated as lowest priority, and 6% considered the vaccine should not be offered to this group.

For peer review only - http://bmjopen.bmj.com/site/about/guidelines.xhtml

Importance of second dose

Nearly all participants (96%, n=4,761) considered it 'very' or 'fairly important' to receive the second vaccine dose. This increased to 99% (n=4,096) amongst those who intended to accept the vaccine.

Discussion

Principal findings

Overall, acceptance was high, with 83% having received or intending to have the vaccine. While this suggests acceptance will be high in future vaccination programmes, it may change if perceived vulnerability to or severity of infection, vaccine efficacy, or side-effects alters. Acceptance increased with age and education, and if invited for vaccination. It decreased with financial hardship, and among non-White British ethnicities and those with unconfirmed past COVID-19. Clinical and scientific information was most trusted, with sociodemographic differences for different sources. Policy on a second dose and vaccination priority groups[1] was supported.

Comparison with other studies

We confirmed lower acceptance in younger groups;[6-8,10,11] acceptance was higher if invited for vaccination, a finding observed for other vaccines in other populations ,[22] and emphasising the importance of ensuring vaccine invitations are issued, using appropriate language with translations if necessary. Confirmation of lower acceptance in non-White British ethnicities.[5,6,9,23] is concerning given increased risk of infection and poorer outcomes.[24] We confirmed lower acceptance in those with lower educational attainment and greater financial hardship,[6,8-10,12,25] leaving these groups at risk of infection and increasing likelihood of emergence of variants.[26]

Those with unconfirmed but suspected COVID-19 had lower acceptance. This suggests infection is thought to confer immunity, or recovery fosters a perception of decreased severity. However, past infection does not guarantee protection and people may still be infectious.[27,28] Messaging should target those with prior infection.

There are other implications for communications. While high acceptance suggests communications are effective, identifying barriers in hesitant groups is a priority for developing interventions.[3,15,18,29] Trusted information sources are needed. The most trusted were the NHS, healthcare professionals, and scientific and medical advisers. This suggests that healthcare professionals have a central role in promoting vaccination in initiatives and during consultations. That government and media are less trusted has implications for acceptance.[7,8,25,30] Without sophisticated tailoring and evaluation, social media and celebrities may fail to promote vaccination; initiatives using ethnic minority celebrities and opinion leaders show promise.[16]

Differences in trust varied by socio-demographics. Compared with White British participants, other ethnicities had lower trust in healthcare and scientific sources. Although trust in faith/community leaders was low, it was higher in Asian and Black British participants, suggesting a role for these

For peer review only - http://bmjopen.bmj.com/site/about/guidelines.xhtml

leaders.[15] Those with lower educational attainment or financial hardship had lower trust in healthcare and scientific sources. Those with no qualifications had higher trust in media and family/friends. This suggests a need for a mix of sources for these groups. Mainstream media may have a role to play, despite lower trust.[25]

Reassuringly for further campaigns, prioritisation was considered acceptable and there was support for additional prioritisation of schoolteachers and others in direct contact with the public. As planning begins for further vaccination, careful communication regarding prioritisation should continue. We found high support for a second dose, suggesting the UK's decision to extend the period between doses has not dented public confidence.

Strengths and limitations

Strengths include the large probability-based nationally representative sample, ability to analyse by ethnicity and surveying during vaccine roll-out. Our findings can be generalised to GB's adult population, however global contexts for COVID-19 and vaccination vary. Although not generalisable to them, the findings are still informative for other countries. The study has limitations. As it is cross-sectional, we cannot infer causality; although we included variables likely to be important in vaccine acceptance, these results are exploratory. Our qualitative studies will deepen understanding of associations. A survey repeated when COVID-19 cases and deaths are low, and without lockdown, might yield different responses. We did not survey individuals who are institutionalised (e.g., prisoners), notably difficult to reach (e.g., homeless), or those not speaking English (therefore, our ethnic minority sample may underrepresent certain views); specific surveys are needed for these groups. We investigated vaccination intention. Actual uptake may be lower, although it is likely that factors associated with intention will influence uptake.

Conclusions

COVID-19 vaccination acceptance is high in GB. Targeted engagement is needed to address hesitancy in non-White British ethnic groups, those with lower education, those younger, those with greater financial hardship and those with unconfirmed but suspected past infection. Healthcare professionals and scientific advisors should lead communications and tailoring is needed. Work is needed to rebuild trust in government information. There is high support for having the second vaccine dose. Views of vaccine prioritisation are mostly consistent with UK official policy but there was support for prioritising additional groups and careful communication around vaccination prioritisation should continue.

For peer review only - http://bmjopen.bmj.com/site/about/guidelines.xhtml

Contributors:

MS, CJ, HEB, KH, and AMM conceived the study, supported by AF, DE, and AM. MS, CJ, KA, HB, and AMM designed the questionnaire, supported by MU, AF, DE, AM, and KH. CJ and AMM acquired and analysed the data, which was interpreted by MS, CJ, HB, MU, KH and AMM. MS and AMM drafted the manuscript supported by CJ, HB, MU, and KH. KA, HB, MU, and KH critically revised the article, supported by MS, CJ, AF, DE, AM, and AMM. All authors read the final version of the manuscript and gave approval for it to be published. AMM, CJ, and MS had access to the data in the study and take responsibility for the integrity of the data and the accuracy of the data analysis. The corresponding author attests that all listed authors meet authorship criteria and that no others meeting the criteria have been omitted. MS is the guarantor.

Public and Patient Involvement:

The questionnaire was cognitively tested by members of the public to ensure understandability.

Ethics approval:

The study received ethical approval from NatCen's Research Ethics Committee (ID P14307). Participants gave informed consent before taking part.

Transparency declaration:

The lead author and manuscript's guarantor (MS) affirms that the manuscript is an honest, accurate, and transparent account of the study being reported; that no important aspects of the study have been omitted; and that any discrepancies from the study as originally planned have been explained.

Role of the funding source:

The OPTIMising general public Uptake of a COVID-19 vaccine (OPTIMUM) study was supported by a UK Research & Innovation (UKRI) Ideas to Address COVID-19 award (no. ES/V012851/1). The funder had no role in the study design; in the collection, analysis, and interpretation of data; in the writing of the report; and in the decision to submit the article for publication. All researchers were independent from the funder and all authors had full access to all of the data (including statistical reports and tables) in the study and can take responsibility for the integrity of the data and the accuracy of the data analysis.

Dissemination to participants and related patient and public communities:

We do not plan to disseminate study results to individual study participants, however findings from the OPTIMUM study will be shared with the public via press releases, social media and interviews, and lay-audience briefing papers.

Data sharing:

After completion of the study, the survey dataset will be deposited in the UK Data Archive.

Competing interests:

All authors have completed the ICMJE uniform disclosure form at www.icmje.org/coi_disclosure.pdf and declare: no support from any organisation for the submitted work; KH has received another UK

For peer review only - http://bmjopen.bmj.com/site/about/guidelines.xhtml

Research and Innovation (Economic and Social Research Council) grant on the impact of COVID-19; no other relationships or activities that could appear to have influenced the submitted work.

Acknowledgments:

We thank the questionnaire development and testing and survey and data delivery teams at NatCen for their work on the survey, and Professor Mark Petticrew at the London School of Hygiene & Tropical Medicine for acting as adviser to the study.

For peer review only

For peer review only - http://bmjopen.bmj.com/site/about/guidelines.xhtml

References

1 UK Government Department of Health and Social Care. Joint Committee on Vaccination and Immunisation: advice on priority groups for COVID-19 vaccination, 30 December 2020. Jan 6, 2021. https://www.gov.uk/government/publications/priority-groups-for-coronavirus-covid-19-vaccination-advice-from-the-jcvi-30-december-2020/joint-committee-on-vaccination-and-immunisation-advice-on-priority-groups-for-covid-19-vaccination-30-december-2020.

2 MacDonald NE, SAGE Working Group on Vaccine Hesitancy. Vaccine hesitancy: definition, scope and determinants. *Vaccine* 2015;33:4161-4.

3 Larson HJ, Jarrett C, Eckersberger E, Smith DMD, Paterson P. Understanding vaccine hesitancy around vaccines and vaccination from a global perspective: a systematic review of published literature, 2007-2012. *Vaccine* 2014;32:2150-9.

4 Lane S, MacDonald NE, Marti M, Dumolard L. Vaccine hesitancy around the globe: analysis of three years of WHO/UNICEF Joint Reporting Form data—2015–2017. *Vaccine* 2018;36:3861-7.

5 Bell S, Clarke R, Mounier-Jack S, Walker JL, Paterson P. Parents' and guardians' views on the acceptability of a future COVID-19 vaccine: A multi-methods study in England. *Vaccine* 2020;38:7789-98.

6 Freeman D, Loe BS, Chadwick A, Vaccari C, Waite F, Rosebrock L, et al. COVID-19 vaccine hesitancy in the UK: the Oxford coronavirus explanations, attitudes, and narratives survey (Oceans) II. *Psychol Med* 2020; published online Dec 11. https://dx.doi.org/10.1017/S0033291720005188.

7 Lazarus JV, Ratzan SC, Palayew A, Gostin LO, Larson HJ, Rabin K, et al. Extended data fig. 1: 'If a COVID-19 vaccine is proven safe and effective and is available, I will take it'. In: A global survey of potential acceptance of a COVID-19 vaccine. *Nat Med* 2021;27:225-8. https://doi.org/10.1038/s41591-020-1124-9.

8 Murphy J, Vallières F, Bentall RP, Shevlin M, McBride O, Hartman TK, et al. Psychological characteristics associated with COVID-19 vaccine hesitancy and resistance in Ireland and the United Kingdom. *Nat Commun* 2021;12:29.

9 Robertson E, Reeve KS, Niedzwiedz CL, Moore J, Blake M, Green M, et al. Predictors of COVID-19 vaccine hesitancy in the UK household longitudinal study. *Brain Behav Immun* 2021;94:41-50.

10 Paul E, Steptoe A, Fancourt D. Attitudes towards vaccines and intention to vaccinate against COVID-19: Implications for public health communications. *Lancet Reg Health Eur* 2021;1:100012. https://doi.org/10.1016/j.lanepe.2020.100012

11 Sherman SM, Smith LE, Sim J, Amlôt R, Cutts M, Dasch H, et al. COVID-19 vaccination intention in the UK: results from the COVID-19 Vaccination Acceptability Study (CoVAccS), a nationally representative cross-sectional survey. *Hum Vaccin Immunother* 2020;26:1-10.

For peer review only - http://bmjopen.bmj.com/site/about/guidelines.xhtml

12 Williams L, Flowers P, McLeod J, Young D, Rollins L. The Catalyst Project Team. Social patterning and stability of intention to accept a COVID-19 vaccine in Scotland: will those most at risk accept a vaccine? *Vaccines (Basel)* 2021;9:17.

13 Office for National Statistics. Coronavirus (COVID-19) latest insights. Apr 16, 2021. https://www.ons.gov.uk/peoplepopulationandcommunity/healthandsocialcare/conditionsanddiseases/articles/coronaviruscovid19/latestinsights#vaccinations.

14 Mahase E. Covid-19: Booster vaccine to be rolled out in autumn as UK secures 60m more Pfizer doses. *BMJ* 2021;373:n1116.

15 Razai MS, Chaudhry UAR, Doerholt K, Bauld L, Majeed A. Covid-19 vaccination hesitancy. *BMJ* 2021;373:n1138.

16 Mohdin A. BAME groups urged to have Covid vaccine in UK TV ad campaign. *The Guardian*, Feb 18, 2021. https://www.theguardian.com/world/2021/feb/18/bame-groups-urged-to-have-covid-vaccine-in-uk-tv-ad-campaign.

17 Sewell HF, Robertson JFR, Stewart M, Kendrick D, Bird SM. Revisiting the UK's strategy for delaying the second dose of the Pfizer covid-19 vaccine. Jan 20, 2021. https://blogs.bmj.com/bmj/2021/01/20/revisiting-the-uks-strategy-for-delaying-the-second-dose-of-the-pfizer-covid-19-vaccine/.

18 World Health Organization Regional Office for Europe. Survey Tool and Guidance. Rapid, simple, flexible behavioural insights on COVID-19. Copenhagen: WHO Regional Office for Europe; 2020. https://www.euro.who.int/__data/assets/pdf_file/0007/436705/COVID-19-survey-tool-and-guidance.pdf?ua=1.

19 Gallup. Ch 5 Attitudes to vaccines. In: Gallup. Wellcome Global Monitor. How does the world feel about science and health? London: Wellcome Trust, 2019: 104-25. https://cms.wellcome.org/sites/default/files/wellcome-global-monitor-2018.pdf.

20 Collins D. Cognitive Interviewing Practice. London: SAGE Publications Ltd, 2014.

21 Jessop C. The NatCen Panel: developing an open probability-based mixed-mode panel in Great Britain. *Soc Res Pract* 2018;6:2-14. https://the-sra.org.uk/Common/Uploaded%20files/Social%20Research%20Practice%20Journal/social-research-practice-journal-issue-06-summer-2018.pdf.

22 Jacobson Vann JC, Jacobson RM, Coyne-Beasley T, Asafu-Adjei JK, Szilagyi PG. Patient reminder and recall interventions to improve immunization rates. *Cochrane Database Syst Rev* 2018;1:CD003941.

23 Thorneloe R, Wilcockson H, Lamb M, Jordan CH, Arden M. Willingness to receive a COVID-19 vaccine among adults at high-risk of COVID-19: a UK-wide survey. *PsyArXiv* 2020; published online Jul 20. https://psyarxiv.com/fs9wk (preprint).

24 Mathur R, Rentsch CT, Morton CE, Hulme WJ, Schultze A, MacKenna B, et al. Ethnic differences in SARS-CoV-2 infection and COVID-19-related hospitalisation, intensive care unit

For peer review only - http://bmjopen.bmj.com/site/about/guidelines.xhtml

For peer review only

admission, and death in 17 million adults in England: an observational cohort study using the OpenSAFELY platform. *Lancet*. 2021;397:1711-24.

25 Allington D, McAndrew S, Moxham-Hall V, Duffy B. Coronavirus conspiracy suspicions, general vaccine attitudes, trust and coronavirus information source as predictors of vaccine hesitancy among UK residents during the COVID-19 pandemic. *Psychol Med* 2021; published online April 12. https://doi.org/10.1017/s0033291721001434

26 Sah P, Vilches TN, Moghadas SM, Fitzpatrick MC, Singer BH, Hotez PJ, et al. Accelerated vaccine rollout is imperative to mitigate highly transmissible COVID-19 variants. *EClinicalMedicine* 2021;35:100865.

27 Hansen CH, Michlmayr D, Gubbels SM, Mølbak K, Ethelberg S. Assessment of protection against reinfection with SARS-CoV-2 among 4 million PCR-tested individuals in Denmark in 2020: a population-level observational study. *Lancet* 2021;397:1204-12.

28 Hall VJ, Foulkes S, Charlett A, Atti A, Monk EJM, Simmons R, et al. SARS-CoV-2 infection rates of antibody-positive compared with antibody-negative health-care workers in England: a large, multicentre, prospective cohort study (SIREN). *Lancet* 2021;397:1459-69.

29 Lockyer B, Islam S, Rahman A, Dickerson J, Pickett K, Sheldon T, et al. Understanding COVID-19 misinformation and vaccine hesitancy in context: Findings from a qualitative study involving citizens in Bradford, UK. *Health Expect* 2021; published online May 4. https://dx.doi.org/10.1111/hex.13240

30 Williams L, Gallant AJ, Rasmussen S, Brown Nicholls LA, Cogan N, et al. Towards intervention development to increase the uptake of COVID-19 vaccination among those at high risk: Outlining evidence-based and theoretically informed future intervention content. *Br J Health Psychol* 2020;25:1039-54.

For peer review only

For peer review only - http://bmjopen.bmj.com/site/about/guidelines.xhtml

Table 1 Sample characteristics

	Unweighted		Weighted	
	n	%	n	%
**Age**				
18-29	464	9.4%	824	16.7%
30-39	772	15.6%	852	17.3%
40-49	848	17.1%	806	16.3%
50-59	904	18.3%	867	17.6%
60-69	1011	20.4%	711	14.4%
70-79	773	15.6%	657	13.3%
80+	178	3.6%	218	4.4%
**Gender**				
Male	2136	42.9%	2402	48.3%
Female	2830	56.9%	2567	51.6%
Other	10	0.2%	7	0.1%
**Ethnicity**				
White British	4261	86.3%	3999	81.2%
Any other White background	319	6.5%	335	6.8%
Mixed or multiple ethnic groups	64	1.3%	100	2.0%
Asian or Asian British	164	3.3%	306	6.2%
Black or Black British	67	1.4%	101	2.1%
Other	62	1.3%	81	1.6%
**Country**				
England	4369	87.9%	4291	86.3%
Scotland	390	7.8%	442	8.9%
Wales	212	4.3%	237	4.8%
**Urban/rural status#**				
Urban	3789	76.2%	4006	80.6%
Rural	1182	23.8%	965	19.4%
**Highest educational qualification**				
Degree or equivalent, and above	2503	50.4%	2077	41.8%
A levels or vocational level 3 or equivalent and above, but below degree	1005	20.2%	1131	22.8%
Other qualifications below A levels or vocational level 3 or equivalent	788	15.9%	838	16.9%
Other qualification	256	5.2%	304	6.1%
No qualifications	416	8.4%	618	12.4%
**Subjective Financial Status**				
Living comfortably	1552	31.2%	1289	26.0%
Doing alright	2028	40.8%	2035	40.9%
Just about getting by	975	19.6%	1132	22.8%
Finding it quite difficult	271	5.5%	337	6.8%
Finding it very difficult	142	2.9%	175	3.5%
**COVID-19 Status**				
Diagnosed with COVID-19	241	4.8%	294	5.9%
Think definitely had COVID-19	140	2.8%	172	3.5%
Think probably had COVID-19	710	14.3%	755	15.2%

For peer review only

For peer review only - http://bmjopen.bmj.com/site/about/guidelines.xhtml

Think probably not had COVID-19	1945	39.1%	1880	37.8%
Think definitely not had COVID-19	1393	28.0%	1305	26.2%
Don't know if had COVID-19	547	11.0%	566	11.4%

[#] England and Wales, based on Office for National Statistics (ONS) definition of urban as population greater than 10,000. Scotland based on Scottish Government definition of urban as population greater than 3,000.

For peer review only

For peer review only - http://bmjopen.bmj.com/site/about/guidelines.xhtml

Table 2 Association between vaccine acceptance and sociodemographic variables – (a) bivariate results and (b) multivariate logistic regression.

	**(a) Bivariate associations between vaccine acceptance and socio-demographics** % Accepted/Intend to Accept (weighted) χ^2 test for differences by demographics					**(b) Logistic regression of vaccine acceptance** 1 = Accepted/Intend to Accept (4294), 0 = Uncertain/Refused/Intend to Refuse (600)				
	**n**	**%**		**χ^2 (df)**	**P**	**N**	**AOR***	**95% CI Lower**	**95% CI Upper**	**P**
**Gender**				2.154 (2)	.341					0.085
Male	2012	83.8				2097	ref			
Female	2117	82.5				2788	0.82	0.67	0.99	0.036
Other	5	71.4				9	0.47	0.09	2.45	0.369
**Age**				274.733 (6)	<.001					<.001
18-29	613	74.4				459	ref			
30-39 *v 18-29*	618	72.5				761	0.89	0.66	1.20	.448
40-49 *v 18-39*	640	79.3				835	1.43	1.12	1.83	.004
50-59 *v 18-49*	745	85.9				896	1.92	1.49	2.46	<.001
60-69 *v 18-59*	659	92.7				1003	3.21	2.37	4.34	<.001
70-79 *v 18-69*	629	95.7				763	3.31	2.22	4.95	<.001
80+ *v 18-79*	209	95.9				177	2.19	0.92	5.21	.078
**Education/Highest qualification**				56.056 (4)	<.001					<.001
No qualifications	495	80.1				411	ref			
Degree or equivalent and above	1811	87.2				2454	3.03	2.17	4.23	<.001
A levels / Vocational level 3 or equivalent	909	80.4				990	1.80	1.27	2.55	<.001
Other qual'ns below A level / Voc level 3	694	82.7				784	1.50	1.05	2.15	.026
Other qualification	223	73.4				255	0.90	0.58	1.39	.632
**Financial Status**				168.660 (4)	<.001					<.001
Living comfortably	1162	90.1				1533	ref			
Doing alright	1749	86.0				1998	0.89	0.69	1.15	.383
Just about getting by	848	74.9				959	0.52	0.39	0.69	<.001
Finding it quite difficult	261	77.2				266	0.74	0.50	1.10	.139
Finding it very difficult	111	63.4				138	0.35	0.22	0.55	<.001
**Country**				3.171 (2)	.205					.326
England	3581	83.5				4302	ref			
Scotland	356	80.5				384	0.82	0.59	1.13	.220
Wales	192	81.0				208	0.80	0.51	1.26	.345
**Urban/rural**				34.517 (1)	<.001					
Urban	3266	81.5				3729	ref			
Rural	863	89.4				1165	1.28	1.00	1.65	.051
**Ethnicity**				246.434 (5)	<.001					<.001
White British	3482	87.1				4226	ref			
Any other white background	254	75.8				318	0.55	0.40	0.76	<.001
Mixed or multiple ethnic groups	62	61.4				62	0.39	0.21	0.71	.002
Asian or Asian British	188	61.4				161	0.41	0.28	0.61	<.001
Black or Black British	59	58.4				67	0.25	0.14	0.43	<.001
Other	59	72.8				60	0.42	0.23	0.79	.007
**Whether been offered vaccine**				45.924 (1)	<.001					
No	3479	81.6				4227	ref			
Yes	658	91.9				667	1.73	1.24	2.43	.001
**COVID-19 Status**				72.865 (4)	<.001					<.001

For peer review only - http://bmjopen.bmj.com/site/about/guidelines.xhtml

Think probably or definitely not had COVID-19	2741	86.1				3288	ref			
Diagnosed with COVID-19	218	74.4				240	0.89	0.60	1.33	.575
Think definitely had COVID-19	118	68.2				140	0.40	0.26	0.60	<.001
Think probably had COVID-19	598	79.1				691	0.71	0.56	0.91	.006
Don't Know if had COVID-19	462	81.5				535	0.73	0.55	0.97	.031
						Hosmer & Lemeshow χ^2= 7.444, df=8, p=0.490.<br>Final model χ^2=497.429, df=29, p<0.001<br>Nagelkerke = 0.184<br>Cases correctly classified: 88.1%.<br>84 cases excluded due to missing data on one or more independent variables.				

* adjusted for all other variables in the model, AOR, adjusted odds ratio; ref, reference category; 95%CI, 95% confidence interval

For peer review only

For peer review only - http://bmjopen.bmj.com/site/about/guidelines.xhtml

Table 3 Trust in potential sources of information on COVID-19 vaccine

Source:	Level of Trust (trust completely [1]…not at all [5]) Completely (1) n	%	A great deal (2) n	%	Somewhat (3) n	%	Very little (4) n	%	Not at all (5) n	%	Mean	Std Dev
The NHS	2084	41.9%	1902	38.3%	701	14.1%	155	3.1%	127	2.5%	1.86	0.95
Doctors, nurses or other healthcare professionals	1918	38.6%	2092	42.1%	714	14.4%	154	3.1%	90	1.8%	1.87	0.90
Scientific and medical advisers	1798	36.2%	2101	42.3%	792	15.9%	160	3.2%	121	2.4%	1.94	0.93
The World Health Organisation (WHO)	1313	26.4%	2016	40.6%	1070	21.6%	310	6.2%	256	5.1%	2.23	1.07
Pharmacists	999	20.1%	1973	39.7%	1434	28.8%	341	6.9%	226	4.5%	2.36	1.02
The UK Government	654	13.2%	1542	31.1%	1739	35.1%	614	12.4%	402	8.1%	2.71	1.10
Scottish Govt/Welsh Assembly[a]	118	17.4%	189	27.9%	207	30.5%	88	13.1%	75	11.1%	2.72	1.21
Drug companies who manufacture vaccines	406	8.2%	1064	21.4%	2065	41.6%	771	15.5%	661	13.3%	3.04	1.11
Family and friends	343	6.9%	876	17.6%	2230	44.9%	977	19.7%	542	10.9%	3.10	1.04
The media (e.g. newspapers, magazines, television, radio)	86	1.7%	302	6.1%	1567	31.5%	1433	28.9%	1580	31.8%	3.83	1.00
Faith or community leaders	131	2.6%	124	2.5%	619	12.5%	827	16.7%	3264	65.7%	4.40	0.98
Social media (e.g. Twitter, Facebook, Instagram etc)	65	1.3%	69	1.4%	506	10.2%	1267	25.5%	3056	61.6%	4.45	0.83
Celebrities and social media influencers	60	1.2%	71	1.4%	493	9.9%	1175	23.6%	3170	63.8%	4.47	0.82

Base: All participants (weighted). Missing cases range from n=3 to n=27. [a]Base: all participants in Scotland or Wales, n=679 (weighted). List order was randomised for each participant.

For peer review only - http://bmjopen.bmj.com/site/about/guidelines.xhtml

Table 4 Views on priority groups for vaccination: who should be first and last groups vaccinated

	Should not be offered		Priority of being offered[a]										Mean[b]	Std Dev
			One of the first (1)		(2)		(3)		(4)		One of the last (5)			
	n	%	n	%	n	%	n	%	n	%	n	%		
Doctors, nurses and other healthcare professionals	33	0.7%	4472	90.0%	280	5.6%	83	1.7%	15	0.3%	83	1.7%	1.17	0.63
People with serious health conditions which mean they are vulnerable to COVID-19	35	0.7%	4017	80.9%	671	13.5%	129	2.6%	35	0.7%	77	1.6%	1.27	0.69
Care home workers	36	0.7%	3926	79.0%	683	13.8%	197	4.0%	58	1.2%	66	1.3%	1.31	0.72
Residents in a care home	47	0.9%	3593	72.4%	734	14.8%	337	6.8%	123	2.5%	131	2.6%	1.47	0.93
People aged 80 or over	49	1.0%	3613	72.9%	706	14.2%	304	6.1%	118	2.4%	168	3.4%	1.48	0.96
Social care workers	33	0.7%	2683	54.0%	1348	27.2%	683	13.8%	143	2.9%	75	1.5%	1.70	0.92
Schoolteachers	47	0.9%	2098	42.2%	1621	32.6%	886	17.8%	223	4.5%	94	1.9%	1.90	0.97
People with jobs that involve direct contact with members of the public	45	0.9%	1864	37.5%	1603	32.3%	1157	23.3%	228	4.6%	70	1.4%	1.99	0.96
People aged 31-50	43	0.9%	154	3.1%	614	12.4%	2096	42.2%	1486	30.0%	568	11.4%	3.35	0.95
People aged 18-30	102	2.0%	123	2.5%	289	5.8%	943	19.0%	1375	27.7%	2130	42.9%	4.05	1.05
People aged under 18	282	5.7%	148	3.0%	253	5.1%	657	13.3%	831	16.8%	2788	56.2%	4.25	1.08

Base: All participants (weighted). [a] Missing cases range from n=11 to n=21. [b] Excludes 'should not be offered', missing cases range from n=45 to n=301. List order was randomised for each participant.

For peer review only

Supplementary material to 'A national survey of attitudes towards and intentions to vaccinate against COVID-19: implications for communications'.

Contents

- Table S1 Overall response rate calculation accounting for recruitment onto original panel and panel attrition.
- Table S2 Doctors, nurses or other healthcare professionals – Association between trust in sources of information about COVID-19 vaccine and socio-demographic variables – (a) bivariate results and (b) multivariate logistic regression.
- Table S3 Pharmacists – Association between trust in sources of information about COVID-19 vaccine and socio-demographic variables – (a) bivariate results and (b) multivariate logistic regression.
- Table S4 The NHS – Association between trust in sources of information about COVID-19 vaccine and socio-demographic variables – (a) bivariate results and (b) multivariate logistic regression.
- Table S5 Drug companies who manufacture vaccines – Association between trust in sources of information about COVID-19 vaccine and socio-demographic variables – (a) bivariate results and (b) multivariate logistic regression.
- Table S6 The UK Government – Association between trust in sources of information about COVID-19 vaccine and socio-demographic variables – (a) bivariate results and (b) multivariate logistic regression.
- Table S7 The Scottish/Welsh Government – Association between trust in sources of information about COVID-19 vaccine and socio-demographic variables – (a) bivariate results and (b) multivariate logistic regression.
- Table S8 Scientific and medical advisers – Association between trust in sources of information about COVID-19 vaccine and socio-demographic variables – (a) bivariate results and (b) multivariate logistic regression.
- Table S9 The World Health Organisation (WHO) – Association between trust in sources of information about COVID-19 vaccine and socio-demographic variables – (a) bivariate results and (b) multivariate logistic regression.
- Table S10 The media (e.g. newspapers, magazines, television, radio) – Association between trust in sources of information about COVID-19 vaccine and socio-demographic variables – (a) bivariate results and (b) multivariate logistic regression.
- Table S11 Social media – Association between trust in sources of information about COVID-19 vaccine and socio-demographic variables – (a) bivariate results and (b) multivariate logistic regression.
- Table S12 Celebrities and social media influencers – Association between trust in sources of information about COVID-19 vaccine and socio-demographic variables – (a) bivariate results and (b) multivariate logistic regression.
- Table S13 Family and friends – Association between trust in sources of information about COVID-19 vaccine and socio-demographic variables – (a) bivariate results and (b) multivariate logistic regression.
- Table S14 Faith or community leaders – Association between trust in sources of information about COVID-19 vaccine and socio-demographic variables – (a) bivariate results and (b) multivariate logistic regression.

For peer review only

Table S1 Overall response rate calculation accounting for recruitment onto original panel and panel attrition.

	Wave of British Social Attitudes Survey (BSA) from which panel was recruited			
**Response to initial BSA survey**	**2018**	**2019**	**2020**	**Total 2018 to 2020**
BSA issued	10,270	7,956	42066	60,292
BSA deadwood	1,023	684	4207	5,914
BSA productive	3,879	3,224	3964	11,067
BSA response rate	42%	44%	10%	20%
**Overall response for panel recruitment**				
BSA productive	3,879	3,224	3964	11,067
Recruited to panel	2,412	2,104	3086	7,602
Panel recruitment rate	62%	65%	78%	69%
Panel deadwood	19	7	0	26
Panel lost to attrition/inactivity prior to vaccine survey	969	673	3	1645
**Panel's response to vaccine survey**				
Issued	1,424	1,424	3,083	5,931
Deadwood	1	5	0	6
Achieved	1,242	1,181	2,555	4,978
**Vaccine survey response rate**	**87%**	**83%**	**83%**	**84%**
**Overall survey response rate[a]**	**13%**	**16%**	**7%**	**9%**

[a] Response rate accounting for non-response at original point of recruitment (British Social Attitudes Survey 2018, 2019 or 2020) and panel attrition thereafter.

For peer review only

Table S2 Doctors, nurses or other healthcare professionals – Association between trust in sources of information about COVID-19 vaccine and socio-demographic variables – (a) bivariate results and (b) multivariate logistic regression.

	**(a) Bivariate associations between socio-demographics and trusting COVID-19 vaccine info from Doctors, nurses or other healthcare professionals** % Trust completely or a great deal (weighted) χ^2 test for differences by demographics					**(b) Logistic regression of trust in COVID-19 vaccine info from Doctors, nurses or other healthcare professionals** 1 = Trust completely or a great deal (4104), 0 = Trust somewhat, very little or not at all (786)				
	**n**	**%**		**χ^2 (df)**	**P**	**N**	**AOR***	**95% CI Lower**	**95% CI Upper**	**P**
**Gender**										
Male	1957	81.7		3.020 (2)	.221	2097	ref			.526
Female	2046	79.9				2784	0.92	0.79	1.08	.331
Other	5	71.4				9	0.59	0.12	2.94	.518
**Age**										
18-29	649	78.8		53.883 (6)	<.001	459	ref			<.001
30-39 *v 18-29*	642	75.7				758	0.83	0.61	1.12	.219
40-49 *v 18-39*	629	78.4				835	0.96	0.76	1.22	.750
50-59 *v 18-49*	701	80.9				896	1.18	0.95	1.47	.133
60-69 *v 18-59*	599	84.2				1004	1.40	1.12	1.74	.003
70-79 *v 18-69*	582	89.0				761	1.72	1.32	2.24	<.001
80+ *v 18-79*	181	83.0				177	1.32	0.83	2.11	.240
**Education/Highest qualification**										
No qualifications	440	71.7		91.917 (4)	<.001	408	ref			<.001
Degree or equivalent and above	1775	85.7				2454	2.64	2.00	3.48	<.001
A levels / Vocational level 3 or equivalent	906	80.0				990	1.87	1.39	2.51	<.001
Other qual'ns below A level / Voc level 3	673	80.5				783	1.70	1.25	2.29	.001
Other qualification	210	69.1				255	0.97	0.67	1.40	.872
**Financial Status**										
Living comfortably	1121	87.2		124.251 (4)	<.001	1533	ref			<.001
Doing alright	1699	83.7				1995	0.90	0.73	1.10	.296
Just about getting by	824	72.9				959	0.61	0.48	0.77	<.001
Finding it quite difficult	247	73.3				266	0.60	0.42	0.84	.003
Finding it very difficult	116	66.7				137	0.51	0.33	0.78	.002
**Country**										
England	3499	81.7		21.523 (2)	<.001	4299	ref			.128
Scotland	321	73.0				383	0.77	0.59	1.02	.068
Wales	183	77.2				208	0.82	0.56	1.20	.308
**Urban/rural**										
Urban	3201	80.1		4.443 (1)	.035	3725	ref			
Rural	802	83.1				1165	0.97	0.80	1.18	.778
**Ethnicity**										
White British	3314	83.0		63.871 (5)	<.001	4224	ref			<.001
Any other white background	242	72.7				317	0.54	0.41	0.72	<.001
Mixed or multiple ethnic groups	64	66.0				62	0.38	0.22	0.67	<.001
Asian or Asian British	223	73.4				160	0.53	0.36	0.77	<.001
Black or Black British	70	69.3				67	0.41	0.24	0.70	.001
Other	58	71.6				60	0.56	0.30	1.03	.060
						Hosmer & Lemeshow χ^2= 10.236, df=8, p=0.249.<br>Final model χ^2=220.263, df=24, p<0.001<br>Nagelkerke = 0.075<br>Cases correctly classified: 84.0%.<br>88 cases excluded due to missing data on one or more independent variables.				

* adjusted for all other variables in the model, AOR, adjusted odds ratio; ref, reference category; 95% CI, 95% confidence interval.

For peer review only

Table S3 Pharmacists – Association between trust in sources of information about COVID-19 vaccine and socio-demographic variables – (a) bivariate results and (b) multivariate logistic regression.

	**(a) Bivariate associations between socio-demographics and trusting COVID-19 vaccine info from Pharmacists** % Trust completely or a great deal (weighted) χ^2 test for differences by demographics				**(b) Logistic regression of trust in COVID-19 vaccine info from Pharmacists** 1 = Trust completely or a great deal (3107), 0 = Trust somewhat, very little or not at all (1786)				
	**n**	**%**	**χ^2 (df)**	**P**	**N**	**AOR***	**95% CI Lower**	**95% CI Upper**	**P**
**Gender**									
Male	1420	59.2	1.597 [#]	.474	2098	ref			.620
Female	1548	60.4			2786	1.06	0.94	1.19	.376
Other	3	42.9			9	0.78	0.21	2.95	.715
**Age**									
18-29	461	55.9	29.783 (6)	<.001	459	ref			.092
30-39 *v 18-29*	486	57.2			759	0.98	0.77	1.24	.848
40-49 *v 18-39*	455	56.7			835	0.96	0.80	1.16	.662
50-59 *v 18-49*	518	59.7			896	1.18	0.99	1.39	.062
60-69 *v 18-59*	462	65.0			1004	1.24	1.06	1.46	.009
70-79 *v 18-69*	428	65.1			763	1.00	0.84	1.19	.972
80+ *v 18-79*	144	65.8			177	1.12	0.80	1.55	.514
**Education/Highest qualification**									
No qualifications	349	56.7	25.123 (4)	<.001	410	ref			<.001
Degree or equivalent and above	1305	63.0			2454	1.34	1.07	1.67	.012
A levels / Vocational level 3 or equivalent	680	60.1			990	1.20	0.94	1.53	.146
Other qual'ns below A level / Voc level 3	485	57.9			784	1.02	0.79	1.31	.890
Other qualification	151	49.8			255	0.82	0.59	1.13	.216
**Financial Status**									
Living comfortably	883	68.7	78.993 (4)	<.001	1533	ref			<.001
Doing alright	1220	60.0			1997	0.77	0.66	0.89	<.001
Just about getting by	607	53.6			960	0.63	0.53	0.75	<.001
Finding it quite difficult	178	52.8			266	0.62	0.47	0.82	.001
Finding it very difficult	81	46.8			137	0.51	0.35	0.73	<.001
**Country**									
England	2589	60.4	7.095 (2)	.029	4301	ref			.673
Scotland	239	54.1			384	0.91	0.73	1.13	.403
Wales	137	57.8			208	0.95	0.71	1.27	.721
**Urban/rural**									
Urban	2349	58.7	8.096 (1)	.004	3728				
Rural	615	63.7			1165	1.04	0.90	1.20	.599
**Ethnicity**									
White British	2516	62.9	91.005 (5)	<.001	4226	ref			<.001
Any other white background	157	46.7			318	0.57	0.45	0.72	<.001
Mixed or multiple ethnic groups	39	40.2			62	0.42	0.25	0.70	<.001
Asian or Asian British	142	46.6			160	0.47	0.34	0.65	<.001
Black or Black British	51	50.5			67	0.63	0.39	1.03	.067
Other	35	43.2			60	0.48	0.29	0.81	.006
					Hosmer & Lemeshow χ^2= 11.453, df=8, p=0.177.<br>Final model χ^2=157.815, df=24, p<0.001<br>Nagelkerke = .043<br>Cases correctly classified: 64.5%.<br>85 cases excluded due to missing data on one or more independent variables.				

* adjusted for all other variables in the model, AOR, adjusted odds ratio; ref, reference category; 95% CI, 95% confidence interval. [#] Fisher-Freeman-Halton Exact Test.

For peer review only

Table S4 The NHS – Association between trust in sources of information about COVID-19 vaccine and socio-demographic variables – (a) bivariate results and (b) multivariate logistic regression.

	**(a) Bivariate associations between socio-demographics and trusting COVID-19 vaccine info from the NHS** % Trust completely or a great deal (weighted) χ 2 test for differences by demographics					**(b) Logistic regression of trust in COVID-19 vaccine info from the NHS** 1 = Trust completely or a great deal (4115), 0 = Trust somewhat, very little or not at all (775)				
	**n**	**%**		**χ^2 (df)**	**P**	**N**	**AOR***	**95% CI Lower**	**95% CI Upper**	**P**
**Gender**										
Male	1926	80.3		1.598 (2)	.450	2097	ref			.461
Female	2055	80.2				2784	0.97	0.82	1.14	.718
Other	5	62.5				9	0.41	0.10	1.74	.225
**Age**										
18-29	609	73.9		106.785 (6)	<.001	459	ref			<.001
30-39 *v 18-29*	632	74.4				759	0.94	0.70	1.26	.672
40-49 *v 18-39*	618	77.1				834	1.05	0.84	1.32	.672
50-59 *v 18-49*	710	81.9				895	1.45	1.16	1.81	.001
60-69 *v 18-59*	609	85.7				1004	1.62	1.29	2.03	<.001
70-79 *v 18-69*	593	90.5				762	1.99	1.50	2.63	<.001
80+ *v 18-79*	186	85.3				177	1.66	0.99	2.79	.056
**Education/Highest qualification**										
No qualifications	447	72.6		60.407 (4)	<.001	410	ref			<.001
Degree or equivalent and above	1733	83.7				2452	2.39	1.80	3.16	<.001
A levels / Vocational level 3 or equivalent	909	80.4				990	1.95	1.44	2.64	<.001
Other qual'ns below A level / Voc level 3	679	81.2				783	1.78	1.30	2.43	<.001
Other qualification	211	69.6				255	0.95	0.65	1.39	.805
**Financial Status**										
Living comfortably	1136	88.3		167.221 (4)	<.001	1533	ref			<.001
Doing alright	1675	82.4				1996	0.74	0.59	0.92	.006
Just about getting by	823	72.8				958	0.47	0.37	0.60	<.001
Finding it quite difficult	248	73.6				266	0.51	0.36	0.72	<.001
Finding it very difficult	99	56.9				137	0.31	0.21	0.47	<.001
**Country**										
England	3459	80.8		6.736 (2)	.034	4298	ref			.505
Scotland	345	78.1				384	0.98	0.73	1.32	.900
Wales	177	74.7				208	0.80	0.54	1.17	.243
**Urban/rural**										
Urban	3165	79.2		14.722 (1)	<.001	3725				
Rural	817	84.7				1165	1.03	0.84	1.26	.791
**Ethnicity**										
White British	3331	83.3		126.307 (5)	<.001	4225	ref			<.001
Any other white background	224	67.3				317	0.47	0.36	0.63	<.001
Mixed or multiple ethnic groups	67	69.1				62	0.48	0.27	0.86	.014
Asian or Asian British	207	68.1				160	0.57	0.39	0.84	.004
Black or Black British	60	60.0				66	0.38	0.22	0.65	<.001
Other	57	70.4				60	0.45	0.25	0.81	.008
						Hosmer & Lemeshow χ^2= 8.677, df=8, p=0.370. Final model χ^2=291.002, df=24, p<0.001 Nagelkerke = .099 Cases correctly classified: 84.3%. 88 cases excluded due to missing data on one or more independent variables.				

* adjusted for all other variables in the model, AOR, adjusted odds ratio; ref, reference category; 95% CI, 95% confidence interval.

For peer review only - http://bmjopen.bmj.com/site/about/guidelines.xhtml

Table S5 Drug companies who manufacture vaccines – Association between trust in sources of information about COVID-19 vaccine and socio-demographic variables – (a) bivariate results and (b) multivariate logistic regression.

	**(a) Bivariate associations between socio-demographics and trusting COVID-19 vaccine info from drug companies who manufacture vaccines** % Trust completely or a great deal (weighted) χ^2 test for differences by demographics					**(b) Logistic regression of trust in COVID-19 vaccine info from drug companies who manufacture vaccines** 1 = Trust completely or a great deal (1416), 0 = Trust somewhat, very little or not at all (3473)				
	**n**	**%**		**χ^2 (df)**	**P**	**N**	**AOR***	**95% CI Lower**	**95% CI Upper**	**P**
**Gender**										
Male	652	27.2		16.276#	<.001	2096	ref			<.001
Female	819	32.0				2784	1.28	1.12	1.45	<.001
Other	0	0.0				9	0.00	0.00	.	.999
**Age**										
18-29	264	32.0		32.180 (6)	<.001	459	ref			.030
30-39 *v 18-29*	249	29.3				759	0.91	0.71	1.18	.480
40-49 *v 18-39*	196	24.5				834	0.72	0.59	0.89	.002
50-59 *v 18-49*	226	26.1				896	0.87	0.72	1.04	.120
60-69 *v 18-59*	219	30.8				1004	0.96	0.81	1.13	.613
70-79 *v 18-69*	233	35.6				760	1.12	0.94	1.34	.204
80+ *v 18-79*	76	34.7				177	1.02	0.73	1.42	.905
**Education/Highest qualification**										
No qualifications	217	35.3		17.807 (4)	.001	408	ref			.004
Degree or equivalent and above	569	27.5				2454	0.72	0.57	0.92	.007
A levels / Vocational level 3 or equivalent	321	28.4				990	0.78	0.60	1.01	.057
Other qual'ns below A level / Voc level 3	267	31.9				783	0.97	0.75	1.26	.844
Other qualification	97	32.1				254	0.94	0.67	1.32	.732
**Financial Status**										
Living comfortably	444	34.5		20.183 (4)	<.001	1533	ref			<.001
Doing alright	565	27.8				1995	0.72	0.62	0.83	<.001
Just about getting by	313	27.7				958	0.66	0.55	0.80	<.001
Finding it quite difficult	98	29.0				266	0.74	0.55	1.00	.054
Finding it very difficult	50	29.1				137	0.55	0.36	0.85	.007
**Country**										
England	1259	29.4		2.325 (2)	.313	4297	ref			.842
Scotland	130	29.3				384	1.00	0.79	1.26	.996
Wales	81	34.0				208	1.10	0.81	1.49	.559
**Urban/rural**										
Urban	1187	29.7		.092 (1)	.762	3724				
Rural	282	29.2				1165	0.99	0.85	1.15	.868
**Ethnicity**										
White British	1234	30.9		29.028 (5)	<.001	4223	ref			.012
Any other white background	70	20.9				318	0.63	0.48	0.84	.002
Mixed or multiple ethnic groups	20	20.6				62	0.92	0.52	1.62	.769
Asian or Asian British	83	27.2				160	0.74	0.50	1.08	.119
Black or Black British	35	34.7				67	1.11	0.64	1.91	.714
Other	12	15.2				59	0.55	0.28	1.07	.078
						Hosmer & Lemeshow χ^2= 9.180, df=8, p=0.327. Final model χ^2=96.401, df=24, p<0.001 Nagelkerke = .028 Cases correctly classified: 71.0%. 89 cases excluded due to missing data on one or more independent variables.				

* adjusted for all other variables in the model, AOR, adjusted odds ratio; ref, reference category; 95% CI, 95% confidence interval. # Fisher-Freeman-Halton Exact Test.

For peer review only - http://bmjopen.bmj.com/site/about/guidelines.xhtml

Table S6 The UK Government – Association between trust in sources of information about COVID-19 vaccine and socio-demographic variables – (a) bivariate results and (b) multivariate logistic regression.

	**(a) Bivariate associations between socio-demographics and trusting COVID-19 vaccine info from the UK Government** % Trust completely or a great deal (weighted) χ^2 test for differences by demographics					**(b) Logistic regression of trust in COVID-19 vaccine info from the UK Government** 1 = Trust completely or a great deal (2279), 0 = Trust somewhat, very little or not at all (2607)				
	**n**	**%**		**χ^2 (df)**	**P**	**N**	**AOR***	**95% CI Lower**	**95% CI Upper**	**P**
**Gender**										
Male	1114	46.6		10.393#	.005	2096	ref			.012
Female	1080	42.3				2781	0.84	0.75	0.95	.004
Other	2	25.0				9	0.47	0.09	2.37	.363
**Age**										
18-29	261	31.9		182.080 (6)	<.001	458	ref			<.001
30-39 *v 18-29*	290	34.2				758	1.14	0.89	1.47	.289
40-49 *v 18-39*	332	41.5				834	1.37	1.14	1.65	<.001
50-59 *v 18-49*	416	48.0				895	1.67	1.41	1.97	<.001
60-69 *v 18-59*	373	52.8				1003	1.46	1.25	1.70	<.001
70-79 *v 18-69*	383	58.6				762	1.65	1.40	1.96	<.001
80+ *v 18-79*	124	57.1				176	1.54	1.13	2.11	.007
**Education/Highest qualification**										
No qualifications	269	43.7		2.856 (4)	.582	410	ref			.439
Degree or equivalent and above	924	44.8				2450	0.95	0.76	1.18	.622
A levels / Vocational level 3 or equivalent	482	42.6				990	1.04	0.82	1.33	.733
Other qual'ns below A level / Voc level 3	386	46.2				783	1.07	0.83	1.37	.601
Other qualification	134	45.1				253	1.14	0.83	1.58	.422
**Financial Status**										
Living comfortably	704	54.8		94.512 (4)	<.001	1533	ref			<.001
Doing alright	891	43.9				1994	0.76	0.66	0.87	<.001
Just about getting by	415	36.9				957	0.54	0.45	0.64	<.001
Finding it quite difficult	124	36.9				266	0.55	0.42	0.73	<.001
Finding it very difficult	60	36.1				136	0.40	0.27	0.59	<.001
**Country**										
England	1928	45.1		19.887 (2)	<.001	4295	ref			.003
Scotland	151	34.5				383	0.70	0.56	0.88	.002
Wales	114	48.3				208	1.18	0.89	1.58	.253
**Urban/rural**										
Urban	1741	43.8		2.886 (1)	.089	3721				
Rural	451	46.8				1165	1.00	0.87	1.15	.969
**Ethnicity**										
White British	1832	45.9		35.180 (5)	<.001	4224	ref			.074
Any other white background	104	31.2				317	0.70	0.55	0.90	.005
Mixed or multiple ethnic groups	31	32.0				62	0.73	0.43	1.26	.258
Asian or Asian British	126	42.6				158	1.14	0.82	1.59	.430
Black or Black British	40	40.0				66	1.07	0.64	1.77	.803
Other	31	39.2				59	1.03	0.60	1.76	.910
						Hosmer & Lemeshow χ^2= 9.412, df=8, p=0.309. Final model χ^2=258.301, df=24, p<0.001 Nagelkerke = .069 Cases correctly classified: 60.3%. 92 cases excluded due to missing data on one or more independent variables.				

* adjusted for all other variables in the model, AOR, adjusted odds ratio; ref, reference category; 95% CI, 95% confidence interval. # Fisher-Freeman-Halton Exact Test.

For peer review only - http://bmjopen.bmj.com/site/about/guidelines.xhtml

Table S7 The Scottish/Welsh Government – Association between trust in sources of information about COVID-19 vaccine and socio-demographic variables – (a) bivariate results and (b) multivariate logistic regression.

	**(a) Bivariate associations between socio-demographics and trusting COVID-19 vaccine info from the Scottish/Welsh Government** % Trust completely or a great deal (weighted) χ^2 test for differences by demographics					**(b) Logistic regression of trust in COVID-19 vaccine info from the Scottish/Welsh Government** 1 = Trust completely or a great deal (289), 0 = Trust somewhat, very little or not at all (297)				
	**n**	**%**		**χ^2 (df)**	**P**	**N**	**AOR***	**95% CI Lower**	**95% CI Upper**	**P**
**Gender**										
Male	173	49.0		3.935 (1)	.047	274	ref			
Female	132	41.4				312	0.89	0.63	1.25	.491
**Age**										
18-29	61	50.8		8.885 (6)	.180	63	ref			.249
30-39 *v 18-29*	45	44.1				93	1.27	0.65	2.48	.485
40-49 *v 18-39*	36	36.7				95	0.87	0.51	1.49	.617
50-59 *v 18-49*	75	52.1				117	1.66	1.03	2.67	.037
60-69 *v 18-59*	46	46.5				113	1.06	0.67	1.68	.790
70-79 *v 18-69*	32	43.8				81	0.78	0.46	1.32	.353
80+ *v 18-79*	10	33.3				24	0.63	0.26	1.50	.297
**Education/Highest qualification**										
No qualifications	29	27.9		31.212 (4)	<.001	52	ref			.042
Degree or equivalent and above	144	56.7				280	2.18	1.12	4.23	.021
A levels / Vocational level 3 or equivalent	79	47.3				135	1.95	0.97	3.95	.062
Other qual'ns below A level / Voc level 3	34	36.6				81	1.27	0.60	2.69	.538
Other qualification	18	35.3				38	1.03	0.42	2.53	.941
**Financial Status**										
Living comfortably	84	56.0		8.356 (4)	.079	167	ref			.153
Doing alright	118	42.4				256	0.69	0.46	1.05	.081
Just about getting by	71	43.3				114	0.62	0.37	1.05	.074
Finding it quite difficult	22	41.5				34	0.49	0.22	1.09	.078
Finding it very difficult	11	45.8				15	0.34	0.10	1.16	.083
**Country**										
Scotland	206	46.9		1.208 (1)	.272	381	ref			
Wales	99	42.5				205	1.09	0.76	1.57	.642
**Urban/rural**										
Urban	233	46.9		1.719 (1)	.190	409				
Rural	72	41.1				177	0.62	0.42	0.91	.015
**Ethnicity**										
White British	270	45.3		.147 (1)	.702	535	ref			
Other than white British	32	47.8				51	1.03	0.55	1.92	.932
						Hosmer & Lemeshow χ^2= 12.017, df=8, p=0.150.<br>Final model χ^2=35.151, df=18, p=0.009<br>Nagelkerke = .078<br>Cases correctly classified: 61.4%.<br>16 cases excluded due to missing data on one or more independent variables.				

* adjusted for all other variables in the model, AOR, adjusted odds ratio; ref, reference category; 95% CI, 95% confidence interval. # Fisher-Freeman-Halton Exact Test.

For peer review only

Table S8 Scientific and medical advisers – Association between trust in sources of information about COVID-19 vaccine and socio-demographic variables – (a) bivariate results and (b) multivariate logistic regression.

	(a) Bivariate associations between socio-demographics and trusting COVID-19 vaccine info from scientific and medical advisers<br>% Trust completely or a great deal (weighted)<br>χ^2 test for differences by demographics					(b) Logistic regression of trust in COVID-19 vaccine info from scientific and medical advisers<br>1 = Trust completely or a great deal (4008), 0 = Trust somewhat, very little or not at all (884)				
	**n**	**%**		**χ^2 (df)**	**P**	**N**	**AOR***	**95% CI Lower**	**95% CI Upper**	**P**
**Gender**										
Male	1885	78.6		0.302 (2)	.860	2097	ref			.544
Female	2006	78.2				2786	1.08	0.93	1.26	.320
Other	5	71.4				9	0.71	0.14	3.51	.674
**Age**										
18-29	644	78.2		11.885 (6)	.065	459	ref			.850
30-39 *v 18-29*	643	75.6				759	0.86	0.63	1.17	.323
40-49 *v 18-39*	631	78.6				835	1.06	0.84	1.34	.640
50-59 *v 18-49*	677	78.1				896	1.09	0.88	1.36	.426
60-69 *v 18-59*	572	80.5				1004	1.03	0.84	1.26	.785
70-79 *v 18-69*	540	82.2				763	1.05	0.84	1.32	.654
80+ *v 18-79*	166	76.1				176	1.10	0.72	1.68	.650
**Education/Highest qualification**										
No qualifications	389	63.1		147.739 (4)	<.001	410	ref			<.001
Degree or equivalent and above	1743	84.1				2453	3.21	2.50	4.13	<.001
A levels / Vocational level 3 or equivalent	905	80.0				990	2.70	2.04	3.55	<.001
Other qual'ns below A level / Voc level 3	653	77.8				784	1.98	1.51	2.61	<.001
Other qualification	205	67.7				255	1.23	0.87	1.73	.245
**Financial Status**										
Living comfortably	1110	86.4		154.081 (4)	<.001	1532	ref			<.001
Doing alright	1640	80.6				1997	0.65	0.53	0.80	<.001
Just about getting by	797	70.4				960	0.46	0.36	0.58	<.001
Finding it quite difficult	249	73.9				266	0.51	0.36	0.72	<.001
Finding it very difficult	97	55.7				137	0.32	0.21	0.48	<.001
**Country**										
England	3375	78.8		3.260 (2)	.196	4300	ref			.068
Scotland	341	77.0				384	1.03	0.77	1.37	.843
Wales	176	74.3				208	0.67	0.47	0.94	.022
**Urban/rural**										
Urban	3108	77.7		5.368 (1)	.021	3727				
Rural	783	81.1				1165	1.00	0.83	1.20	.978
**Ethnicity**										
White British	3236	80.9		87.036 (5)	<.001	4225	ref			<.001
Any other white background	244	72.8				318	0.56	0.42	0.75	<.001
Mixed or multiple ethnic groups	68	70.8				62	0.51	0.28	0.92	.025
Asian or Asian British	203	66.6				160	0.46	0.32	0.67	<.001
Black or Black British	57	56.4				67	0.31	0.19	0.53	<.001
Other	53	65.4				60	0.43	0.24	0.76	.004
						Hosmer & Lemeshow χ^2= 5.496, df=8, p=0.704.<br>Final model χ^2=268.594, df=24, p<0.001<br>Nagelkerke = .087<br>Cases correctly classified: 82.1%.<br>86 cases excluded due to missing data on one or more independent variables.				

* adjusted for all other variables in the model, AOR, adjusted odds ratio; ref, reference category; 95% CI, 95% confidence interval.

For peer review only

Table S9 The World Health Organisation (WHO) – Association between trust in sources of information about COVID-19 vaccine and socio-demographic variables – (a) bivariate results and (b) multivariate logistic regression.

	**(a) Bivariate associations between socio-demographics and trusting COVID-19 vaccine info from The World Health Organisation (WHO)** % Trust completely or a great deal (weighted) χ^2 test for differences by demographics					**(b) Logistic regression of trust in COVID-19 vaccine info from The World Health Organisation (WHO)** 1 = Trust completely or a great deal (3423), 0 = Trust somewhat, very little or not at all (1468)				
	**n**	**%**		**χ^2 (df)**	**P**	**N**	**AOR***	**95% CI Lower**	**95% CI Upper**	**P**
**Gender**										
Male	1523	63.7		23.303[#]	<.001	2097	ref			<.001
Female	1797	70.1				2785	1.49	1.31	1.69	<.001
Other	5	71.4				9	1.92	0.39	9.42	.420
**Age**										
18-29	556	68.1		5.005 (6)	.543	458	ref			.177
30-39 *v 18-29*	549	64.5				760	0.84	0.65	1.10	.203
40-49 *v 18-39*	538	67.1				835	0.98	0.81	1.20	.882
50-59 *v 18-49*	593	68.4				896	1.15	0.96	1.39	.126
60-69 *v 18-59*	484	68.1				1004	0.89	0.76	1.06	.189
70-79 *v 18-69*	450	68.6				762	0.87	0.73	1.04	.127
80+ *v 18-79*	140	64.8				176	0.86	0.62	1.20	.366
**Education/Highest qualification**										
No qualifications	351	56.9		75.592 (4)	<.001	410	ref			<.001
Degree or equivalent and above	1490	72.3				2452	1.73	1.38	2.18	<.001
A levels / Vocational level 3 or equivalent	758	67.0				990	1.39	1.08	1.77	.010
Other qual'ns below A level / Voc level 3	561	66.9				784	1.36	1.06	1.76	.017
Other qualification	166	54.6				255	0.88	0.64	1.21	.428
**Financial Status**										
Living comfortably	953	74.2		67.486 (4)	<.001	1533	ref			<.001
Doing alright	1384	68.2				1995	0.78	0.66	0.91	.002
Just about getting by	691	61.0				960	0.60	0.50	0.73	<.001
Finding it quite difficult	204	60.4				266	0.56	0.42	0.75	<.001
Finding it very difficult	92	54.8				137	0.45	0.31	0.65	<.001
**Country**										
England	2880	67.4		2.028 (2)	.363	-				
Scotland	283	64.0				-	-	-	-	-
Wales	160	67.5				-	-	-	-	-
**Urban/rural**										
Urban	2663	66.7		1.288 (1)	.256	3727				
Rural	660	68.6				1164	0.99	0.85	1.15	.897
**Ethnicity**										
White British	2737	68.5		30.713 (5)	<.001	4224	ref			<.001
Any other white background	223	66.4				318	0.75	0.58	0.96	.023
Mixed or multiple ethnic groups	59	61.5				62	0.63	0.37	1.07	.089
Asian or Asian British	171	57.2				160	0.64	0.46	0.90	.010
Black or Black British	55	53.9				67	0.44	0.27	0.72	.001
Other	45	56.3				60	0.61	0.36	1.04	.068
						Hosmer & Lemeshow χ^2= 14.933, df=8, p=0.060.<br>Final model χ^2=172.240, df=22, p<0.001<br>Nagelkerke = .049<br>Cases correctly classified: 70.0%.<br>87 cases excluded due to missing data on one or more independent variables.				

* adjusted for all other variables in the model, AOR, adjusted odds ratio; ref, reference category; 95% CI, 95% confidence interval. [#] Fisher-Freeman-Halton Exact Test. Country was excluded from the logistic regression to achieve model fit.

For peer review only - http://bmjopen.bmj.com/site/about/guidelines.xhtml

Table S10 The media (e.g. newspapers, magazines, television, radio) – Association between trust in sources of information about COVID-19 vaccine and socio-demographic variables – (a) bivariate results and (b) multivariate logistic regression.

	**(a) Bivariate associations between socio-demographics and trusting COVID-19 vaccine info from the media** % Trust completely or a great deal (weighted) χ^2 test for differences by demographics					**(b) Logistic regression of trust in COVID-19 vaccine info from the media** 1 = Trust completely or a great deal (361), 0 = Trust somewhat, very little or not at all (4530)				
	**n**	**%**		**χ^2 (df)**	**P**	**N**	**AOR***	**95% CI Lower**	**95% CI Upper**	**P**
**Gender**										
Male	176	7.3		2.193 (2)	.334	2097				.575
Female	213	8.3				2785	1.13	0.90	1.41	.293
Other	0	0.0				9	0.00	0.00		.999
**Age**										
18-29	57	6.9		39.450 (6)	<.001	459				.003
30-39 *v 18-29*	57	6.7				759	0.84	0.52	1.35	.462
40-49 *v 18-39*	52	6.5				834	1.04	0.72	1.49	.848
50-59 *v 18-49*	58	6.7				895	1.08	0.78	1.50	.629
60-69 *v 18-59*	49	6.9				1004	1.05	0.77	1.42	.761
70-79 *v 18-69*	85	12.9				763	1.75	1.32	2.33	<.001
80+ *v 18-79*	29	13.3				177	1.74	1.07	2.83	.024
**Education/Highest qualification**										
No qualifications	77	12.5		34.152 (4)	<.001	409				.005
Degree or equivalent and above	153	7.4				2454	0.63	0.44	0.90	.011
A levels / Vocational level 3 or equivalent	58	5.1				990	0.44	0.29	0.68	<.001
Other qual'ns below A level / Voc level 3	79	9.4				784	0.72	0.48	1.07	.102
Other qualification	21	7.0				254	0.56	0.31	1.00	.051
**Financial Status**										
Living comfortably	105	8.2		34.041 (4)	<.001	1533				.671
Doing alright	122	6.0				1996	0.90	0.69	1.16	.413
Just about getting by	99	8.8				959	1.01	0.73	1.39	.974
Finding it quite difficult	33	9.8				266	1.22	0.75	1.98	.430
Finding it very difficult	30	17.2				137	0.79	0.37	1.68	.535
**Country**										
England	336	7.8		.515 (2)	.773	4300				.457
Scotland	32	7.3				383	0.79	0.51	1.24	.313
Wales	21	8.8				208	1.19	0.71	2.00	.499
**Urban/rural**										
Urban	323	8.1		1.957 (1)	.162	3726				
Rural	65	6.7				1165	0.91	0.70	1.19	.486
**Ethnicity**										
White British	296	7.4		6.645 (5)	.248	4226				.073
Any other white background	30	9.0				318	1.55	1.03	2.32	.035
Mixed or multiple ethnic groups	6	6.2				62	1.31	0.52	3.33	.570
Asian or Asian British	30	9.9				160	1.73	1.02	2.94	.043
Black or Black British	12	11.9				66	1.84	0.82	4.12	.139
Other	8	10.3				59	1.62	0.68	3.85	.274
						Hosmer & Lemeshow χ^2= 2.359, df=8, p=0.968.<br>Final model χ^2=54.051, df=24, p<0.001<br>Nagelkerke = .027<br>Cases correctly classified: 92.6%.<br>87 cases excluded due to missing data on one or more independent variables.				

* adjusted for all other variables in the model, AOR, adjusted odds ratio; ref, reference category; 95% CI, 95% confidence interval.

For peer review only - http://bmjopen.bmj.com/site/about/guidelines.xhtml

Table S11 Social media – Association between trust in sources of information about COVID-19 vaccine and socio-demographic variables – (a) bivariate results and (b) multivariate logistic regression.

	(a) Bivariate associations between socio-demographics and trusting COVID-19 vaccine info from social media<br>% Trust completely or a great deal (weighted)<br>χ^2 test for differences by demographics					(b) Logistic regression of trust in COVID-19 vaccine info from social media<br>1 = Trust completely or a great deal (95), 0 = Trust somewhat, very little or not at all (4792)				
	**n**	**%**		**χ^2 (df)**	**P**	**N**	**AOR***	**95% CI Lower**	**95% CI Upper**	**P**
**Gender**										
Male	63	2.6		0.284 (2)	.868	2094	ref			.845
Female	71	2.8				2784	1.13	0.74	1.73	.561
Other	0	0.0				9	0.00	0.00		.999
**Age**										
18-29	31	3.8		12.626 (6)	.049	459	ref			.634
30-39 *v 18-29*	25	2.9				759	0.55	0.24	1.22	.139
40-49 *v 18-39*	22	2.7				835	0.89	0.47	1.67	.713
50-59 *v 18-49*	13	1.5				896	0.85	0.47	1.55	.596
60-69 *v 18-59*	13	1.8				1003	0.71	0.39	1.29	.259
70-79 *v 18-69*	19	2.9				761	1.11	0.62	1.97	.727
80+ *v 18-79*	9	4.2				174	1.26	0.49	3.25	.631
**Education/Highest qualification**										
No qualifications	31	5.1		24.978 (4)	<.001	409	ref			<.001
Degree or equivalent and above	44	2.1				2452	0.24	0.13	0.44	<.001
A levels / Vocational level 3 or equivalent	18	1.6				989	0.32	0.16	0.64	.001
Other qual'ns below A level / Voc level 3	32	3.8				782	0.50	0.26	0.94	.031
Other qualification	8	2.6				255	0.46	0.18	1.18	.106
**Financial Status**										
Living comfortably	34	2.6		26.413 (4)	<.001	1531	ref			.522
Doing alright	38	1.9				1994	0.73	0.43	1.24	.241
Just about getting by	35	3.1				959	0.99	0.55	1.79	.976
Finding it quite difficult	12	3.6				266	1.35	0.60	3.04	.473
Finding it very difficult	14	8.1				137	0.81	0.23	2.84	.746
**Country**										
England	118	2.8		.404(2)	.817	4295	ref			.215
Scotland	10	2.3				384	0.40	0.12	1.27	.120
Wales	6	2.5				208	1.42	0.56	3.58	.463
**Urban/rural**										
Urban	122	3.1		9.660(1)	.002	3724				
Rural	12	1.2				1163	0.57	0.31	1.05	.071
**Ethnicity**										
White British	94	2.4		17.781#	.002	4220	ref			.326
Any other white background	7	2.1				318	1.00	0.39	2.53	.994
Mixed or multiple ethnic groups	4	4.1				62	2.52	0.75	8.45	.134
Asian or Asian British	14	4.6				160	1.85	0.77	4.45	.172
Black or Black British	9	8.8				69	2.57	0.76	8.64	.128
Other	1	1.2				60	0.83	0.11	6.20	.855
						Hosmer & Lemeshow χ^2= 5.858, df=8, p=0.663.<br>Final model χ^2=46.839, df=24, p=0.004<br>Nagelkerke = .055<br>Cases correctly classified: 98.1%.<br>91 cases excluded due to missing data on one or more independent variables.				

* adjusted for all other variables in the model, AOR, adjusted odds ratio; ref, reference category; 95% CI, 95% confidence interval. # Fisher-Freeman-Halton Exact Test.

For peer review only

Table S12 Celebrities and social media influencers – Association between trust in sources of information about COVID-19 vaccine and socio-demographic variables – (a) bivariate results and (b) multivariate logistic regression.

	**(a) Bivariate associations between socio-demographics and trusting COVID-19 vaccine info from celebrities and social media influencers** % Trust completely or a great deal (weighted) χ^2 test for differences by demographics					**(b) Logistic regression of trust in COVID-19 vaccine info from celebrities and social media influencers** 1 = Trust completely or a great deal (95), 0 = Trust somewhat, very little or not at all (4795)				
	**n**	**%**		**χ^2 (df)**	**P**	**N**	**AOR***	**95% CI Lower**	**95% CI Upper**	**P**
**Gender**										
Male	57	2.4		1.430 (2)	.489	2097	ref			.996
Female	74	2.9				2784	0.98	0.65	1.49	.928
Other	0	0.0				9	0.00	0.00		.999
**Age**										
18-29	24	2.9		19.156 (6)	.004	459	ref			.348
30-39 *v 18-29*	25	3.0				758	0.90	0.37	2.21	.818
40-49 *v 18-39*	23	2.9				835	1.11	0.58	2.14	.744
50-59 *v 18-49*	18	2.1				895	1.28	0.73	2.25	.390
60-69 *v 18-59*	9	1.3				1004	0.65	0.34	1.25	.192
70-79 *v 18-69*	17	2.6				762	1.26	0.70	2.28	.441
80+ *v 18-79*	14	6.4				177	2.18	0.96	4.98	.064
**Education/Highest qualification**										
No qualifications	35	5.7		58.886 (4)	<.001	409	ref			.002
Degree or equivalent and above	32	1.5				2453	0.31	0.17	0.58	<.001
A levels / Vocational level 3 or equivalent	13	1.1				990	0.31	0.15	0.65	.002
Other qual'ns below A level / Voc level 3	41	4.9				783	0.59	0.31	1.11	.103
Other qualification	10	3.3				255	0.57	0.24	1.40	.221
**Financial Status**										
Living comfortably	23	1.8		53.820 (4)	<.001	1533	ref			.022
Doing alright	27	1.3				1995	1.01	0.56	1.82	.975
Just about getting by	59	5.2				959	2.08	1.13	3.80	.018
Finding it quite difficult	11	3.3				266	2.47	1.08	5.64	.032
Finding it very difficult	10	5.7				137	1.86	0.60	5.77	.284
**Country**										
England	117	2.7		11.948 (2)	.003	4299	ref			.028
Scotland	3	0.7				383	0.40	0.13	1.29	.127
Wales	12	5.0				208	2.26	1.06	4.82	.036
**Urban/rural**										
Urban	119	3.0		9.096 (1)	.003	3725				
Rural	12	1.2				1165	0.64	0.36	1.14	.133
**Ethnicity**										
White British	94	2.4		14.208#	.008	4224	ref			.574
Any other white background	11	3.3				318	1.62	0.75	3.47	.217
Mixed or multiple ethnic groups	1	1.0				62	0.84	0.11	6.27	.866
Asian or Asian British	10	3.3				160	1.58	0.61	4.09	.346
Black or Black British	9	8.9				66	2.36	0.70	7.94	.166
Other	1	1.2				60	0.94	0.13	7.04	.956
						Hosmer & Lemeshow χ^2= 9.111, df=8, p=0.333. Final model χ^2=57.132, df=24, p<0.001 Nagelkerke = .067 Cases correctly classified: 98.1%. 88 cases excluded due to missing data on one or more independent variables.				

* adjusted for all other variables in the model, AOR, adjusted odds ratio; ref, reference category; 95% CI, 95% confidence interval.

For peer review only - http://bmjopen.bmj.com/site/about/guidelines.xhtml

Table S13 Family and friends – Association between trust in sources of information about COVID-19 vaccine and socio-demographic variables – (a) bivariate results and (b) multivariate logistic regression.

	**(a) Bivariate associations between socio-demographics and trusting COVID-19 vaccine info from family and friends** % Trust completely or a great deal (weighted) χ^2 test for differences by demographics					**(b) Logistic regression of trust in COVID-19 vaccine info from family and friends** 1 = Trust completely or a great deal (1139), 0 = Trust somewhat, very little or not at all (3752)				
	**n**	**%**		**χ^2 (df)**	**P**	**N**	**AOR***	**95% CI Lower**	**95% CI Upper**	**P**
**Gender**										
Male	557	23.2		4.455 (2)	.108	2098	ref			.053
Female	660	25.8				2784	1.17	1.01	1.34	.030
Other	2	28.6				9	2.39	0.58	9.85	.227
**Age**										
18-29	140	17.0		109.226 (6)	<.001	459	ref			<.001
30-39 *v 18-29*	194	22.8				759	1.28	0.94	1.73	.112
40-49 *v 18-39*	186	23.3				834	1.18	0.94	1.47	.157
50-59 *v 18-49*	182	21.0				896	0.93	0.76	1.15	.504
60-69 *v 18-59*	176	24.8				1004	1.11	0.92	1.33	.283
70-79 *v 18-69*	233	35.6				762	1.85	1.54	2.23	<.001
80+ *v 18-79*	90	41.1				177	2.33	1.69	3.20	<.001
**Education/Highest qualification**										<.001
No qualifications	203	33.0		70.692 (4)	<.001	410	ref			<.001
Degree or equivalent and above	403	19.4				2454	0.61	0.47	0.77	<.001
A levels / Vocational level 3 or equivalent	271	23.9				990	0.81	0.62	1.05	.112
Other qual'ns below A level / Voc level 3	254	30.4				783	0.99	0.76	1.29	.925
Other qualification	86	28.5				254	0.96	0.68	1.36	.812
**Financial Status**										
Living comfortably	330	25.7		6.383 (4)	.172	1533	ref			.667
Doing alright	470	23.1				1997	0.94	0.79	1.10	.424
Just about getting by	300	26.6				958	1.00	0.81	1.22	.976
Finding it quite difficult	81	24.0				266	1.03	0.75	1.42	.856
Finding it very difficult	38	21.8				137	0.75	0.47	1.18	.212
**Country**										
England	1051	24.6		13.592 (2)	.001	4299	ref			.012
Scotland	89	20.1				384	1.00	0.77	1.29	.976
Wales	78	32.9				208	1.59	1.17	2.17	.003
**Urban/rural**										
Urban	979	24.5		.029 (1)	.866	3726				
Rural	239	24.8				1165	0.91	0.77	1.07	.237
**Ethnicity**										
White British	1005	25.1		36.523 (5)	<.001	4226	ref			.029
Any other white background	50	15.0				317	0.79	0.58	1.07	.128
Mixed or multiple ethnic groups	15	15.5				62	0.76	0.38	1.51	.428
Asian or Asian British	100	32.8				160	1.52	1.06	2.18	.024
Black or Black British	27	26.7				67	1.39	0.79	2.46	.250
Other	12	15.2				59	0.53	0.24	1.18	.122
						Hosmer & Lemeshow χ^2= 6.067, df=8, p=0.640.<br>Final model χ^2=153.732, df=24, p<0.001<br>Nagelkerke = .047<br>Cases correctly classified: 76.7%.<br>87 cases excluded due to missing data on one or more independent variables.				

* adjusted for all other variables in the model, AOR, adjusted odds ratio; ref, reference category; 95% CI, 95% confidence interval.

For peer review only - http://bmjopen.bmj.com/site/about/guidelines.xhtml

Table S14 Faith or community leaders – Association between trust in sources of information about COVID-19 vaccine and socio-demographic variables – (a) bivariate results and (b) multivariate logistic regression.

	**(a) Bivariate associations between socio-demographics and trusting COVID-19 vaccine info from faith and community leaders** % Trust completely or a great deal (weighted) χ^2 test for differences by demographics					**(b) Logistic regression of trust in COVID-19 vaccine info from faith and community leaders** 1 = Trust completely or a great deal (161), 0 = Trust somewhat, very little or not at all (4724)				
	**n**	**%**		**χ^2 (df)**	**P**	**N**	**AOR***	**95% CI Lower**	**95% CI Upper**	**P**
**Gender**										
Male	118	4.9		17.452 (2)	<.001	2095	ref			.006
Female	135	5.3				2781	1.19	0.86	1.66	.294
Other	3	37.5				9	14.06	2.67	73.92	.002
**Age**										
18-29	47	5.7		20.879 (6)	.002	459	ref			.041
30-39 *v 18-29*	46	5.4				758	0.95	0.47	1.93	.880
40-49 *v 18-39*	31	3.9				834	1.11	0.65	1.89	.710
50-59 *v 18-49*	46	5.3				895	1.70	1.09	2.65	.020
60-69 *v 18-59*	20	2.8				1003	0.98	0.61	1.58	.933
70-79 *v 18-69*	42	6.4				760	1.62	1.03	2.55	.038
80+ *v 18-79*	20	9.2				176	2.28	1.15	4.56	.019
**Education/Highest qualification**										
No qualifications	57	9.3		37.137 (4)	<.001	407	ref			.011
Degree or equivalent and above	76	3.7				2451	0.42	0.25	0.71	<.001
A levels / Vocational level 3 or equivalent	51	4.5				990	0.57	0.33	0.99	.048
Other qual'ns below A level / Voc level 3	58	6.9				782	0.75	0.44	1.28	.290
Other qualification	15	4.9				255	0.50	0.22	1.14	.098
**Financial Status**										
Living comfortably	59	4.6		39.487 (4)	<.001	1530	ref			.042
Doing alright	71	3.5				1997	0.91	0.59	1.39	.663
Just about getting by	84	7.5				955	1.46	0.92	2.33	.110
Finding it quite difficult	20	5.9				266	2.05	1.09	3.84	.025
Finding it very difficult	20	11.5				137	1.44	0.60	3.44	.417
**Country**										
England	239	5.6		12.569 (2)	.002	4294	ref			.592
Scotland	11	2.5				383	0.69	0.33	1.43	.316
Wales	5	2.1				208	1.07	0.46	2.48	.877
**Urban/rural**										
Urban	229	5.7		13.640 (1)	<.001	3722				
Rural	27	2.8				1163	0.66	0.42	1.03	.068
**Ethnicity**										
White British	163	4.1		152.072(5)	<.001	4219	ref			<.001
Any other white background	12	3.6				318	1.22	0.60	2.46	.583
Mixed or multiple ethnic groups	3	3.1				62	2.59	0.90	7.42	.077
Asian or Asian British	59	19.6				159	4.82	2.76	8.42	<.001
Black or Black British	12	11.9				67	4.52	2.04	9.99	<.001
Other	2	2.5				60	1.37	0.32	5.77	.669
						Hosmer & Lemeshow χ^2= 11.202, df=8, p=0.191.<br>Final model χ^2=87.282, df=24, p<0.001<br>Nagelkerke = .070<br>Cases correctly classified: 96.7%.<br>93 cases excluded due to missing data on one or more independent variables.				

* adjusted for all other variables in the model, AOR, adjusted odds ratio; ref, reference category; 95% CI, 95% confidence interval.

For peer review only

For peer review only - http://bmjopen.bmj.com/site/about/guidelines.xhtml

Supplementary material to 'A national survey of attitudes towards and intentions to vaccinate against COVID-19: implications for communications'.

Checklist: STROBE Statement – items that should be included in reports of cross-sectional studies

	Item No	Recommendation	Location where item is reported
**Title and abstract**	1	(*a*) Indicate the study's design with a commonly used term in the title or the abstract	Title and abstract
		(*b*) Provide in the abstract an informative and balanced summary of what was done and what was found	Abstract
**Introduction**			
Background/rationale	2	Explain the scientific background and rationale for the investigation being reported	Introduction
Objectives	3	State specific objectives, including any prespecified hypotheses	Introduction (third paragraph). As this was the first survey of this type which addressed actual, rather than hypothetical, vaccines; we conducted an exploratory investigation rather than testing hypotheses.
**Methods**			
Study design	4	Present key elements of study design early in the paper	Methods (opening paragraph)
Setting	5	Describe the setting, locations, and relevant dates, including periods of recruitment, exposure, follow-up, and data collection	Methods: Sample and data collection
Participants	6	(*a*) Give the eligibility criteria, and the sources and methods of selection of participants	Methods: Sample and data collection
Variables	7	Clearly define all outcomes, exposures, predictors, potential confounders, and effect modifiers. Give diagnostic criteria, if applicable	Methods: Measures
Data sources/ measurement	8*	For each variable of interest, give sources of data and details of methods of assessment (measurement). Describe comparability of assessment methods if there is more than one group	Methods: Measures
Bias	9	Describe any efforts to address potential sources of bias	Methods: Sample and data collection (Survey used a probability sample; participants accessed online or by telephone, sent reminders–addressing response bias)
Study size	10	Explain how the study size was arrived at	Methods: Sample and data collection
Quantitative variables	11	Explain how quantitative variables were handled in the analyses. If applicable, describe which groupings were chosen and why	Methods: Data analysis
Statistical methods	12	(*a*) Describe all statistical methods, including those used to control for confounding	Methods: Data analysis
		(*b*) Describe any methods used to examine subgroups and interactions	Methods: Data analysis
		(*c*) Explain how missing data were addressed	Methods: Data analysis
		(*d*) If applicable, describe analytical methods taking account of sampling strategy	Methods: Data analysis
		(*e*) Describe any sensitivity analyses	Not applicable
**Results**			
Participants	13*	(a) Report numbers of individuals at each stage of study—eg numbers potentially eligible, examined for eligibility, confirmed eligible, included in the study, completing follow-up, and analysed	Table S1
		(b) Give reasons for non-participation at each stage	Table S1
		(c) Consider use of a flow diagram	Not required
Descriptive data	14*	(a) Give characteristics of study participants (eg demographic, clinical, social) and information on exposures and potential confounders	Results: Sample characteristics; and Table 1
		(b) Indicate number of participants with missing data for each variable of interest	Tables 2-4 and S2-S14
Outcome data	15*	Report numbers of outcome events or summary measures	Tables 2-4 and S2-S14
Main results	16	(*a*) Give unadjusted estimates and, if applicable, confounder-adjusted estimates and their precision (eg, 95% confidence interval). Make clear which confounders were adjusted for and why they were included	Results; Tables 3-4 and S2-S14
		(*b*) Report category boundaries when continuous variables were categorized	Not applicable (all variables were categorical)
		(*c*) If relevant, consider translating estimates of relative risk into absolute risk for a meaningful time period	Not applicable

For peer review only - http://bmjopen.bmj.com/site/about/guidelines.xhtml

	Item No	Recommendation	Location where item is reported
Other analyses	17	Report other analyses done—eg analyses of subgroups and interactions, and sensitivity analyses	Tables S2-S14 (subgroup analyses of the 13 COVID-19 vaccine information sources)
**Discussion**			
Key results	18	Summarise key results with reference to study objectives	Discussion: Principal findings
Limitations	19	Discuss limitations of the study, taking into account sources of potential bias or imprecision. Discuss both direction and magnitude of any potential bias	Discussion: Strengths and limitations
Interpretation	20	Give a cautious overall interpretation of results considering objectives, limitations, multiplicity of analyses, results from similar studies, and other relevant evidence	Discussion: Comparison with other studies; and Discussion: Strengths and limitations
Generalisability	21	Discuss the generalisability (external validity) of the study results	Discussion: Strengths and limitations
**Other information**			
Funding	22	Give the source of funding and the role of the funders for the present study and, if applicable, for the original study on which the present article is based	Role of the funding source

*Give information separately for exposed and unexposed groups.

For peer review only

BMJ Open

A national survey of attitudes towards and intentions to vaccinate against COVID-19: implications for communications

Journal:	*BMJ Open*
Manuscript ID	bmjopen-2021-055085.R1
Article Type:	Original research
Date Submitted by the Author:	14-Sep-2021
Complete List of Authors:	Stead, Martine; University of Stirling Institute for Social Marketing and Health<br>Jessop, Curtis; NatCen Social Research<br>Angus, Kathryn; University of Stirling Institute for Social Marketing and Health<br>Bedford, Helen; UCL Great Ormond Street Institute of Child Health Population Policy and Practice<br>Ussher, Michael; University of Stirling Institute for Social Marketing and Health; St George's University of London Population Health Research Institute<br>Ford, Allison; University of Stirling Institute for Social Marketing and Health<br>Eadie, Douglas; University of Stirling Institute for Social Marketing and Health<br>MacGregor, Andy; NatCen Social Research<br>Hunt, Kate; University of Stirling Institute for Social Marketing and Health<br>MacKintosh, Anne Marie; University of Stirling Institute for Social Marketing and Health
**Primary Subject Heading**:	Public health
Secondary Subject Heading:	Immunology (including allergy)
Keywords:	COVID-19, IMMUNOLOGY, Epidemiology < INFECTIOUS DISEASES, Public health < INFECTIOUS DISEASES, PUBLIC HEALTH

SCHOLARONE™
Manuscripts

I, the Submitting Author has the right to grant and does grant on behalf of all authors of the Work (as defined in the below author licence), an exclusive licence and/or a non-exclusive licence for contributions from authors who are: i) UK Crown employees; ii) where BMJ has agreed a CC-BY licence shall apply, and/or iii) in accordance with the terms applicable for US Federal Government officers or employees acting as part of their official duties; on a worldwide, perpetual, irrevocable, royalty-free basis to BMJ Publishing Group Ltd ("BMJ") its licensees and where the relevant Journal is co-owned by BMJ to the co-owners of the Journal, to publish the Work in this journal and any other BMJ products and to exploit all rights, as set out in our licence.

The Submitting Author accepts and understands that any supply made under these terms is made by BMJ to the Submitting Author unless you are acting as an employee on behalf of your employer or a postgraduate student of an affiliated institution which is paying any applicable article publishing charge ("APC") for Open Access articles. Where the Submitting Author wishes to make the Work available on an Open Access basis (and intends to pay the relevant APC), the terms of reuse of such Open Access shall be governed by a Creative Commons licence – details of these licences and which Creative Commons licence will apply to this Work are set out in our licence referred to above.

Other than as permitted in any relevant BMJ Author's Self Archiving Policies, I confirm this Work has not been accepted for publication elsewhere, is not being considered for publication elsewhere and does not duplicate material already published. I confirm all authors consent to publication of this Work and authorise the granting of this licence.

For peer review only

A national survey of attitudes towards and intentions to vaccinate against COVID-19: implications for communications

Martine Stead, Deputy Director[1*]
Curtis Jessop, Research Director for Longitudinal Surveys[2]
Kathryn Angus, Research Officer[1]
Helen Bedford, Professor of Children's Health[3]
Michael Ussher, Professor of Behavioural Medicine[1,4]
Allison Ford, Research Fellow[1]
Douglas Eadie, Research Fellow[1]
Andy MacGregor, Director of Policy Research[2]
Kate Hunt, Professor of Behavioural Sciences and Health[1]
Anne Marie MacKintosh, Senior Researcher[1]

[1]Institute for Social Marketing and Health, University of Stirling, Stirling, UK
[2]NatCen The National Centre for Social Research, London, UK
[3]Great Ormond Street Institute of Child Health, University College London, London, UK
[4]Population Health Research Institute, St George's University of London, London, UK

*Corresponding author: martine.stead@stir.ac.uk, +44(0)1786 467390, Institute for Social Marketing and Health, University of Stirling, Stirling, FK9 4LA, UK

For peer review only

For peer review only - http://bmjopen.bmj.com/site/about/guidelines.xhtml

Abstract

Objectives

To examine public views on COVID-19 vaccination and consider the implications for communications and targeted support.

Design

Cross-sectional study.

Setting

Online and telephone nationally representative survey in Great Britain, January to February 2021.

Participants

4,978 adults. Survey response rate was 84%, among the 5,931 panellists invited.

Main Outcome Measures

Sociodemographic characteristics (age, gender, ethnicity, education, financial status), COVID-19 status, vaccine acceptance, trust in COVID-19 vaccination information sources, perceptions of vaccination priority groups, and perceptions of importance of second dose.

Results

COVID-19 vaccine acceptance (83%) was associated with increasing age, higher level of education and having been invited for vaccination. Acceptance decreased with unconfirmed past COVID-19, greater financial hardship, and non-White British ethnicity; Black/Black British participants had lowest acceptance. Overall, healthcare and scientific sources of information were most trusted. Compared with White British participants, other ethnicities had lower trust in healthcare and scientific sources. Those with lower educational attainment or financial hardship had lower trust in healthcare and scientific sources. Those with no qualifications had higher trust in media and family/friends. While trust was low overall in community or faith leaders it was higher among those with Asian/Asian British and Black/Black British ethnicity compared with White British participants. Views of vaccine prioritisation were mostly consistent with UK official policy but there was support for prioritising additional groups. There was high support for having the second vaccine dose.

Conclusions

Targeted engagement is needed to address COVID-19 vaccine hesitancy in non-White British ethnic groups, in younger adults, and among those with lower education, greater financial hardship and unconfirmed past infection. Healthcare professionals and scientific advisors should play a central role in communications and tailored messaging is needed for hesitant groups. Careful communication around vaccination prioritisation continues to be required.

For peer review only

STRENGTHS AND LIMITATIONS OF THIS STUDY

- The survey was conducted at the start of vaccine rollout giving timely insight into COVID-19 vaccine acceptance/hesitancy and trusted information sources when individuals' decision-making was real rather than hypothetical.
- Results come from a large probability-based sample, representative of adults in Great Britain, which was sufficiently large to examine ethnicity in detail.
- The survey did not include those who are institutionalised (e.g., prisoners), notably difficult to reach populations (e.g., homeless) or those not speaking English (therefore, our ethnic minority sample may underrepresent certain views).
- The survey benefited from a rigorous design, with questionnaire development informed by cognitive interviews conducted with a broad range of individuals.
- A cross-sectional survey cannot infer causality; although variables likely to be important in vaccine acceptance were included, the results are exploratory.

For peer review only

Introduction

Widespread vaccination is likely to be one of the most effective ways of controlling the COVID-19 pandemic, and is central to the UK government's recovery strategy. The UK vaccine programme began in December 2020, prioritising older adults in care homes and their carers, those aged over 80, and frontline health and social-care workers.[1] Administration of first doses of vaccination to the adult population, by decade of age, is to be completed by July 2021. Uncertainty or unwillingness to accept vaccination – 'vaccine hesitancy'[2] – threatens comprehensive vaccination.[3,4] Before the introduction of a COVID-19 vaccine, UK surveys reported that 64% to 82% of adults were willing to be vaccinated.[5-12] Most of these studies used non-probability samples, introducing selection bias and limiting generalisability. Increased vaccine confidence has been reported since vaccination commenced;[13] possibly due to increased COVID-19 cases and deaths, a further UK lockdown in early 2021, and, increasingly, vaccination becoming the social norm. It is important to examine vaccine acceptance when people are making *active,* rather than *hypothetical,* decisions about vaccination. This also provides insight into potential acceptance of repeat COVID-19 vaccination and boosters.[14]

UK uptake has been high (94% of adults surveyed in April reported uptake or intention to accept vaccination),[13] but there remain concerns about uptake in subpopulations, such as younger adults and some ethnic minorities,[15] giving rise to initiatives such as social media campaigns featuring non-White celebrities.[16] Robust, timely data are needed to identify the characteristics of groups with lower acceptance and the information sources they trust, to inform targeted interventions. It is also important to assess whether attitudes towards COVID-19 vaccination have been affected by specific events and media coverage. Two issues in the UK merit particular attention. First, the government followed recommendations to offer the vaccine to priority groups.[1] If this approach is continued, it is important to examine its acceptability and any implications for communications. Secondly, the government decided, on 30th December 2020, to deviate from recommended protocols for the Pfizer-BioNTech vaccine by extending the interval between doses to up to 12 weeks;[1] this precipitated concerns that it may lead to reduced willingness to be vaccinated or to have a second dose.[17]

We conducted a survey in early 2021, using probability sampling, to examine public views on COVID-19 vaccination and consider the implications for communications. During this period most people aged over 80 had been invited to have a vaccine and invitations were being extended to those aged over 70, with other age groups advised they would be invited in the coming months.

Methods

For peer review only - http://bmjopen.bmj.com/site/about/guidelines.xhtml

We administered a cross-sectional survey with adults (aged 18+) in Great Britain (GB) in January and February 2021. This paper follows the STROBE Statement for reporting cross-sectional studies.[18]

Questionnaire development and testing

The questionnaire was informed by a review of studies on public attitudes towards and experiences of vaccines and COVID-19. Existing measures were adapted[5,19,20] and new questions developed. The questionnaire was cognitively tested with members of the public to ensure understandability.[21] Interviews were conducted with 20 individuals recruited by an external fieldwork agency. A purposive sampling approach was employed, with quotas used to ensure people with a mix of genders, ages, parental status, likelihood of accepting a COVID-19 vaccination, and experiences of shielding were recruited. The questionnaire was subsequently revised based on these interviews. Final revisions reflected changes in the UK's vaccine rollout. The questionnaire covered: vaccine acceptance, trust in vaccine information sources, perception of priority groups, COVID-19 status, and perceived importance of a second dose. The questionnaire is provided in Supplementary Material, Methods S1.

Sample and data collection

The target population for the study was adults (18+) living in Great Britain. The survey was administered to the probability-based NatCen Panel,[22] recruited from the 2018, 2019, and 2020 waves of the British Social Attitudes survey (BSA), with participants randomly selected from England, Wales and Scotland. All BSA respondents who agreed to join the Panel, had not requested to leave or become inactive were invited to take part, maintaining the random probability design. Data were collected through online and telephone interviews (conducted 14th January to 7th February 2021). Panellists were sent reminders and offered a small financial sum (£5 - £20 depending on interview duration and whether participant had characteristics which are typically under-represented in survey samples) in recognition of their contribution. Participants who did not initially take part online, and for whom a telephone number was available, were followed up by a telephone interviewer and encouraged to take part online or given the opportunity to take part on the telephone. Among 5,931 panellists invited, the survey response rate was 84%, with 4,978 completing it (4,776 online, 202 by telephone). Supplementary Material, Table S1 details overall response rate, accounting for non-response at the panel recruitment stage and panel attrition. Data were weighted for non-response and to be representative of the GB adult population (see Supplementary Material, Methods S2).

Measures

Sociodemographic and other characteristics

Data on age, gender, ethnicity, education, country, urban/rural status, and financial status were obtained from existing information on NatCen panellists. Full details of sub-groups of each variable are provided in Tables 1 and 2. Age was categorised into bands from 18-29 years then ten-year

For peer review only - http://bmjopen.bmj.com/site/about/guidelines.xhtml

bands up to 80+. Self-assigned ethnicity was recorded in six categories, and education in five categories according to highest qualification. As indices of multiple deprivation were not available, self-reported financial status was used. COVID-19 status was derived from two items: 1) *"Have you officially been diagnosed with the coronavirus (COVID-19)?" (yes/no/don't know)*; those answering other than *'yes'* were asked: 2) *"Do you think you have ever had the coronavirus (COVID-19)?" (yes-definitely/yes-probably/no-probably not/no-definitely not/don't know)*.

Vaccine measures

Vaccine acceptance was derived from five items: 1) "*Have you been offered a vaccine for COVID-19?*" (*yes/no*). Those answering '*yes*' were asked: 2) "*And have you had that vaccine?*" (*yes/no*). Participants who had been offered but not yet had the vaccine were then asked: 3) "*And do you intend to have that vaccine?*" (*yes/no/not sure*). Participants who had not yet been offered the vaccine were asked: 4) "*Would you accept the vaccine for yourself if it is offered to you?*" (*yes/no/not sure*). Those answering '*not sure*' were asked: 5) "*If you had to choose, if a COVID-19 vaccine became publicly available and you were offered it, would you accept the vaccine for yourself?*" (*yes/no/I'm really not sure*). Participants were classed as: 'Accepted/accepting' if they answered '*yes*' to any of items 2, 3, 4, or 5; 'Uncertain' if they answered '*not sure*' to item 3 or '*I'm really not sure*' to item 5; and 'Refused/refusing' if they answered '*no*' to items 3, 4, or 5.

Trust in information sources was assessed for 13 sources: "*To what extent, if at all, would you trust information about a COVID-19 vaccine from each of the following sources?*" (see Table 3): *completely* (1); *a great deal* (2); *somewhat* (3); *very little* (4); *not at all* (5).

Perceptions of vaccine priority groups were assessed across 11 groups (see Table 4): "*Below are some groups that some people say should be the first to be offered a COVID-19 vaccine. For each one, how high a priority do you think it is that they get a COVID-19 vaccine, or do you not think they should be offered the vaccine at all?*": 1 '*One of the first*', 5 '*One of the last*', with an additional option "*They should not be offered a vaccine*".

Perceived importance of receiving the second dose of the vaccine was assessed with: "*How important, if at all, do you think it is for people to get the second injection of the COVID-19 vaccine?*": *very important* (1); *fairly important* (2); *not very important* (3); *not at all important* (4).

Data analysis

Descriptive data, including bivariate analyses, were weighted to be representative of British adult population. Initial bivariate analyses, using chi-square tests, examined correlates of vaccine acceptance and trust in sources of information about COVID-19 vaccination. Multivariate logistic regression was conducted to examine differences in vaccine acceptance controlling for socio-demographic variables, vaccine offer, and COVID-19 status. The dependent variable dichotomised those classed as *accepted/intending to accept* vs *uncertain/refused/intend to refuse*. Age was entered as a categorical variable and the 'difference' contrast within SPSS logistic regression was used to test influence of each increasing age group, relative to younger ages (e.g., 30-39 vs 18-29;

For peer review only - http://bmjopen.bmj.com/site/about/guidelines.xhtml

80+ vs 18-79) (see Table 2). Sociodemographic variation in trust in information sources was examined using multivariate logistic regressions. For each information source, the dependent variable dichotomised the 5-point scale into trusting *completely or a great deal* vs *somewhat/very little/not at all*. Cases were excluded from the logistic regressions if they had missing data on the dependent or any independent variables. All logistic regressions were conducted on unweighted data as sociodemographic variables were included as control variables. For each information source, logistic regression analysis examined likelihood of trust (completely/a great deal v somewhat/very little/not at all) by sociodemographic characteristics (Supplementary Material, Tables S2-S14). Given the large sample size in this study, the threshold for statistical significance was set at $p<0.01$. Data were analysed using SPSS v27.

Public and patient involvement

The questionnaire was cognitively tested by members of the public to ensure understandability (see 'Questionnaire development and testing' above).

Results

Sample characteristics

The weighted sample comprised adults aged 18 and over (see Table 1). Over half (52%) were female and 81% were White British. Around two-thirds reported 'living comfortably'/'doing alright', while one in ten rated their financial status as 'quite' or 'very difficult'. Just over two-fifths were educated to degree level or above, while for almost a quarter their highest qualification was A level or equivalent. A minority (12%) had no qualifications. A minority indicated having been diagnosed with COVID-19 (6%); nearly two-thirds thought they probably or definitely had not had COVID-19; 11% were unsure.

Vaccine offer and acceptance

At the time of the survey, 14% (n=716) had been offered the vaccine. Of these, 92% (n=658) had accepted or intended to, 4% (n=29) were uncertain, and 4% (n=29) had refused or intended to refuse.

Among those not yet offered the vaccine, 82% (n=3479) intended to accept, while 11% (n=471) were uncertain and 7% (n=311) indicated they would refuse. Overall, the acceptance level was 83% (n=4137), with 10% (n=502) uncertain and 7% (n=340) refusing.

Multivariate logistic regression, with vaccine acceptance as the outcome variable (accepted/accepting v refused/refusing/uncertain), indicated likelihood of acceptance increased with age (Table 2). For example, those aged 40-49 were more likely than 18-39-year-olds to indicate acceptance (AOR=1.43, 95%CI (1.12, 1.83, p=0.004) as were 70-79-year-olds compared with 18-69-year-olds (AOR=3.31, 95%CI (2.22, 4.95), $p<0.001$). Acceptance was also positively associated with education. Those with at least a degree were three times as likely to indicate acceptance

For peer review only - http://bmjopen.bmj.com/site/about/guidelines.xhtml

(AOR=3.03, 95%CI (2.17, 4.23), p<0.001) and those educated to A level or equivalent nearly twice as likely (AOR=1.80, 95%CI (1.27, 2.55), p<0.001), compared with people without qualifications. Lower acceptance was also associated with financial hardship and ethnicity. For example, compared with those 'living comfortably', people 'finding it very difficult' were much less likely to accept the vaccine (AOR=0.35, 95%CI (0.22, 0.55), p<0.001). Compared with White British participants, those from other ethnic groups were less likely to accept the vaccine. Black/Black British participants had the lowest likelihood of accepting (AOR=0.25, 95%CI (0.14, 0.43), p<0.001). This is illustrated in the descriptive data too, with 87% of White British participants indicating vaccine acceptance compared with 58% among Black/Black British, 61% among mixed/multiple ethnic groups and 61% among Asian/Asian British.

After controlling for demographic variables, vaccine acceptance was positively associated with having been invited for vaccination (AOR=1.73, 95%CI (1.24, 2.43), p=0.001), but negatively associated with COVID-19 status. Compared with those who had 'probably not' or 'definitely not' had COVID-19, those who thought they had 'definitely' or 'probably' had COVID-19 were less likely to indicate acceptance (AOR = 0.40, 95%CI (0.26, 0.60), p<0.001 and AOR=0.71, 95%CI (0.56, 0.91), p=0.006 respectively). Confirmed diagnosis with COVID-19 was not significantly associated with vaccine acceptance, after controlling for demographic variables.

Trust in information sources

The three most trusted information sources were: the NHS; doctors/nurses/other healthcare professionals; and scientific and medical advisers. These groups were trusted 'completely/a great deal' by around 80% of participants (Table 3). Only 44% trusted the UK government 'completely/a great deal'. The three least trusted sources were celebrities and social media influencers, social media, and faith or community leaders; around two-thirds indicated they would have no trust in each. A majority (61%) indicated they had very little/no trust in the media (e.g., newspapers/magazines/television/radio).

Trust did not differ by gender except for drug companies and the WHO, with females more likely to indicate trust in these sources (Tables S5 and S9 respectively).

Trust was higher among older participants for five sources (doctors/nurses/other healthcare professionals, NHS, UK government, media, and family/friends; Tables S2, S4, S6, S10, S13). For example, trust in the UK government was higher among those aged 50-59 than 18-49-year-olds (Table S6).

Trust varied by education. Compared with those without qualifications, other participants were more likely to trust five sources (doctors/nurses/other healthcare professionals, NHS, scientists, WHO; Tables S2, S4, S8, S9) and less likely to trust another five (drug companies, media, social media, celebrities/social media influencers, family/friends; Tables S5, S10-S13). Compared with those 'living comfortably' participants in more difficult financial situations were less likely to trust the seven sources most closely aligned with scientific or clinical expertise (doctors/nurses/other healthcare

For peer review only - http://bmjopen.bmj.com/site/about/guidelines.xhtml

professionals, pharmacists, NHS, drug companies, UK government, scientists, WHO; Tables S2-S6, S8, S9). Similarly, participants from minority ethnic groups were less likely to trust scientific or clinical sources than White British participants (Tables S2-S4, S8, S9). Whilst lack of trust in faith or community leaders was low overall, Asian/Asian British participants were more likely than White British to trust faith/community leaders (AOR=4.82, 95%CI (2.76, 8.42), p<0.001) as were Black/Black British participants (AOR=4.52, 95%CI (2.04, 9.99), p<0.001) (Table S14).

Views on prioritisation

Nine in ten participants rated healthcare professionals as highest priority for vaccination. Over 70% indicated those with serious health conditions/heightened vulnerability to COVID-19, care home workers and residents, and over 80s should be 'one of the first' to be vaccinated (Table 4). Priority was also given to social care workers, schoolteachers, and those directly working with the public. Over a third considered each of these groups should be 'one of the first' to be vaccinated, and 70% or more rated them in the top two priority levels. People aged under 18 were rated as lowest priority, and 6% considered the vaccine should not be offered to this group.

Importance of second dose

Nearly all participants (96%, n=4,761) considered it 'very' or 'fairly important' to receive the second vaccine dose. This increased to 99% (n=4,096) amongst those who intended to accept the vaccine.

Discussion

Principal findings

Overall, acceptance was high, with 83% having received or intending to have the vaccine. Acceptance increased with age and education, and if invited for vaccination. It decreased with financial hardship, and among non-White British ethnicities and those with unconfirmed past COVID-19. Clinical and scientific information was most trusted, with sociodemographic differences for different sources. Policy on a second dose and vaccination priority groups[1] was supported.

Comparison with other studies

We confirmed lower acceptance in younger groups;[6-8,10,11] acceptance was higher if invited for vaccination, a finding observed for other vaccines in other populations ,[23] and emphasising the importance of ensuring vaccine invitations are issued, using appropriate language with translations if necessary. Confirmation of lower acceptance in non-White British ethnicities.[5,6,9,24] is concerning given increased risk of infection and poorer outcomes.[25] This lower acceptance has been reported to result from an erosion of trust with health care services as a consequence of past experiences of unethical experimental research conducted among black populations, the lack of participants from ethnic minorities included in health research, particularly vaccine trials, and poor experiences of healthcare.[15] Successful initiatives by primary care health professionals to overcome these barriers have been reported, but they require considerable resources.[26] We confirmed lower acceptance in

For peer review only - http://bmjopen.bmj.com/site/about/guidelines.xhtml

those with lower educational attainment and greater financial hardship,[6,8-10,12,27] leaving these groups at risk of infection and increasing likelihood of emergence of variants.[28] Gender was not associated with vaccine hesitancy in the analysis reported in this paper, but female gender has been found to be a factor associated with greater COVID-19 vaccine hesitancy in some other studies;[6,8-10,29] further research is needed to explore whether and why gender may relate to hesitancy.

A novel finding was that there was lower vaccine acceptance among those with unconfirmed but suspected COVID-19. This suggests that prior infection is thought to confer immunity, or that recovery fosters a perception of decreased severity, but further research is needed to explore this relationship. However, past infection does not guarantee protection and people may still be infectious.[30,31] Messaging should target those with prior infection.

There are other implications for communications. While high acceptance suggests communications are effective, identifying barriers in hesitant groups is a priority for developing interventions.[3,15,19,32] Trusted information sources are needed. The most trusted were the NHS, healthcare professionals, and scientific and medical advisers. This suggests that healthcare professionals have a central role in promoting vaccination in initiatives and during consultations. That government and media are less trusted has implications for acceptance.[7,8,27,33] We found particularly low levels of trust in social media and celebrities. However, this does not necessarily mean that they do not influence feelings about vaccination, and, with careful research, they could still play a positive role in communications (for example, initiatives using ethnic minority celebrities and opinion leaders.[16]) Such initiatives would need to use pre-testing of messages to ensure they are appropriately tailored to target audiences, while avoiding stereotyping, and would require evaluation of acceptability and effectiveness.

Differences in trust varied by socio-demographics. Compared with White British participants, other ethnicities had lower trust in healthcare and scientific sources. Although trust in faith/community leaders was low, it was higher in Asian and Black British participants, suggesting a role for these leaders.[15] Those with lower educational attainment or financial hardship had lower trust in healthcare and scientific sources. Those with no qualifications had higher trust in media and family/friends. This suggests a need for a mix of sources for these groups. Mainstream media may have a role to play, despite lower trust.[27]

Reassuringly for further campaigns, for the first time, this study reported that prioritisation was considered acceptable by the general public and there was support for additional prioritisation of schoolteachers and others in direct contact with the public. This is consistent with research suggesting that healthcare workers themselves support the decision to prioritise vaccination for frontline health and social care workers and those at increased risk of vulnerability to infection.[34]As planning begins for further vaccination, careful communication regarding prioritisation should continue. We found high support for a second dose, suggesting the UK's decision to extend the

For peer review only - http://bmjopen.bmj.com/site/about/guidelines.xhtml

period between doses has not dented public confidence. While the high acceptance rate may suggest that acceptance will be similarly high in future COVID-19 vaccination programmes, this cannot be assumed. The survey was conducted during a period of considerable public anxiety, with rising infection rates and restrictions on many activities including travel. Similar acceptance rates may not be observed in future if the threat is perceived to have receded and society is functioning more normally.

Strengths and limitations

Strengths include the large probability-based nationally representative sample, ability to analyse by ethnicity and surveying during vaccine roll-out. Our findings can be generalised to GB's adult population, however global contexts for COVID-19 and vaccination vary. Although not generalisable to them, the findings are still informative for other countries. The study has limitations. As it is cross-sectional, we cannot infer causality; although we included variables likely to be important in vaccine acceptance, these results are exploratory. Our qualitative studies will deepen understanding of associations. A survey repeated when COVID-19 cases and deaths are low, and without lockdown, might yield different responses. We did not survey individuals who are institutionalised (e.g., prisoners), notably difficult to reach (e.g., homeless), or those not speaking English (therefore, our ethnic minority sample may underrepresent certain views); specific surveys are needed for these groups. We investigated vaccination intention. Actual uptake may be lower, although it is likely that factors associated with intention will influence uptake.

Conclusions

COVID-19 vaccination acceptance is high in GB. Targeted engagement is needed to address hesitancy in non-White British ethnic groups, those with lower education, those younger, those with greater financial hardship and those with unconfirmed but suspected past infection. Healthcare professionals and scientific advisors should lead communications and tailoring is needed. Work is needed to rebuild trust in government information. There is high support for having the second vaccine dose. Views of vaccine prioritisation are mostly consistent with UK official policy but there was support for prioritising additional groups and careful communication around vaccination prioritisation should continue.

For peer review only

Contributors:

MS, CJ, HEB, KH, and AMM conceived the study, supported by AF, DE, and AM. MS, CJ, KA, HB, and AMM designed the questionnaire, supported by MU, AF, DE, AM, and KH. CJ and AMM acquired and analysed the data, which was interpreted by MS, CJ, HB, MU, KH and AMM. MS and AMM drafted the manuscript supported by CJ, HB, MU, and KH. KA, HB, MU, and KH critically revised the article, supported by MS, CJ, AF, DE, AM, and AMM. All authors read the final version of the manuscript and gave approval for it to be published. AMM, CJ, and MS had access to the data in the study and take responsibility for the integrity of the data and the accuracy of the data analysis. The corresponding author attests that all listed authors meet authorship criteria and that no others meeting the criteria have been omitted. MS is the guarantor.

Ethics approval:

The study received ethical approval from NatCen's Research Ethics Committee (ID P14307). Participants gave informed consent before taking part.

Transparency declaration:

The lead author and manuscript's guarantor (MS) affirms that the manuscript is an honest, accurate, and transparent account of the study being reported; that no important aspects of the study have been omitted; and that any discrepancies from the study as originally planned have been explained.

Role of the funding source:

The OPTIMising general public Uptake of a COVID-19 vaccine (OPTIMUM) study was supported by a UK Research & Innovation (UKRI) Ideas to Address COVID-19 award (no. ES/V012851/1). The funder had no role in the study design; in the collection, analysis, and interpretation of data; in the writing of the report; and in the decision to submit the article for publication. All researchers were independent from the funder and all authors had full access to all of the data (including statistical reports and tables) in the study and can take responsibility for the integrity of the data and the accuracy of the data analysis.

Dissemination to participants and related patient and public communities:

We do not plan to disseminate study results to individual study participants, however findings from the OPTIMUM study will be shared with the public via press releases, social media and interviews, and lay-audience briefing papers.

Data sharing:

After completion of the study, the survey dataset will be deposited in the UK Data Archive.

Competing interests:

All authors have completed the ICMJE uniform disclosure form at www.icmje.org/coi_disclosure.pdf and declare: no support from any organisation for the submitted work; KH has received another UK Research and Innovation (Economic and Social Research Council) grant on the impact of COVID-19; no other relationships or activities that could appear to have influenced the submitted work.

For peer review only - http://bmjopen.bmj.com/site/about/guidelines.xhtml

Acknowledgments:
We thank the questionnaire development and testing and survey and data delivery teams at NatCen for their work on the survey, and Professor Mark Petticrew at the London School of Hygiene & Tropical Medicine for acting as adviser to the study.

For peer review only

For peer review only - http://bmjopen.bmj.com/site/about/guidelines.xhtml

References

1 UK Government Department of Health and Social Care. Joint Committee on Vaccination and Immunisation: advice on priority groups for COVID-19 vaccination, 30 December 2020. Jan 6, 2021. https://www.gov.uk/government/publications/priority-groups-for-coronavirus-covid-19-vaccination-advice-from-the-jcvi-30-december-2020/joint-committee-on-vaccination-and-immunisation-advice-on-priority-groups-for-covid-19-vaccination-30-december-2020.

2 MacDonald NE, SAGE Working Group on Vaccine Hesitancy. Vaccine hesitancy: definition, scope and determinants. *Vaccine* 2015;33:4161-4.

3 Larson HJ, Jarrett C, Eckersberger E, Smith DMD, Paterson P. Understanding vaccine hesitancy around vaccines and vaccination from a global perspective: a systematic review of published literature, 2007-2012. *Vaccine* 2014;32:2150-9.

4 Lane S, MacDonald NE, Marti M, Dumolard L. Vaccine hesitancy around the globe: analysis of three years of WHO/UNICEF Joint Reporting Form data—2015–2017. *Vaccine* 2018;36:3861-7.

5 Bell S, Clarke R, Mounier-Jack S, Walker JL, Paterson P. Parents' and guardians' views on the acceptability of a future COVID-19 vaccine: A multi-methods study in England. *Vaccine* 2020;38:7789-98.

6 Freeman D, Loe BS, Chadwick A, Vaccari C, Waite F, Rosebrock L, et al. COVID-19 vaccine hesitancy in the UK: the Oxford coronavirus explanations, attitudes, and narratives survey (Oceans) II. *Psychol Med* 2020; published online Dec 11. https://dx.doi.org/10.1017/S0033291720005188.

7 Lazarus JV, Ratzan SC, Palayew A, Gostin LO, Larson HJ, Rabin K, et al. A global survey of potential acceptance of a COVID-19 vaccine. *Nat Med* 2021;27:225-8. https://doi.org/10.1038/s41591-020-1124-9.

8 Murphy J, Vallières F, Bentall RP, Shevlin M, McBride O, Hartman TK, et al. Psychological characteristics associated with COVID-19 vaccine hesitancy and resistance in Ireland and the United Kingdom. *Nat Commun* 2021;12:29.

9 Robertson E, Reeve KS, Niedzwiedz CL, Moore J, Blake M, Green M, et al. Predictors of COVID-19 vaccine hesitancy in the UK household longitudinal study. *Brain Behav Immun* 2021;94:41-50.

10 Paul E, Steptoe A, Fancourt D. Attitudes towards vaccines and intention to vaccinate against COVID-19: Implications for public health communications. *Lancet Reg Health Eur* 2021;1:100012. https://doi.org/10.1016/j.lanepe.2020.100012

11 Sherman SM, Smith LE, Sim J, Amlôt R, Cutts M, Dasch H, et al. COVID-19 vaccination intention in the UK: results from the COVID-19 Vaccination Acceptability Study (CoVAccS), a nationally representative cross-sectional survey. *Hum Vaccin Immunother* 2020;26:1-10.

12 Williams L, Flowers P, McLeod J, Young D, Rollins L. The Catalyst Project Team. Social patterning and stability of intention to accept a COVID-19 vaccine in Scotland: will those most at risk accept a vaccine? *Vaccines (Basel)* 2021;9:17.

For peer review only - http://bmjopen.bmj.com/site/about/guidelines.xhtml

13 Office for National Statistics. Coronavirus (COVID-19) latest insights. Apr 16, 2021. https://www.ons.gov.uk/peoplepopulationandcommunity/healthandsocialcare/conditionsanddiseases/articles/coronaviruscovid19/latestinsights#vaccinations. [Accessed Apr 19 2021].

14 Mahase E. Covid-19: Booster vaccine to be rolled out in autumn as UK secures 60m more Pfizer doses. *BMJ* 2021;373:n1116.

15 Razai MS, Chaudhry UAR, Doerholt K, Bauld L, Majeed A. Covid-19 vaccination hesitancy. *BMJ* 2021;373:n1138.

16 Mohdin A. BAME groups urged to have Covid vaccine in UK TV ad campaign. *The Guardian*, Feb 18, 2021. https://www.theguardian.com/world/2021/feb/18/bame-groups-urged-to-have-covid-vaccine-in-uk-tv-ad-campaign.

17 Sewell HF, Robertson JFR, Stewart M, Kendrick D, Bird SM. Revisiting the UK's strategy for delaying the second dose of the Pfizer covid-19 vaccine. Jan 20, 2021. https://blogs.bmj.com/bmj/2021/01/20/revisiting-the-uks-strategy-for-delaying-the-second-dose-of-the-pfizer-covid-19-vaccine/.

18 von Elm E, Altman DG, Egger M, Pocock SJ, Gøtzsche PC, Vandenbroucke JP. Strengthening the Reporting of Observational Studies in Epidemiology (STROBE) statement: guidelines for reporting observational studies. *BMJ*. 2007;335:806-808.

19 World Health Organization Regional Office for Europe. Survey Tool and Guidance. Rapid, simple, flexible behavioural insights on COVID-19. Copenhagen: WHO Regional Office for Europe; 2020. https://www.euro.who.int/__data/assets/pdf_file/0007/436705/COVID-19-survey-tool-and-guidance.pdf?ua=1.

20 Gallup. Ch 5 Attitudes to vaccines. In: Gallup. Wellcome Global Monitor. How does the world feel about science and health? London: Wellcome Trust, 2019: 104-25. https://cms.wellcome.org/sites/default/files/wellcome-global-monitor-2018.pdf.

21 Collins D. Cognitive Interviewing Practice. London: SAGE Publications Ltd, 2014.

22 Jessop C. The NatCen Panel: developing an open probability-based mixed-mode panel in Great Britain. *Soc Res Pract* 2018;6:2-14. https://the-sra.org.uk/Common/Uploaded%20files/Social%20Research%20Practice%20Journal/social-research-practice-journal-issue-06-summer-2018.pdf.

23 Jacobson Vann JC, Jacobson RM, Coyne-Beasley T, Asafu-Adjei JK, Szilagyi PG. Patient reminder and recall interventions to improve immunization rates. *Cochrane Database Syst Rev* 2018;1:CD003941.

24 Thorneloe R, Wilcockson H, Lamb M, Jordan CH, Arden M. Willingness to receive a COVID-19 vaccine among adults at high-risk of COVID-19: a UK-wide survey. *PsyArXiv* 2020; published online Jul 20. https://psyarxiv.com/fs9wk (preprint).

25 Mathur R, Rentsch CT, Morton CE, Hulme WJ, Schultze A, MacKenna B, et al. Ethnic differences in SARS-CoV-2 infection and COVID-19-related hospitalisation, intensive care unit

For peer review only - http://bmjopen.bmj.com/site/about/guidelines.xhtml

For peer review only

admission, and death in 17 million adults in England: an observational cohort study using the OpenSAFELY platform. *Lancet*. 2021;397:1711-24.

26 Mahase E. Covid-19 vaccines: GPs boost uptake by calling patients and teaming up with community groups *BMJ* 2021;374:n2093.

27 Allington D, McAndrew S, Moxham-Hall V, Duffy B. Coronavirus conspiracy suspicions, general vaccine attitudes, trust and coronavirus information source as predictors of vaccine hesitancy among UK residents during the COVID-19 pandemic. *Psychol Med* 2021; published online April 12. https://doi.org/10.1017/s0033291721001434

28 Sah P, Vilches TN, Moghadas SM, Fitzpatrick MC, Singer BH, Hotez PJ, et al. Accelerated vaccine rollout is imperative to mitigate highly transmissible COVID-19 variants. *EClinicalMedicine* 2021;35:100865.

29 Malik AA, McFadden SM, Elharake J, Omer SB. Determinants of COVID-19 vaccine acceptance in the US. *EClinicalMedicine*. 2020;26:100495. https://doi.org/10.1016/j.eclinm.2020.100495

30 Hansen CH, Michlmayr D, Gubbels SM, Mølbak K, Ethelberg S. Assessment of protection against reinfection with SARS-CoV-2 among 4 million PCR-tested individuals in Denmark in 2020: a population-level observational study. *Lancet* 2021;397:1204-12.

31 Hall VJ, Foulkes S, Charlett A, Atti A, Monk EJM, Simmons R, et al. SARS-CoV-2 infection rates of antibody-positive compared with antibody-negative health-care workers in England: a large, multicentre, prospective cohort study (SIREN). *Lancet* 2021;397:1459-69.

32 Lockyer B, Islam S, Rahman A, Dickerson J, Pickett K, Sheldon T, et al. Understanding COVID-19 misinformation and vaccine hesitancy in context: Findings from a qualitative study involving citizens in Bradford, UK. *Health Expect* 2021; published online May 4. https://dx.doi.org/10.1111/hex.13240

33 Williams L, Gallant AJ, Rasmussen S, Brown Nicholls LA, Cogan N, et al. Towards intervention development to increase the uptake of COVID-19 vaccination among those at high risk: Outlining evidence-based and theoretically informed future intervention content. *Br J Health Psychol* 2020;25:1039-54.

34 Manby R, Dowrick A, Karia A, Maio L, Buck C, Singleton G, et al. Healthcare workers' perceptions and attitudes towards the UK's COVID-19 vaccination programme. *medRxiv* 2021; published online Mar 31. https://doi.org/10.1101/2021.03.30.21254459 (preprint).

For peer review only

For peer review only - http://bmjopen.bmj.com/site/about/guidelines.xhtml

Table 1 Sample characteristics

	Unweighted n	Unweighted %	Weighted n	Weighted %
**Age**				
18-29	464	9.4	824	16.7
30-39	772	15.6	852	17.3
40-49	848	17.1	806	16.3
50-59	904	18.3	867	17.6
60-69	1011	20.4	711	14.4
70-79	773	15.6	657	13.3
80+	178	3.6	218	4.4
**Gender**				
Male	2136	42.9	2402	48.3
Female	2830	56.9	2567	51.6
Other	10	0.2	7	0.1
**Ethnicity**				
White British	4261	86.3	3999	81.2
Any other White background	319	6.5	335	6.8
Mixed or multiple ethnic groups	64	1.3	100	2.0
Asian or Asian British	164	3.3	306	6.2
Black or Black British	67	1.4	101	2.1
Other	62	1.3	81	1.6
**Country**				
England	4369	87.9	4291	86.3
Scotland	390	7.8	442	8.9
Wales	212	4.3	237	4.8
**Urban/rural status**[#]				
Urban	3789	76.2	4006	80.6
Rural	1182	23.8	965	19.4
**Highest educational qualification**				
Degree or equivalent, and above	2503	50.4	2077	41.8
A levels or vocational level 3 or equivalent and above, but below degree	1005	20.2	1131	22.8
Other qualifications below A levels or vocational level 3 or equivalent	788	15.9	838	16.9
Other qualification	256	5.2	304	6.1
No qualifications	416	8.4	618	12.4
**Subjective Financial Status**				
Living comfortably	1552	31.2	1289	26.0
Doing alright	2028	40.8	2035	40.9
Just about getting by	975	19.6	1132	22.8
Finding it quite difficult	271	5.5	337	6.8
Finding it very difficult	142	2.9	175	3.5
**COVID-19 Status**				
Diagnosed with COVID-19	241	4.8	294	5.9
Think definitely had COVID-19	140	2.8	172	3.5
Think probably had COVID-19	710	14.3	755	15.2

For peer review only

For peer review only - http://bmjopen.bmj.com/site/about/guidelines.xhtml

Think probably not had COVID-19	1945	39.1	1880	37.8
Think definitely not had COVID-19	1393	28.0	1305	26.2
Don't know if had COVID-19	547	11.0	566	11.4

[#] England and Wales, based on Office for National Statistics (ONS) definition of urban as population greater than 10,000. Scotland based on Scottish Government definition of urban as population greater than 3,000.

For peer review only

For peer review only - http://bmjopen.bmj.com/site/about/guidelines.xhtml

Table 2 Association between vaccine acceptance and sociodemographic variables – (a) bivariate results and (b) multivariate logistic regression.

	**(a) Bivariate associations between vaccine acceptance and socio-demographics** % Accepted/Intend to Accept (weighted) χ^2 test for differences by demographics					**(b) Logistic regression of vaccine acceptance** 1 = Accepted/Intend to Accept (4294), 0 = Uncertain/Refused/Intend to Refuse (600)				
	**n**	**%**		**χ^2 (df)**	**P**	**N**	**AOR***	**95% CI Lower**	**95% CI Upper**	**P**
**Gender**				2.154 (2)	.341					0.085
Male	2012	83.8				2097	ref			
Female	2117	82.5				2788	0.82	0.67	0.99	0.036
Other	5	71.4				9	0.47	0.09	2.45	0.369
**Age**				274.733 (6)	<.001					<.001
18-29	613	74.4				459	ref			
30-39 *v 18-29*	618	72.5				761	0.89	0.66	1.20	.448
40-49 *v 18-39*	640	79.3				835	1.43	1.12	1.83	.004
50-59 *v 18-49*	745	85.9				896	1.92	1.49	2.46	<.001
60-69 *v 18-59*	659	92.7				1003	3.21	2.37	4.34	<.001
70-79 *v 18-69*	629	95.7				763	3.31	2.22	4.95	<.001
80+ *v 18-79*	209	95.9				177	2.19	0.92	5.21	.078
**Education/Highest qualification**				56.056 (4)	<.001					<.001
No qualifications	495	80.1				411	ref			
Degree or equivalent and above	1811	87.2				2454	3.03	2.17	4.23	<.001
A levels / Vocational level 3 or equivalent	909	80.4				990	1.80	1.27	2.55	<.001
Other qual'ns below A level / Voc level 3	694	82.7				784	1.50	1.05	2.15	.026
Other qualification	223	73.4				255	0.90	0.58	1.39	.632
**Financial Status**				168.660 (4)	<.001					<.001
Living comfortably	1162	90.1				1533	ref			
Doing alright	1749	86.0				1998	0.89	0.69	1.15	.383
Just about getting by	848	74.9				959	0.52	0.39	0.69	<.001
Finding it quite difficult	261	77.2				266	0.74	0.50	1.10	.139
Finding it very difficult	111	63.4				138	0.35	0.22	0.55	<.001
**Country**				3.171 (2)	.205					.326
England	3581	83.5				4302	ref			
Scotland	356	80.5				384	0.82	0.59	1.13	.220
Wales	192	81.0				208	0.80	0.51	1.26	.345
**Urban/rural**				34.517 (1)	<.001					
Urban	3266	81.5				3729	ref			
Rural	863	89.4				1165	1.28	1.00	1.65	.051
**Ethnicity**				246.434 (5)	<.001					<.001
White British	3482	87.1				4226	ref			
Any other white background	254	75.8				318	0.55	0.40	0.76	<.001
Mixed or multiple ethnic groups	62	61.4				62	0.39	0.21	0.71	.002
Asian or Asian British	188	61.4				161	0.41	0.28	0.61	<.001
Black or Black British	59	58.4				67	0.25	0.14	0.43	<.001
Other	59	72.8				60	0.42	0.23	0.79	.007
**Whether been offered vaccine**				45.924 (1)	<.001					
No	3479	81.6				4227	ref			
Yes	658	91.9				667	1.73	1.24	2.43	.001
**COVID-19 Status**				72.865 (4)	<.001					<.001

For peer review only - http://bmjopen.bmj.com/site/about/guidelines.xhtml

Think probably or definitely not had COVID-19	2741	86.1				3288	ref			
Diagnosed with COVID-19	218	74.4				240	0.89	0.60	1.33	.575
Think definitely had COVID-19	118	68.2				140	0.40	0.26	0.60	<.001
Think probably had COVID-19	598	79.1				691	0.71	0.56	0.91	.006
Don’t Know if had COVID-19	462	81.5				535	0.73	0.55	0.97	.031
						Hosmer & Lemeshow χ^2= 7.444, df=8, p=0.490.<br>Final model χ^2=497.429, df=29, p<0.001<br>Nagelkerke = 0.184<br>Cases correctly classified: 88.1%.<br>84 cases excluded due to missing data on one or more independent variables.				

* adjusted for all other variables in the model, AOR, adjusted odds ratio; ref, reference category; 95%CI, 95% confidence interval

For peer review only

For peer review only - http://bmjopen.bmj.com/site/about/guidelines.xhtml

Table 3 Trust in potential sources of information on COVID-19 vaccine

Source:	Level of Trust (trust completely [1]…not at all [5]) Completely (1)		A great deal (2)		Somewhat (3)		Very little (4)		Not at all (5)		Mean	Std Dev
	n	%	n	%	n	%	n	%	n	%		
The NHS	2084	41.9	1902	38.3	701	14.1	155	3.1	127	2.5	1.86	0.95
Doctors, nurses or other healthcare professionals	1918	38.6	2092	42.1	714	14.4	154	3.1	90	1.8	1.87	0.90
Scientific and medical advisers	1798	36.2	2101	42.3	792	15.9	160	3.2	121	2.4	1.94	0.93
The World Health Organisation (WHO)	1313	26.4	2016	40.6	1070	21.6	310	6.2	256	5.1	2.23	1.07
Pharmacists	999	20.1	1973	39.7	1434	28.8	341	6.9	226	4.5	2.36	1.02
The UK Government	654	13.2	1542	31.1	1739	35.1	614	12.4	402	8.1	2.71	1.10
Scottish Govt/Welsh Assembly[a]	118	17.4	189	27.9	207	30.5	88	13.1	75	11.1	2.72	1.21
Drug companies who manufacture vaccines	406	8.2	1064	21.4	2065	41.6	771	15.5	661	13.3	3.04	1.11
Family and friends	343	6.9	876	17.6	2230	44.9	977	19.7	542	10.9	3.10	1.04
The media (e.g. newspapers, magazines, television, radio)	86	1.7	302	6.1	1567	31.5	1433	28.9	1580	31.8	3.83	1.00
Faith or community leaders	131	2.6	124	2.5	619	12.5	827	16.7	3264	65.7	4.40	0.98
Social media (e.g. Twitter, Facebook, Instagram etc)	65	1.3	69	1.4	506	10.2	1267	25.5	3056	61.6	4.45	0.83
Celebrities and social media influencers	60	1.2	71	1.4	493	9.9	1175	23.6	3170	63.8	4.47	0.82

Base: All participants (weighted). Missing cases range from n=3 to n=27. [a]Base: all participants in Scotland or Wales, n=679 (weighted). List order was randomised for each participant.

For peer review only - http://bmjopen.bmj.com/site/about/guidelines.xhtml

Table 4 Views on priority groups for vaccination: who should be first and last groups vaccinated

	Should not be offered		Priority of being offered[a]										Mean[b]	Std Dev
			One of the first (1)		(2)		(3)		(4)		One of the last (5)			
	n	%	n	%	n	%	n	%	n	%	n	%		
Doctors, nurses and other healthcare professionals	33	0.7	4472	90.0	280	5.6	83	1.7	15	0.3	83	1.7	1.17	0.63
People with serious health conditions which mean they are vulnerable to COVID-19	35	0.7	4017	80.9	671	13.5	129	2.6	35	0.7	77	1.6	1.27	0.69
Care home workers	36	0.7	3926	79.0	683	13.8	197	4.0	58	1.2	66	1.3	1.31	0.72
Residents in a care home	47	0.9	3593	72.4	734	14.8	337	6.8	123	2.5	131	2.6	1.47	0.93
People aged 80 or over	49	1.0	3613	72.9	706	14.2	304	6.1	118	2.4	168	3.4	1.48	0.96
Social care workers	33	0.7	2683	54.0	1348	27.2	683	13.8	143	2.9	75	1.5	1.70	0.92
Schoolteachers	47	0.9	2098	42.2	1621	32.6	886	17.8	223	4.5	94	1.9	1.90	0.97
People with jobs that involve direct contact with members of the public	45	0.9	1864	37.5	1603	32.3	1157	23.3	228	4.6	70	1.4	1.99	0.96
People aged 31-50	43	0.9	154	3.1	614	12.4	2096	42.2	1486	30.0	568	11.4	3.35	0.95
People aged 18-30	102	2.0	123	2.5	289	5.8	943	19.0	1375	27.7	2130	42.9	4.05	1.05
People aged under 18	282	5.7	148	3.0	253	5.1	657	13.3	831	16.8	2788	56.2	4.25	1.08

Base: All participants (weighted). [a] Missing cases range from n=11 to n=21. [b] Excludes 'should not be offered', missing cases range from n=45 to n=301. List order was randomised for each participant.

For peer review only - http://bmjopen.bmj.com/site/about/guidelines.xhtml

Supplementary material to 'A national survey of attitudes towards and intentions to vaccinate against COVID-19: implications for communications'.

Contents

- Methods S1: Questionnaire for the OPTIMising general public Uptake of a COVID-19 vaccine (OPTIMUM) study.
- Table S1 Overall response rate calculation accounting for recruitment onto original panel and panel attrition.
- Methods S2: Non-response weights.
- Table S2 Doctors, nurses or other healthcare professionals – Association between trust in sources of information about COVID-19 vaccine and socio-demographic variables – (a) bivariate results and (b) multivariate logistic regression.
- Table S3 Pharmacists – Association between trust in sources of information about COVID-19 vaccine and socio-demographic variables – (a) bivariate results and (b) multivariate logistic regression.
- Table S4 The NHS – Association between trust in sources of information about COVID-19 vaccine and socio-demographic variables – (a) bivariate results and (b) multivariate logistic regression.
- Table S5 Drug companies who manufacture vaccines – Association between trust in sources of information about COVID-19 vaccine and socio-demographic variables – (a) bivariate results and (b) multivariate logistic regression.
- Table S6 The UK Government – Association between trust in sources of information about COVID-19 vaccine and socio-demographic variables – (a) bivariate results and (b) multivariate logistic regression.
- Table S7 The Scottish/Welsh Government – Association between trust in sources of information about COVID-19 vaccine and socio-demographic variables – (a) bivariate results and (b) multivariate logistic regression.
- Table S8 Scientific and medical advisers – Association between trust in sources of information about COVID-19 vaccine and socio-demographic variables – (a) bivariate results and (b) multivariate logistic regression.
- Table S9 The World Health Organisation (WHO) – Association between trust in sources of information about COVID-19 vaccine and socio-demographic variables – (a) bivariate results and (b) multivariate logistic regression.
- Table S10 The media (e.g. newspapers, magazines, television, radio) – Association between trust in sources of information about COVID-19 vaccine and socio-demographic variables – (a) bivariate results and (b) multivariate logistic regression.
- Table S11 Social media – Association between trust in sources of information about COVID-19 vaccine and socio-demographic variables – (a) bivariate results and (b) multivariate logistic regression.
- Table S12 Celebrities and social media influencers – Association between trust in sources of information about COVID-19 vaccine and socio-demographic variables – (a) bivariate results and (b) multivariate logistic regression.
- Table S13 Family and friends – Association between trust in sources of information about COVID-19 vaccine and socio-demographic variables – (a) bivariate results and (b) multivariate logistic regression.
- Table S14 Faith or community leaders – Association between trust in sources of information about COVID-19 vaccine and socio-demographic variables – (a) bivariate results and (b) multivariate logistic regression.

For peer review only

Methods S1: Questionnaire for the OPTIMising general public Uptake of a COVID-19 vaccine (OPTIMUM) study

Socio-demographic questions
{ASK IF DemogUpd = 0}
EconAct
Which of these descriptions applied to what you spent the **most** time doing last week, that is the seven days ending last Sunday?

_WEB: “Please select one answer on every row”
_TEL: “INTERVIEWER: READ OUT EACH STATEMENT AND THE ANSWER CODES. REPEAT ANSWER CODES AS REQUIRED.”

1. In full-time education (including on vacation)
2. On government training/employment programme
3. In paid work (or away temporarily, including furlough) for at least 10 hours in week
4. Waiting to take up paid work already accepted
5. Unemployed
6. Permanently sick or disabled
7. Wholly retired from work
8. Looking after your home or family
9. Doing something else

{ASK ALL}
C19HiRsk
Since the start of the COVID-19 outbreak, have you ever been contacted by your GP or Healthcare Provider to say that you are at severe risk from COVID-19 and advised to shield?

1. Yes
2. No
3. Don’t know

{ASK ALL}
C19HiRskHH
And since the start of the COVID-19 outbreak, has anyone else in your household ever been contacted by their GP or Healthcare Provider to say that they are at severe risk from COVID-19 and advised to shield?

1. Yes
2. No
3. Don’t know

{ASK IF FF_Sex = 2 AND FF_Age LT 50}
Preg
“Are you currently pregnant?”

1. Yes
2. No

{ASK IF Cur_EconAct = 3 OR EconAct = 3}
EmpCond [MULTICODE: RANDOMISE 1…3]
“Which, if any, of the following apply to you?

_WEB: “Please select all that apply”
_TEL: “INTERVIEWER: READ OUT EACH OPTION AND CODE ALL THAT APPLY”

1. In my current job I’m required to work in close proximity with other people
2. I work in social care and have direct contact with patients or members of the public
3. I work in health care and have direct contact with patients or members of the public
4. None of these [EXCLUSIVE]

For peer review only

Vaccines

{ASK ALL}

VaccQInt

"The next set of questions will ask you about your views on vaccines in general."

DISPLAY

{ASK ALL}

VaccSafe [FLIP SCALE]

In general, how often do you think vaccines cause serious side effects?

_WEB: "Please select one answer on every row"
_TEL: "INTERVIEWER: READ OUT EACH STATEMENT AND THE ANSWER CODES. REPEAT ANSWER CODES AS REQUIRED."

1. Always
2. Frequently
3. Sometimes
4. Rarely
5. Never

{ASK ALL}

VaccMildSE [FLIP SCALE]

In general, how likely would you be to accept a vaccine that caused mild side effects?

By mild side effects we mean things like a mild fever, pain or swelling at the injection site, or feeling a bit unwell for a few days

_WEB: "Please select one answer on every row"
_TEL: "INTERVIEWER: READ OUT EACH STATEMENT AND THE ANSWER CODES. REPEAT ANSWER CODES AS REQUIRED."

1. Very likely
2. Quite likely
3. Neither likely nor unlikely
4. Quite unlikely
5. Very unlikely

{ASK ALL}

VaccEffec [FLIP SCALE]

How much protection do you think the flu vaccine provides against flu?

INTERVIEWER: READ OUT

1. Complete protection
2. A lot of protection
3. Some protection
4. A little protection
5. No protection at all

{ASK ALL}

VaccAtt [GRID; FLIP SCALE; RANDOMISE ROWS]

"{WEB: "Below are"; TEL: "I will now read out"} some statements about vaccines in general.

To what extent do you agree or disagree with each of the following?

_WEB: "Please select one answer on every row"
_TEL: "INTERVIEWER: READ OUT EACH STATEMENT AND THE ANSWER CODES. REPEAT ANSWER CODES AS REQUIRED."

S3

For peer review only

GRID ROWS

1. Government decisions about vaccines are made in people's best interests
2. My immune system is strong enough that I don't need most vaccines
3. The illnesses that vaccines prevent are not severe enough for me to get vaccinated
4. I get vaccinated because it helps to protect other people as well as me
5. I follow the recommendation of healthcare professionals when deciding whether or not to get a vaccine

GRID COLS

1. Strongly agree
2. Agree
3. Neither agree nor disagree
4. Disagree
5. Strongly disagree

{ASK ALL}
VaccAccepCh [FLIP SCALE 1…4]
Thinking about times a healthcare professional has recommended your children get a vaccine, how often have you followed that recommendation (that is, if you have any children)?

_WEB: "Please select one answer on every row"
_TEL: "INTERVIEWER: READ OUT EACH STATEMENT AND THE ANSWER CODES. REPEAT ANSWER CODES AS REQUIRED."

1. All of the time
2. Most of the time
3. Some of the time
4. None of the time
5. They have never been offered a vaccine
6. I do not have any children

{ASK ALL}
VaccAccep [FLIP SCALE 1…4]
And thinking about times a health-care professional has recommended you get a vaccine, how often have you followed that recommendation?

_WEB: "Please select one answer on every row"
_TEL: "INTERVIEWER: READ OUT EACH STATEMENT AND THE ANSWER CODES. REPEAT ANSWER CODES AS REQUIRED."

1. All of the time
2. Most of the time
3. Some of the time
4. None of the time
5. I have never been offered a vaccine

Covid-19 Vaccine
{ASK ALL}
C19VaccQInt
"The next set of questions will ask you about your views on a vaccine for COVID-19."

DISPLAY

{ASK ALL}
C19VaccOff
"Have you been offered a vaccine for COVID-19?

1. Yes
2. No

{IF C19VaccOff = 1}

For peer review only

C19VaccAcc1
"And have you had that vaccine?"

{WEB: "Please select 'Yes' if you have only had one of multiple doses"}
INTERVIEWER: "Please include if you have only had one of multiple doses"

1. Yes
2. No

{IF C19VaccAcc1 = 2}
C19VaccInt
"And do you intend to have that vaccine?"

_WEB: "Please select one answer on every row"
_TEL: "INTERVIEWER: READ OUT EACH STATEMENT AND THE ANSWER CODES. REPEAT ANSWER CODES AS REQUIRED."

1. Yes
2. No
3. Not sure

{IF C19VaccOff <> 1}
C19VaccAcc2
Would you accept the vaccine for yourself if it is offered to you?

_WEB: "Please select one answer on every row"
_TEL: "INTERVIEWER: READ OUT EACH STATEMENT AND THE ANSWER CODES. REPEAT ANSWER CODES AS REQUIRED."

1. Yes
2. No
3. Not sure

{IF C19VaccAcc2 = 3 or -8}
C19VaccAcc3
"Thank you for your response.

We would really like to know your opinion on this, even if you are unsure or don't feel you know enough.

If you had to choose, if a COVID-19 vaccine became publicly available and you were offered it, would you accept the vaccine for yourself?

_WEB: "Please select one answer on every row"
_TEL: "INTERVIEWER: READ OUT EACH STATEMENT AND THE ANSWER CODES. REPEAT ANSWER CODES AS REQUIRED."

1. Yes
2. No
3. I'm really not sure

{IF C19VaccInt = 2,3, -8 OR C19VaccAcc2 = 2 OR C19VaccAcc3 = 2,3, -8}
C19VaccWhyNo [MULTICODE: RANDOMISE 1…12]
For which, if any, of the following reasons {IF C19VaccInt = 2: "did you not"; IF C19VaccAcc2 = 2 OR C19VaccAcc3 = 2: "would you not"; IF C19VaccAcc3 = 3 or -8 or C19VaccInt =3 or -8: "are you unsure if you would"} accept a vaccine for COVID-19?

_WEB: "Please select all that apply"
_TEL: "INTERVIEWER: READ OUT EACH OPTION AND CODE ALL THAT APPLY"

1. I don't think COVID-19 is severe enough

For peer review only - http://bmjopen.bmj.com/site/about/guidelines.xhtml

2. I am concerned that vaccines are being rushed in
3. I am concerned that the vaccines have not been properly tested
4. I am frightened of needles
5. I don't feel that I have enough information about the vaccines
6. I don't think that the vaccines would be effective
7. I am worried about ingredients in the vaccines
8. I am worried that I would have a bad reaction or be allergic to it
9. I don't trust the motives of those involved in developing COVID-19 vaccines (governments, pharmaceutical companies etc.).
10. I do not believe in vaccines
11. I feel I don't need a vaccine
12. It would be inconvenient for me to get vaccinated
13. Other reason (Please describe)
14. None of these (EXCLUSIVE]

{ASK ALL}
C19VaccDec [GRID; FLIP SCALE 1…5; RANDOMISE ROWS]
How much {IF C19VaccOff = 1: "did"; IF C19VaccOff <> 1: "would"} your decision to get a COVID-19 vaccine depend on each of the following?

_WEB: "Please select one answer on every row"
_TEL: "INTERVIEWER: READ OUT EACH STATEMENT AND THE ANSWER CODES. REPEAT ANSWER CODES AS REQUIRED."

GRID ROWS
1. The country in which the vaccine is developed
2. Whether or not the vaccine is recommended by my GP/healthcare professional
3. Whether or not the vaccine is recommended by the NHS
4. Whether or not the vaccine has been tested in large trials
5. Whether or not the vaccine has been in use for a few months with no serious side-effects
6. Whether or not people I know had already had the vaccine
7. Whether or not my GP/healthcare professional had already had the vaccine
8. Whether or not my local faith leader had recommended it
9. How easy or difficult it is to get the vaccine
10. Whether or not more than one injection was needed to provide adequate protection
11. Whether or not it would allow me to get my life back (be able to go out socialising, get back to work etc)
12. Whether or not it would help to protect members of my family who are vulnerable to COVID-19

GRID COLS
1. Completely
2. A great deal
3. Somewhat
4. Very little
5. Not at all

{ASK ALL}
C19VaccAccFF [FLIP SCALE 1…5]
"Thinking about your family and friends, how many do you think would get vaccinated against COVID-19 if a vaccine was offered to them?

_WEB: "Please select one answer on every row"
_TEL: "INTERVIEWER: READ OUT EACH STATEMENT AND THE ANSWER CODES. REPEAT ANSWER CODES AS REQUIRED."

1. All of them
2. Most of them
3. About half of them
4. Some of them
5. None of them
6. Not applicable

For peer review only

{ASK ALL}
C19VaccSupFF [FLIP SCALE 1…5]
"To what extent do you think your family and friends support or oppose you getting vaccinated against COVID-19?"

_WEB: "Please select one answer on every row"
_TEL: "INTERVIEWER: READ OUT EACH STATEMENT AND THE ANSWER CODES. REPEAT ANSWER CODES AS REQUIRED."

1. Strongly oppose
2. Oppose
3. Neither oppose nor support
4. Support
5. Strongly support
6. Not applicable

{ASK ALL}
C19VaccTrstInf [GRID; FLIP SCALE; RANDOMISE ROWS]
"Thinking about {IF C19VaccOff = 1: "when"; IF C19VaccOff <> 1: "if"} you had to make a decision on whether or not to get a COVID-19 vaccine…

To what extent, if at all, would you trust information about a COVID-19 vaccine from each of the following sources?

_WEB: "Please select one answer on every row"
_TEL: "INTERVIEWER: READ OUT EACH STATEMENT AND THE ANSWER CODES. REPEAT ANSWER CODES AS REQUIRED."

GRID ROWS
1. Doctors, nurses, or other healthcare professionals
2. Pharmacists
3. The NHS
4. Drug companies who manufacture vaccines
5. The UK Government
6. {IF Cur_Country = 2: "The Scottish government"; IF Cur_Country = 3: "The Welsh Assembly"}
7. Scientific and medical advisers
8. The World Health Organisation (WHO)
9. The media (e.g. newspapers, magazines, television, radio)
10. Social media (e.g. Twitter, Facebook, Instagram etc)
11. Celebrities and social media influencers
12. Family and friends
13. Faith or community leaders

GRID COLS
1. Completely
2. A great deal
3. Somewhat
4. Very little
5. Not at all

{ASK ALL}
C19VaccPriAccep [GRID; FLIP SCALE; RANDOMISE ROWS]
A COVID-19 vaccine will be offered to some groups of people before other groups, and it is possible that not everyone in the population will be offered a COVID-19 vaccine.

How acceptable or unacceptable do you think each of the following are?

_WEB: "Please select one answer on every row"
_TEL: "INTERVIEWER: READ OUT EACH STATEMENT AND THE ANSWER CODES. REPEAT ANSWER CODES AS REQUIRED."

For peer review only - http://bmjopen.bmj.com/site/about/guidelines.xhtml

GRID ROWS
1. Some people being offered a COVID-19 vaccine before others
2. Some people not being offered a COVID-19 vaccine at all

GRID COLS
1. Very acceptable
2. Somewhat acceptable
3. Neither acceptable nor unacceptable
4. Somewhat unacceptable
5. Very unacceptable

{ASK ALL}
C19VaccPri [GRID; FLIP SCALE 1…5; RANDOMISE ROWS]
"{WEB: "Below are"; TEL: "I will now read out"} some groups that some people say should be the first to be offered a COVID-19 vaccine. For each one, how high a priority do you think it is that they get a COVID-19 vaccine, or do you not think they should be offered the vaccine at all?

Please answer on a scale of 1 to 5 where 1 means you think they should be one of the first groups to be offered the vaccine, and 5 means you think they should be one of the last groups to be offered the vaccine.

_WEB: "Please select one answer on every row"
_TEL: "INTERVIEWER: READ OUT EACH STATEMENT AND THE ANSWER CODES. REPEAT ANSWER CODES AS REQUIRED."

GRID ROWS
1. Doctors, nurses, and other healthcare professionals
2. People aged 18 to 30
3. Social care workers
4. People aged under 18
5. People with serious health conditions which mean they are vulnerable to COVID-19
6. Residents in a care home
7. Care home workers
8. People aged 80 or over
9. People aged 31-50
10. People with jobs that involve direct contact with members of the public
11. Schoolteachers

GRID COLS
1. 1 – One of the first
2. 2
3. 3
4. 4
5. 5 – One of the last
6. They should not be offered a vaccine

{ASK ALL}
C19VaccDoseImp [FLIP SCALE 1…4]
"How important, if at all, do you think it is for people to get the second injection of the COVID-19 vaccine?"

_WEB: "Please select one answer on every row"
_TEL: "INTERVIEWER: READ OUT EACH STATEMENT AND THE ANSWER CODES. REPEAT ANSWER CODES AS REQUIRED."

1. Very important
2. Fairly important
3. Not very important
4. Not at all important

Covid-19 attitudes
{ASK ALL}

For peer review only

C19VaccAttQInt
"The next set of questions will ask you about your views and experiences of COVID-19.

Some people may find these questions sensitive. Remember, you do not have to answer any questions you would prefer not to."

DISPLAY

{ASK ALL}
C19InfoEas [GRID; FLIP SCALE 1…5; RANDOMISE ROWS]
"How easy or difficult do you find each of the following?

_WEB: "Please select one answer on every row"
_TEL: "INTERVIEWER: READ OUT EACH STATEMENT AND THE ANSWER CODES. REPEAT ANSWER CODES AS REQUIRED."

GRID ROWS
1. Finding information to help you make decisions about your health
2. Finding information about how to protect yourself and others from COVID-19
3. Finding information on what to do if you have symptoms of COVID-19
4. Understanding the current instructions and guidance on how to protect yourself and others from COVID-19

GRID COLS
1. Very easy
2. Fairly easy
3. Neither easy nor difficult
4. Fairly difficult
5. Very difficult
6. Not applicable

{ASK ALL}
C19Diag
"Have you been officially diagnosed with the coronavirus (COVID-19)?"

1. Yes
2. No
3. Don't know

{IF C19Diag <> 1}
C19Had [FLIP SCALE 1…4]
"Do you think you have ever had the coronavirus (COVID-19)?"

1. Yes – definitely
2. Yes - probably
3. No – probably not
4. No – definitely not
5. Don't know

{ASK ALL}
C19Symp
"Since January 2020, have you had coronavirus (COVID-19) symptoms?

Symptoms can include a high temperature, a new continuous cough, or a loss of sense of smell or taste"

1. Yes
2. No
3. Don't know

{IF C19Symp = 1}

For peer review only

C19SympSev [FLIP SCALE]
"Would you say your symptoms were mild or severe?"

1. Mild
2. Severe

{ASK ALL}
C19Oth
"Do you have any friends or family who have had the coronavirus (COVID-19)?"

1. Yes
2. No

{IF C19Oth = 1}
C19OthHosp
"Have any of your friends or family had to go to hospital as a result of having the coronavirus (COVID-19)?"

1. Yes
2. No
3. Prefer not to say

{IF C19Oth = 1}
C19OthDied
"Have any of your friends or family died as a result of having the coronavirus (COVID-19)?"

1. Yes
2. No
3. Prefer not to say

{ASK ALL}
C19Imp [FLIP SCALE]
"Thinking about the impact the COVID-19 pandemic has had on different areas of your life…

How much of a negative impact, if any, would you say the COVID-19 pandemic has had on your life?"

_WEB: "Please select one answer on every row"
_TEL: "INTERVIEWER: READ OUT EACH STATEMENT AND THE ANSWER CODES. REPEAT ANSWER CODES AS REQUIRED."

1. An extremely negative impact
2. A very negative impact
3. A somewhat negative impact
4. A slightly negative impact
5. It has not had a negative impact

{ASK ALL}
C19Fut1 [FLIP SCALE 1-5]
"How likely or unlikely do you think you are to get COVID-19 in the next 6 months?

_WEB: "Please select one answer on every row"
_TEL: "INTERVIEWER: READ OUT EACH STATEMENT AND THE ANSWER CODES. REPEAT ANSWER CODES AS REQUIRED."

1. Very likely
2. Quite likely
3. Neither likely nor unlikely
4. Quite unlikely
5. Very unlikely
6. Don't know

For peer review only

For peer review only - http://bmjopen.bmj.com/site/about/guidelines.xhtml

{ASK IF C19Fut = 6}
C19Fut1DK [FLIP SCALE 1-5]
Thank you for your response.

We would really like to know your opinion on this, even if you are unsure or don't feel you have enough information.

If you had to decide, how likely or unlikely do you think you are to get COVID-19 in the next 6 months?

_WEB: "Please select one answer on every row"
_TEL: "INTERVIEWER: READ OUT EACH STATEMENT AND THE ANSWER CODES. REPEAT ANSWER CODES AS REQUIRED."

1. Very likely
2. Quite likely
3. Neither likely nor unlikely
4. Quite unlikely
5. Very unlikely
6. I really don't know

{ASK ALL}
C19Fut2 [FLIP SCALE 1-5]
If you did get COVID-19 in the next 6 months, how likely or unlikely do you think you would be to become seriously ill as a result of it?

_WEB: "Please select one answer on every row"
_TEL: "INTERVIEWER: READ OUT EACH STATEMENT AND THE ANSWER CODES. REPEAT ANSWER CODES AS REQUIRED."

1. Very likely
2. Quite likely
3. Neither likely nor unlikely
4. Quite unlikely
5. Very unlikely
6. Don't know

{ASK IF C19Fut2=6}
C19Fut2DK [FLIP SCALE 1-5]
Thank you for your response.

We would really like to know your opinion on this, even if you are unsure or don't feel you have enough information.

If you had to decide, if you did get COVID-19 in the next 6 months, how likely or unlikely do you think you would be to become seriously ill as a result of it?

_WEB: "Please select one answer on every row"
_TEL: "INTERVIEWER: READ OUT EACH STATEMENT AND THE ANSWER CODES. REPEAT ANSWER CODES AS REQUIRED."

1. Very likely
2. Quite likely
3. Neither likely nor unlikely
4. Quite unlikely
5. Very unlikely
6. I really don't know

{ASK ALL}

For peer review only

For peer review only - http://bmjopen.bmj.com/site/about/guidelines.xhtml

C19PrvDon [GRID: RANDOMISE ROWS; FLIP SCALE 1…5]
"How often do you currently do each of the following to help prevent the spread of COVID-19?"

_WEB: "Please select one answer on every row"
_TEL: "INTERVIEWER: READ OUT EACH STATEMENT AND THE ANSWER CODES. REPEAT ANSWER CODES AS REQUIRED."

GRID ROWS
1. Try to stay physically distant from other people when I am out in public
2. Avoid crowded public places
3. Wash my hands with soap and water for at least 20 seconds
4. Wear a face covering whenever in shops or on public transport
5. Obey the rules about how many people from different households can meet indoors
6. Obey the rules about how many people from different households can meet outdoors
7. Register my contact details when I visit cafes, restaurants or bars

GRID COLS
1. Always
2. Often
3. Sometimes
4. Rarely
5. Never
6. Not applicable

{ASK ALL}
C19Att [GRID: RANDOMISE ROWS; FLIP SCALE]
To what extent do you agree or disagree with each of the following statements about COVID-19?

_WEB: "Please select one answer on every row"
_TEL: "INTERVIEWER: READ OUT EACH STATEMENT AND THE ANSWER CODES. REPEAT ANSWER CODES AS REQUIRED."

GRID ROWS
1. Thinking about COVID-19 makes me feel worried
2. I am worried that I or people I care about will get sick from COVID-19
3. In general, the seriousness of COVID-19 is being exaggerated
4. COVID-19 feels like something far away from me
5. The {IF Cur_Country = -1,1: "UK"; IF Cur_Country = 2: "Scottish"; IF Cur_Country = 3: "Welsh Assembly"} Government's response to COVID-19 is doing more harm than the disease itself
6. COVID-19 is a hoax

GRID COLS
1. Strongly agree
2. Agree
3. Neither agree nor disagree
4. Disagree
5. Strongly disagree

{ASK ALL}
C19Acc [GRID: RANDOMISE ROWS; FLIP SCALE]
"How acceptable or unacceptable do you find each of the following options for addressing COVID-19 in the next 12 months?

_WEB: "Please select one answer on every row"
_TEL: "INTERVIEWER: READ OUT EACH STATEMENT AND THE ANSWER CODES. REPEAT ANSWER CODES AS REQUIRED."

GRID ROWS
1. Encouraging the general public to get vaccinated against COVID-19
2. Bringing in restrictions from time to time to stop the spread of COVID-19

For peer review only

3. Using test and trace systems to control the spread of COVID-19
4. Letting COVID-19 run its course through the population
5. Modifying our behaviour to live with COVID-19
6. Shielding of vulnerable people and letting everyone else get on with their lives

GRID COLS

1. Very acceptable
2. Somewhat acceptable
3. Neither acceptable nor unacceptable
4. Somewhat unacceptable
5. Very unacceptable

For peer review only

For peer review only - http://bmjopen.bmj.com/site/about/guidelines.xhtml

Table S1 Overall response rate calculation accounting for recruitment onto original panel and panel attrition.

	Wave of British Social Attitudes Survey (BSA) from which panel was recruited			
**Response to initial BSA survey**	**2018**	**2019**	**2020**	**Total 2018 to 2020**
BSA issued	10,270	7,956	42066	60,292
BSA deadwood	1,023	684	4207	5,914
BSA productive	3,879	3,224	3964	11,067
BSA response rate	42%	44%	10%	20%
**Overall response for panel recruitment**				
BSA productive	3,879	3,224	3964	11,067
Recruited to panel	2,412	2,104	3086	7,602
Panel recruitment rate	62%	65%	78%	69%
Panel deadwood	19	7	0	26
Panel lost to attrition/inactivity prior to vaccine survey	969	673	3	1645
**Panel's response to vaccine survey**				
Issued	1,424	1,424	3,083	5,931
Deadwood	1	5	0	6
Achieved	1,242	1,181	2,555	4,978
**Vaccine survey response rate**	**87%**	**83%**	**83%**	**84%**
**Overall survey response rate[a]**	**13%**	**16%**	**7%**	**9%**

[a] Response rate accounting for non-response at original point of recruitment (British Social Attitudes Survey 2018, 2019 or 2020; http://bsa.natcen.ac.uk) and panel attrition thereafter.

For peer review only

Methods S2: Non-response weights

Non-response to NatCen's probability panel surveys can occur at any one of three stages: the survey used for recruitment to the panel (the British Social Attitudes survey), the invitation to join the panel (at the end of the BSA interview) and the survey of panel members itself. The BSA survey is already weighted to adjust for non-response and we compute further weights to take account of non-response at each of the two subsequent stages. The final weights are the product of these three weights. This three-stage approach is ideal because the correlates of non-response can be different at each stage. With this system we also can optimise the use of the data available from the British Social Attitudes Survey (BSA).

These are the three weights we have computed:

1. **BSA survey weight:** the panel members were recruited from BSA 2018, 2019 and 2020. The weighting process for BSA 2020 was a little different from the other years due to the change in methodology due to the COVID-19 pandemic (using a 'push-to-web' methodology, with up to two participants in a household allowed to take part). All three years required weights to adjust for differential selection probabilities (design weights), non-response at household level (non-response weights) and weights to adjust the profile of respondents to match population estimates (calibration weights). We now describe in more detail the approaches used in 2018/19 and 2020.

   For 2018/2019, a non-response model was estimated to adjust for household level non-response. The model included region, dwelling type, percentage of owner-occupied properties in the postcode sector (grouped) and population density. The model produced a non-response weight, which was combined with the design weights (which accounted for unequal selection probabilities of households and individuals within households) to produce a composite weight. This weight was then adjusted using calibration weighting so that the profile of BSA respondents matches the British population in terms of age, sex and region.[a]

   As above, the weighting process for BSA 2020 was a little different from previous years due to the methodology used. Two non-response models were created: one to adjust for household level non-response (as in previous years), and another to account for differential response within households. The first model included (grouped) census variables measuring percentage of owner occupied properties, percentage of adults with a degree and percentage of BAME individuals in the postcode sector, plus region and the geo-demographic Output Area Classification. The second model included region, household tenure, household income (grouped), number of eligible adults and IMD tertiles. Each model produced a non-response weight and these were combined to produce a composite weight. This weight was then adjusted using calibration weighting so that the profile of BSA respondents matches the GB population in terms of age, gender, highest educational qualification, tenure and region.

2. **Panel weight:** this weight accounts for non-response at the panel recruitment stage where some people interviewed as part of the BSA survey chose not to join the panel. A logistic regression model has been used to derive the probability of response of each panel member; the panel weight is computed as the inverse of the probabilities of response. This weight adjusts the panel for non-response using the following variables: age and sex groups, region, BSA year, household type, household income, education level, internet access, ethnicity, tenure, social class group, economic activity, political party identification, and interest in politics.[b,c] The resulting panel weight has been multiplied by the BSA weights, so the panel is representative of the population.

3. **Survey weight:** this weight is to adjust the bias caused by non-response to this particular panel survey. A logistic regression model has been used to compute the probabilities of response of each participant. The panel survey weight is equal to the inverse of the probabilities of response. The initial set of predictors used to build the model was the same as for the panel weight; and at this wave the final set of variables used was also the same. Unlike the model used to calculate the panel weight, no interaction term between BSA survey year and internet access was used. As this wave of data collection was web-only, there were only a very small number of panellists (from each year of BSA) who took part in the survey but did not have access to the internet when they were interviewed for BSA. It was therefore deemed inappropriate to include the interaction term in the model.

   The final survey weight is the result of multiplying the survey weight by the compounded panel weight.

Notes:
a. More details on the BSA weight can be found at http://bsa.natcen.ac.uk/
b. The characteristics that are likely to change with time for an individual and whose distribution differed between 2018 and 2020 BSA sample have been entered into the model in interaction with BSA year.
c. More details about these variables, the question wording and the full dataset can be found at http://bsa.natcen.ac.uk/

For peer review only - http://bmjopen.bmj.com/site/about/guidelines.xhtml

Table S2 Doctors, nurses or other healthcare professionals – Association between trust in sources of information about COVID-19 vaccine and socio-demographic variables – (a) bivariate results and (b) multivariate logistic regression.

	**(a) Bivariate associations between socio-demographics and trusting COVID-19 vaccine info from Doctors, nurses or other healthcare professionals** % Trust completely or a great deal (weighted) χ^2 test for differences by demographics				**(b) Logistic regression of trust in COVID-19 vaccine info from Doctors, nurses or other healthcare professionals** 1 = Trust completely or a great deal (4104), 0 = Trust somewhat, very little or not at all (786)				
	**n**	**%**	**χ^2 (df)**	**P**	**N**	**AOR***	**95% CI Lower**	**95% CI Upper**	**P**
**Gender**									
Male	1957	81.7	3.020 (2)	.221	2097	ref			.526
Female	2046	79.9			2784	0.92	0.79	1.08	.331
Other	5	71.4			9	0.59	0.12	2.94	.518
**Age**									
18-29	649	78.8	53.883 (6)	<.001	459	ref			<.001
30-39 *v 18-29*	642	75.7			758	0.83	0.61	1.12	.219
40-49 *v 18-39*	629	78.4			835	0.96	0.76	1.22	.750
50-59 *v 18-49*	701	80.9			896	1.18	0.95	1.47	.133
60-69 *v 18-59*	599	84.2			1004	1.40	1.12	1.74	.003
70-79 *v 18-69*	582	89.0			761	1.72	1.32	2.24	<.001
80+ *v 18-79*	181	83.0			177	1.32	0.83	2.11	.240
**Education/Highest qualification**									
No qualifications	440	71.7	91.917 (4)	<.001	408	ref			<.001
Degree or equivalent and above	1775	85.7			2454	2.64	2.00	3.48	<.001
A levels / Vocational level 3 or equivalent	906	80.0			990	1.87	1.39	2.51	<.001
Other qual'ns below A level / Voc level 3	673	80.5			783	1.70	1.25	2.29	.001
Other qualification	210	69.1			255	0.97	0.67	1.40	.872
**Financial Status**									
Living comfortably	1121	87.2	124.251 (4)	<.001	1533	ref			<.001
Doing alright	1699	83.7			1995	0.90	0.73	1.10	.296
Just about getting by	824	72.9			959	0.61	0.48	0.77	<.001
Finding it quite difficult	247	73.3			266	0.60	0.42	0.84	.003
Finding it very difficult	116	66.7			137	0.51	0.33	0.78	.002
**Country**									
England	3499	81.7	21.523 (2)	<.001	4299	ref			.128
Scotland	321	73.0			383	0.77	0.59	1.02	.068
Wales	183	77.2			208	0.82	0.56	1.20	.308
**Urban/rural**									
Urban	3201	80.1	4.443 (1)	.035	3725	ref			
Rural	802	83.1			1165	0.97	0.80	1.18	.778
**Ethnicity**									
White British	3314	83.0	63.871 (5)	<.001	4224	ref			<.001
Any other white background	242	72.7			317	0.54	0.41	0.72	<.001
Mixed or multiple ethnic groups	64	66.0			62	0.38	0.22	0.67	<.001
Asian or Asian British	223	73.4			160	0.53	0.36	0.77	<.001
Black or Black British	70	69.3			67	0.41	0.24	0.70	.001
Other	58	71.6			60	0.56	0.30	1.03	.060
					Hosmer & Lemeshow χ^2= 10.236, df=8, p=0.249. Final model χ^2=220.263, df=24, p<0.001 Nagelkerke = 0.075 Cases correctly classified: 84.0%. 88 cases excluded due to missing data on one or more independent variables.				

* adjusted for all other variables in the model, AOR, adjusted odds ratio; ref, reference category; 95% CI, 95% confidence interval.

For peer review only

For peer review only - http://bmjopen.bmj.com/site/about/guidelines.xhtml

Table S3 Pharmacists – Association between trust in sources of information about COVID-19 vaccine and socio-demographic variables – (a) bivariate results and (b) multivariate logistic regression.

	**(a) Bivariate associations between socio-demographics and trusting COVID-19 vaccine info from Pharmacists** % Trust completely or a great deal (weighted) χ^2 test for differences by demographics					**(b) Logistic regression of trust in COVID-19 vaccine info from Pharmacists** 1 = Trust completely or a great deal (3107), 0 = Trust somewhat, very little or not at all (1786)				
	**n**	**%**		**χ^2 (df)**	**P**	**N**	**AOR***	**95% CI Lower**	**95% CI Upper**	**P**
**Gender**										
Male	1420	59.2		1.597 #	.474	2098	ref			.620
Female	1548	60.4				2786	1.06	0.94	1.19	.376
Other	3	42.9				9	0.78	0.21	2.95	.715
**Age**										
18-29	461	55.9		29.783 (6)	<.001	459	ref			.092
30-39 *v 18-29*	486	57.2				759	0.98	0.77	1.24	.848
40-49 *v 18-39*	455	56.7				835	0.96	0.80	1.16	.662
50-59 *v 18-49*	518	59.7				896	1.18	0.99	1.39	.062
60-69 *v 18-59*	462	65.0				1004	1.24	1.06	1.46	.009
70-79 *v 18-69*	428	65.1				763	1.00	0.84	1.19	.972
80+ *v 18-79*	144	65.8				177	1.12	0.80	1.55	.514
**Education/Highest qualification**										
No qualifications	349	56.7		25.123 (4)	<.001	410	ref			<.001
Degree or equivalent and above	1305	63.0				2454	1.34	1.07	1.67	.012
A levels / Vocational level 3 or equivalent	680	60.1				990	1.20	0.94	1.53	.146
Other qual'ns below A level / Voc level 3	485	57.9				784	1.02	0.79	1.31	.890
Other qualification	151	49.8				255	0.82	0.59	1.13	.216
**Financial Status**										
Living comfortably	883	68.7		78.993 (4)	<.001	1533	ref			<.001
Doing alright	1220	60.0				1997	0.77	0.66	0.89	<.001
Just about getting by	607	53.6				960	0.63	0.53	0.75	<.001
Finding it quite difficult	178	52.8				266	0.62	0.47	0.82	.001
Finding it very difficult	81	46.8				137	0.51	0.35	0.73	<.001
**Country**										
England	2589	60.4		7.095 (2)	.029	4301	ref			.673
Scotland	239	54.1				384	0.91	0.73	1.13	.403
Wales	137	57.8				208	0.95	0.71	1.27	.721
**Urban/rural**										
Urban	2349	58.7		8.096 (1)	.004	3728				
Rural	615	63.7				1165	1.04	0.90	1.20	.599
**Ethnicity**										
White British	2516	62.9		91.005 (5)	<.001	4226	ref			<.001
Any other white background	157	46.7				318	0.57	0.45	0.72	<.001
Mixed or multiple ethnic groups	39	40.2				62	0.42	0.25	0.70	<.001
Asian or Asian British	142	46.6				160	0.47	0.34	0.65	<.001
Black or Black British	51	50.5				67	0.63	0.39	1.03	.067
Other	35	43.2				60	0.48	0.29	0.81	.006
						Hosmer & Lemeshow χ^2= 11.453, df=8, p=0.177.<br>Final model χ^2=157.815, df=24, p<0.001<br>Nagelkerke = .043<br>Cases correctly classified: 64.5%.<br>85 cases excluded due to missing data on one or more independent variables.				

* adjusted for all other variables in the model, AOR, adjusted odds ratio; ref, reference category; 95% CI, 95% confidence interval. # Fisher-Freeman-Halton Exact Test.

For peer review only - http://bmjopen.bmj.com/site/about/guidelines.xhtml

Table S4 The NHS – Association between trust in sources of information about COVID-19 vaccine and socio-demographic variables – (a) bivariate results and (b) multivariate logistic regression.

	**(a) Bivariate associations between socio-demographics and trusting COVID-19 vaccine info from the NHS** % Trust completely or a great deal (weighted) χ 2 test for differences by demographics					**(b) Logistic regression of trust in COVID-19 vaccine info from the NHS** 1 = Trust completely or a great deal (4115), 0 = Trust somewhat, very little or not at all (775)				
	**n**	**%**		**χ^2 (df)**	**P**	**N**	**AOR***	**95% CI Lower**	**95% CI Upper**	**P**
**Gender**										
Male	1926	80.3		1.598 (2)	.450	2097	ref			.461
Female	2055	80.2				2784	0.97	0.82	1.14	.718
Other	5	62.5				9	0.41	0.10	1.74	.225
**Age**										
18-29	609	73.9		106.785 (6)	<.001	459	ref			<.001
30-39 *v 18-29*	632	74.4				759	0.94	0.70	1.26	.672
40-49 *v 18-39*	618	77.1				834	1.05	0.84	1.32	.672
50-59 *v 18-49*	710	81.9				895	1.45	1.16	1.81	.001
60-69 *v 18-59*	609	85.7				1004	1.62	1.29	2.03	<.001
70-79 *v 18-69*	593	90.5				762	1.99	1.50	2.63	<.001
80+ *v 18-79*	186	85.3				177	1.66	0.99	2.79	.056
**Education/Highest qualification**										
No qualifications	447	72.6		60.407 (4)	<.001	410	ref			<.001
Degree or equivalent and above	1733	83.7				2452	2.39	1.80	3.16	<.001
A levels / Vocational level 3 or equivalent	909	80.4				990	1.95	1.44	2.64	<.001
Other qual'ns below A level / Voc level 3	679	81.2				783	1.78	1.30	2.43	<.001
Other qualification	211	69.6				255	0.95	0.65	1.39	.805
**Financial Status**										
Living comfortably	1136	88.3		167.221 (4)	<.001	1533	ref			<.001
Doing alright	1675	82.4				1996	0.74	0.59	0.92	.006
Just about getting by	823	72.8				958	0.47	0.37	0.60	<.001
Finding it quite difficult	248	73.6				266	0.51	0.36	0.72	<.001
Finding it very difficult	99	56.9				137	0.31	0.21	0.47	<.001
**Country**										
England	3459	80.8		6.736 (2)	.034	4298	ref			.505
Scotland	345	78.1				384	0.98	0.73	1.32	.900
Wales	177	74.7				208	0.80	0.54	1.17	.243
**Urban/rural**										
Urban	3165	79.2		14.722 (1)	<.001	3725				
Rural	817	84.7				1165	1.03	0.84	1.26	.791
**Ethnicity**										
White British	3331	83.3		126.307 (5)	<.001	4225	ref			<.001
Any other white background	224	67.3				317	0.47	0.36	0.63	<.001
Mixed or multiple ethnic groups	67	69.1				62	0.48	0.27	0.86	.014
Asian or Asian British	207	68.1				160	0.57	0.39	0.84	.004
Black or Black British	60	60.0				66	0.38	0.22	0.65	<.001
Other	57	70.4				60	0.45	0.25	0.81	.008
						Hosmer & Lemeshow χ^2= 8.677, df=8, p=0.370.<br>Final model χ^2=291.002, df=24, p<0.001<br>Nagelkerke = .099<br>Cases correctly classified: 84.3%.<br>88 cases excluded due to missing data on one or more independent variables.				

* adjusted for all other variables in the model, AOR, adjusted odds ratio; ref, reference category; 95% CI, 95% confidence interval.

For peer review only - http://bmjopen.bmj.com/site/about/guidelines.xhtml

Table S5 Drug companies who manufacture vaccines – Association between trust in sources of information about COVID-19 vaccine and socio-demographic variables – (a) bivariate results and (b) multivariate logistic regression.

	**(a) Bivariate associations between socio-demographics and trusting COVID-19 vaccine info from drug companies who manufacture vaccines** % Trust completely or a great deal (weighted) χ^2 test for differences by demographics				**(b) Logistic regression of trust in COVID-19 vaccine info from drug companies who manufacture vaccines** 1 = Trust completely or a great deal (1416), 0 = Trust somewhat, very little or not at all (3473)				
	**n**	**%**	**χ^2 (df)**	**P**	**N**	**AOR***	**95% CI Lower**	**95% CI Upper**	**P**
**Gender**									
Male	652	27.2	16.276#	<.001	2096	ref			<.001
Female	819	32.0			2784	1.28	1.12	1.45	<.001
Other	0	0.0			9	0.00	0.00	.	.999
**Age**									
18-29	264	32.0	32.180 (6)	<.001	459	ref			.030
30-39 *v 18-29*	249	29.3			759	0.91	0.71	1.18	.480
40-49 *v 18-39*	196	24.5			834	0.72	0.59	0.89	.002
50-59 *v 18-49*	226	26.1			896	0.87	0.72	1.04	.120
60-69 *v 18-59*	219	30.8			1004	0.96	0.81	1.13	.613
70-79 *v 18-69*	233	35.6			760	1.12	0.94	1.34	.204
80+ *v 18-79*	76	34.7			177	1.02	0.73	1.42	.905
**Education/Highest qualification**									
No qualifications	217	35.3	17.807 (4)	.001	408	ref			.004
Degree or equivalent and above	569	27.5			2454	0.72	0.57	0.92	.007
A levels / Vocational level 3 or equivalent	321	28.4			990	0.78	0.60	1.01	.057
Other qual'ns below A level / Voc level 3	267	31.9			783	0.97	0.75	1.26	.844
Other qualification	97	32.1			254	0.94	0.67	1.32	.732
**Financial Status**									
Living comfortably	444	34.5	20.183 (4)	<.001	1533	ref			<.001
Doing alright	565	27.8			1995	0.72	0.62	0.83	<.001
Just about getting by	313	27.7			958	0.66	0.55	0.80	<.001
Finding it quite difficult	98	29.0			266	0.74	0.55	1.00	.054
Finding it very difficult	50	29.1			137	0.55	0.36	0.85	.007
**Country**									
England	1259	29.4	2.325 (2)	.313	4297	ref			.842
Scotland	130	29.3			384	1.00	0.79	1.26	.996
Wales	81	34.0			208	1.10	0.81	1.49	.559
**Urban/rural**									
Urban	1187	29.7	.092 (1)	.762	3724				
Rural	282	29.2			1165	0.99	0.85	1.15	.868
**Ethnicity**									
White British	1234	30.9	29.028 (5)	<.001	4223	ref			.012
Any other white background	70	20.9			318	0.63	0.48	0.84	.002
Mixed or multiple ethnic groups	20	20.6			62	0.92	0.52	1.62	.769
Asian or Asian British	83	27.2			160	0.74	0.50	1.08	.119
Black or Black British	35	34.7			67	1.11	0.64	1.91	.714
Other	12	15.2			59	0.55	0.28	1.07	.078
					Hosmer & Lemeshow χ^2= 9.180, df=8, p=0.327. Final model χ^2=96.401, df=24, p<0.001 Nagelkerke = .028 Cases correctly classified: 71.0%. 89 cases excluded due to missing data on one or more independent variables.				

* adjusted for all other variables in the model, AOR, adjusted odds ratio; ref, reference category; 95% CI, 95% confidence interval. # Fisher-Freeman-Halton Exact Test.

For peer review only - http://bmjopen.bmj.com/site/about/guidelines.xhtml

Table S6 The UK Government – Association between trust in sources of information about COVID-19 vaccine and socio-demographic variables – (a) bivariate results and (b) multivariate logistic regression.

	**(a) Bivariate associations between socio-demographics and trusting COVID-19 vaccine info from the UK Government** % Trust completely or a great deal (weighted) χ^2 test for differences by demographics					**(b) Logistic regression of trust in COVID-19 vaccine info from the UK Government** 1 = Trust completely or a great deal (2279), 0 = Trust somewhat, very little or not at all (2607)				
	**n**	**%**		**χ^2 (df)**	**P**	**N**	**AOR***	**95% CI Lower**	**95% CI Upper**	**P**
**Gender**										
Male	1114	46.6		10.393[#]	.005	2096	ref			.012
Female	1080	42.3				2781	0.84	0.75	0.95	.004
Other	2	25.0				9	0.47	0.09	2.37	.363
**Age**										
18-29	261	31.9		182.080 (6)	<.001	458	ref			<.001
30-39 *v 18-29*	290	34.2				758	1.14	0.89	1.47	.289
40-49 *v 18-39*	332	41.5				834	1.37	1.14	1.65	<.001
50-59 *v 18-49*	416	48.0				895	1.67	1.41	1.97	<.001
60-69 *v 18-59*	373	52.8				1003	1.46	1.25	1.70	<.001
70-79 *v 18-69*	383	58.6				762	1.65	1.40	1.96	<.001
80+ *v 18-79*	124	57.1				176	1.54	1.13	2.11	.007
**Education/Highest qualification**										
No qualifications	269	43.7		2.856 (4)	.582	410	ref			.439
Degree or equivalent and above	924	44.8				2450	0.95	0.76	1.18	.622
A levels / Vocational level 3 or equivalent	482	42.6				990	1.04	0.82	1.33	.733
Other qual'ns below A level / Voc level 3	386	46.2				783	1.07	0.83	1.37	.601
Other qualification	134	45.1				253	1.14	0.83	1.58	.422
**Financial Status**										
Living comfortably	704	54.8		94.512 (4)	<.001	1533	ref			<.001
Doing alright	891	43.9				1994	0.76	0.66	0.87	<.001
Just about getting by	415	36.9				957	0.54	0.45	0.64	<.001
Finding it quite difficult	124	36.9				266	0.55	0.42	0.73	<.001
Finding it very difficult	60	36.1				136	0.40	0.27	0.59	<.001
**Country**										
England	1928	45.1		19.887 (2)	<.001	4295	ref			.003
Scotland	151	34.5				383	0.70	0.56	0.88	.002
Wales	114	48.3				208	1.18	0.89	1.58	.253
**Urban/rural**										
Urban	1741	43.8		2.886 (1)	.089	3721				
Rural	451	46.8				1165	1.00	0.87	1.15	.969
**Ethnicity**										
White British	1832	45.9		35.180 (5)	<.001	4224	ref			.074
Any other white background	104	31.2				317	0.70	0.55	0.90	.005
Mixed or multiple ethnic groups	31	32.0				62	0.73	0.43	1.26	.258
Asian or Asian British	126	42.6				158	1.14	0.82	1.59	.430
Black or Black British	40	40.0				66	1.07	0.64	1.77	.803
Other	31	39.2				59	1.03	0.60	1.76	.910
						Hosmer & Lemeshow χ^2= 9.412, df=8, p=0.309. Final model χ^2=258.301, df=24, p<0.001 Nagelkerke = .069 Cases correctly classified: 60.3%. 92 cases excluded due to missing data on one or more independent variables.				

* adjusted for all other variables in the model, AOR, adjusted odds ratio; ref, reference category; 95% CI, 95% confidence interval. [#] Fisher-Freeman-Halton Exact Test.

For peer review only - http://bmjopen.bmj.com/site/about/guidelines.xhtml

Table S7 The Scottish/Welsh Government – Association between trust in sources of information about COVID-19 vaccine and socio-demographic variables – (a) bivariate results and (b) multivariate logistic regression.

	**(a) Bivariate associations between socio-demographics and trusting COVID-19 vaccine info from the Scottish/Welsh Government** % Trust completely or a great deal (weighted) χ^2 test for differences by demographics					**(b) Logistic regression of trust in COVID-19 vaccine info from the Scottish/Welsh Government** 1 = Trust completely or a great deal (289), 0 = Trust somewhat, very little or not at all (297)				
	**n**	**%**		**χ^2 (df)**	**P**	**N**	**AOR***	**95% CI Lower**	**95% CI Upper**	**P**
**Gender**										
Male	173	49.0		3.935 (1)	.047	274	ref			
Female	132	41.4				312	0.89	0.63	1.25	.491
**Age**										
18-29	61	50.8		8.885 (6)	.180	63	ref			.249
30-39 *v 18-29*	45	44.1				93	1.27	0.65	2.48	.485
40-49 *v 18-39*	36	36.7				95	0.87	0.51	1.49	.617
50-59 *v 18-49*	75	52.1				117	1.66	1.03	2.67	.037
60-69 *v 18-59*	46	46.5				113	1.06	0.67	1.68	.790
70-79 *v 18-69*	32	43.8				81	0.78	0.46	1.32	.353
80+ *v 18-79*	10	33.3				24	0.63	0.26	1.50	.297
**Education/Highest qualification**										
No qualifications	29	27.9		31.212 (4)	<.001	52	ref			.042
Degree or equivalent and above	144	56.7				280	2.18	1.12	4.23	.021
A levels / Vocational level 3 or equivalent	79	47.3				135	1.95	0.97	3.95	.062
Other qual'ns below A level / Voc level 3	34	36.6				81	1.27	0.60	2.69	.538
Other qualification	18	35.3				38	1.03	0.42	2.53	.941
**Financial Status**										
Living comfortably	84	56.0		8.356 (4)	.079	167	ref			.153
Doing alright	118	42.4				256	0.69	0.46	1.05	.081
Just about getting by	71	43.3				114	0.62	0.37	1.05	.074
Finding it quite difficult	22	41.5				34	0.49	0.22	1.09	.078
Finding it very difficult	11	45.8				15	0.34	0.10	1.16	.083
**Country**										
Scotland	206	46.9		1.208 (1)	.272	381	ref			
Wales	99	42.5				205	1.09	0.76	1.57	.642
**Urban/rural**										
Urban	233	46.9		1.719 (1)	.190	409				
Rural	72	41.1				177	0.62	0.42	0.91	.015
**Ethnicity**										
White British	270	45.3		.147 (1)	.702	535	ref			
Other than white British	32	47.8				51	1.03	0.55	1.92	.932
						Hosmer & Lemeshow χ^2= 12.017, df=8, p=0.150.<br>Final model χ^2=35.151, df=18, p=0.009<br>Nagelkerke = .078<br>Cases correctly classified: 61.4%.<br>16 cases excluded due to missing data on one or more independent variables.				

* adjusted for all other variables in the model, AOR, adjusted odds ratio; ref, reference category; 95% CI, 95% confidence interval. [#] Fisher-Freeman-Halton Exact Test.

For peer review only

Table S8 Scientific and medical advisers – Association between trust in sources of information about COVID-19 vaccine and socio-demographic variables – (a) bivariate results and (b) multivariate logistic regression.

	**(a) Bivariate associations between socio-demographics and trusting COVID-19 vaccine info from scientific and medical advisers** % Trust completely or a great deal (weighted) χ^2 test for differences by demographics					**(b) Logistic regression of trust in COVID-19 vaccine info from scientific and medical advisers** 1 = Trust completely or a great deal (4008), 0 = Trust somewhat, very little or not at all (884)				
	**n**	**%**		**χ^2 (df)**	**P**	**N**	**AOR***	**95% CI Lower**	**95% CI Upper**	**P**
**Gender**										
Male	1885	78.6		0.302 (2)	.860	2097	ref			.544
Female	2006	78.2				2786	1.08	0.93	1.26	.320
Other	5	71.4				9	0.71	0.14	3.51	.674
**Age**										
18-29	644	78.2		11.885 (6)	.065	459	ref			.850
30-39 *v 18-29*	643	75.6				759	0.86	0.63	1.17	.323
40-49 *v 18-39*	631	78.6				835	1.06	0.84	1.34	.640
50-59 *v 18-49*	677	78.1				896	1.09	0.88	1.36	.426
60-69 *v 18-59*	572	80.5				1004	1.03	0.84	1.26	.785
70-79 *v 18-69*	540	82.2				763	1.05	0.84	1.32	.654
80+ *v 18-79*	166	76.1				176	1.10	0.72	1.68	.650
**Education/Highest qualification**										
No qualifications	389	63.1		147.739 (4)	<.001	410	ref			<.001
Degree or equivalent and above	1743	84.1				2453	3.21	2.50	4.13	<.001
A levels / Vocational level 3 or equivalent	905	80.0				990	2.70	2.04	3.55	<.001
Other qual'ns below A level / Voc level 3	653	77.8				784	1.98	1.51	2.61	<.001
Other qualification	205	67.7				255	1.23	0.87	1.73	.245
**Financial Status**										
Living comfortably	1110	86.4		154.081 (4)	<.001	1532	ref			<.001
Doing alright	1640	80.6				1997	0.65	0.53	0.80	<.001
Just about getting by	797	70.4				960	0.46	0.36	0.58	<.001
Finding it quite difficult	249	73.9				266	0.51	0.36	0.72	<.001
Finding it very difficult	97	55.7				137	0.32	0.21	0.48	<.001
**Country**										
England	3375	78.8		3.260 (2)	.196	4300	ref			.068
Scotland	341	77.0				384	1.03	0.77	1.37	.843
Wales	176	74.3				208	0.67	0.47	0.94	.022
**Urban/rural**										
Urban	3108	77.7		5.368 (1)	.021	3727				
Rural	783	81.1				1165	1.00	0.83	1.20	.978
**Ethnicity**										
White British	3236	80.9		87.036 (5)	<.001	4225	ref			<.001
Any other white background	244	72.8				318	0.56	0.42	0.75	<.001
Mixed or multiple ethnic groups	68	70.8				62	0.51	0.28	0.92	.025
Asian or Asian British	203	66.6				160	0.46	0.32	0.67	<.001
Black or Black British	57	56.4				67	0.31	0.19	0.53	<.001
Other	53	65.4				60	0.43	0.24	0.76	.004
						Hosmer & Lemeshow χ^2= 5.496, df=8, p=0.704. Final model χ^2=268.594, df=24, p<0.001 Nagelkerke = .087 Cases correctly classified: 82.1%. 86 cases excluded due to missing data on one or more independent variables.				

* adjusted for all other variables in the model, AOR, adjusted odds ratio; ref, reference category; 95% CI, 95% confidence interval.

For peer review only

For peer review only - http://bmjopen.bmj.com/site/about/guidelines.xhtml

Table S9 The World Health Organisation (WHO) – Association between trust in sources of information about COVID-19 vaccine and socio-demographic variables – (a) bivariate results and (b) multivariate logistic regression.

	**(a) Bivariate associations between socio-demographics and trusting COVID-19 vaccine info from The World Health Organisation (WHO)**<br>% Trust completely or a great deal (weighted)<br>χ^2 test for differences by demographics					**(b) Logistic regression of trust in COVID-19 vaccine info from The World Health Organisation (WHO)**<br>1 = Trust completely or a great deal (3423), 0 = Trust somewhat, very little or not at all (1468)				
	**n**	**%**		**χ^2 (df)**	**P**	**N**	**AOR***	**95% CI Lower**	**95% CI Upper**	**P**
**Gender**										
Male	1523	63.7		23.303#	<.001	2097	ref			<.001
Female	1797	70.1				2785	1.49	1.31	1.69	<.001
Other	5	71.4				9	1.92	0.39	9.42	.420
**Age**										
18-29	556	68.1		5.005 (6)	.543	458	ref			.177
30-39 *v 18-29*	549	64.5				760	0.84	0.65	1.10	.203
40-49 *v 18-39*	538	67.1				835	0.98	0.81	1.20	.882
50-59 *v 18-49*	593	68.4				896	1.15	0.96	1.39	.126
60-69 *v 18-59*	484	68.1				1004	0.89	0.76	1.06	.189
70-79 *v 18-69*	450	68.6				762	0.87	0.73	1.04	.127
80+ *v 18-79*	140	64.8				176	0.86	0.62	1.20	.366
**Education/Highest qualification**										
No qualifications	351	56.9		75.592 (4)	<.001	410	ref			<.001
Degree or equivalent and above	1490	72.3				2452	1.73	1.38	2.18	<.001
A levels / Vocational level 3 or equivalent	758	67.0				990	1.39	1.08	1.77	.010
Other qual'ns below A level / Voc level 3	561	66.9				784	1.36	1.06	1.76	.017
Other qualification	166	54.6				255	0.88	0.64	1.21	.428
**Financial Status**										
Living comfortably	953	74.2		67.486 (4)	<.001	1533	ref			<.001
Doing alright	1384	68.2				1995	0.78	0.66	0.91	.002
Just about getting by	691	61.0				960	0.60	0.50	0.73	<.001
Finding it quite difficult	204	60.4				266	0.56	0.42	0.75	<.001
Finding it very difficult	92	54.8				137	0.45	0.31	0.65	<.001
**Country**										
England	2880	67.4		2.028 (2)	.363	-				
Scotland	283	64.0				-	-	-	-	-
Wales	160	67.5				-	-	-	-	-
**Urban/rural**										
Urban	2663	66.7		1.288 (1)	.256	3727				
Rural	660	68.6				1164	0.99	0.85	1.15	.897
**Ethnicity**										
White British	2737	68.5		30.713 (5)	<.001	4224	ref			<.001
Any other white background	223	66.4				318	0.75	0.58	0.96	.023
Mixed or multiple ethnic groups	59	61.5				62	0.63	0.37	1.07	.089
Asian or Asian British	171	57.2				160	0.64	0.46	0.90	.010
Black or Black British	55	53.9				67	0.44	0.27	0.72	.001
Other	45	56.3				60	0.61	0.36	1.04	.068
						Hosmer & Lemeshow χ^2= 14.933, df=8, p=0.060.<br>Final model χ^2=172.240, df=22, p<0.001<br>Nagelkerke = .049<br>Cases correctly classified: 70.0%.<br>87 cases excluded due to missing data on one or more independent variables.				

* adjusted for all other variables in the model, AOR, adjusted odds ratio; ref, reference category; 95% CI, 95% confidence interval. # Fisher-Freeman-Halton Exact Test. Country was excluded from the logistic regression to achieve model fit.

For peer review only

Table S10 The media (e.g. newspapers, magazines, television, radio) – Association between trust in sources of information about COVID-19 vaccine and socio-demographic variables – (a) bivariate results and (b) multivariate logistic regression.

	**(a) Bivariate associations between socio-demographics and trusting COVID-19 vaccine info from the media** % Trust completely or a great deal (weighted) χ^2 test for differences by demographics					**(b) Logistic regression of trust in COVID-19 vaccine info from the media** 1 = Trust completely or a great deal (361), 0 = Trust somewhat, very little or not at all (4530)				
	**n**	**%**		**χ^2 (df)**	**P**	**N**	**AOR***	**95% CI Lower**	**95% CI Upper**	**P**
**Gender**										
Male	176	7.3		2.193 (2)	.334	2097				.575
Female	213	8.3				2785	1.13	0.90	1.41	.293
Other	0	0.0				9	0.00	0.00		.999
**Age**										
18-29	57	6.9		39.450 (6)	<.001	459				.003
30-39 *v 18-29*	57	6.7				759	0.84	0.52	1.35	.462
40-49 *v 18-39*	52	6.5				834	1.04	0.72	1.49	.848
50-59 *v 18-49*	58	6.7				895	1.08	0.78	1.50	.629
60-69 *v 18-59*	49	6.9				1004	1.05	0.77	1.42	.761
70-79 *v 18-69*	85	12.9				763	1.75	1.32	2.33	<.001
80+ *v 18-79*	29	13.3				177	1.74	1.07	2.83	.024
**Education/Highest qualification**										
No qualifications	77	12.5		34.152 (4)	<.001	409				.005
Degree or equivalent and above	153	7.4				2454	0.63	0.44	0.90	.011
A levels / Vocational level 3 or equivalent	58	5.1				990	0.44	0.29	0.68	<.001
Other qual'ns below A level / Voc level 3	79	9.4				784	0.72	0.48	1.07	.102
Other qualification	21	7.0				254	0.56	0.31	1.00	.051
**Financial Status**										
Living comfortably	105	8.2		34.041 (4)	<.001	1533				.671
Doing alright	122	6.0				1996	0.90	0.69	1.16	.413
Just about getting by	99	8.8				959	1.01	0.73	1.39	.974
Finding it quite difficult	33	9.8				266	1.22	0.75	1.98	.430
Finding it very difficult	30	17.2				137	0.79	0.37	1.68	.535
**Country**										
England	336	7.8		.515 (2)	.773	4300				.457
Scotland	32	7.3				383	0.79	0.51	1.24	.313
Wales	21	8.8				208	1.19	0.71	2.00	.499
**Urban/rural**										
Urban	323	8.1		1.957 (1)	.162	3726				
Rural	65	6.7				1165	0.91	0.70	1.19	.486
**Ethnicity**										
White British	296	7.4		6.645 (5)	.248	4226				.073
Any other white background	30	9.0				318	1.55	1.03	2.32	.035
Mixed or multiple ethnic groups	6	6.2				62	1.31	0.52	3.33	.570
Asian or Asian British	30	9.9				160	1.73	1.02	2.94	.043
Black or Black British	12	11.9				66	1.84	0.82	4.12	.139
Other	8	10.3				59	1.62	0.68	3.85	.274
						Hosmer & Lemeshow χ^2= 2.359, df=8, p=0.968. Final model χ^2=54.051, df=24, p<0.001 Nagelkerke = .027 Cases correctly classified: 92.6%. 87 cases excluded due to missing data on one or more independent variables.				

* adjusted for all other variables in the model, AOR, adjusted odds ratio; ref, reference category; 95% CI, 95% confidence interval.

For peer review only - http://bmjopen.bmj.com/site/about/guidelines.xhtml

Table S11 Social media – Association between trust in sources of information about COVID-19 vaccine and socio-demographic variables – (a) bivariate results and (b) multivariate logistic regression.

	**(a) Bivariate associations between socio-demographics and trusting COVID-19 vaccine info from social media** % Trust completely or a great deal (weighted) χ^2 test for differences by demographics					**(b) Logistic regression of trust in COVID-19 vaccine info from social media** 1 = Trust completely or a great deal (95), 0 = Trust somewhat, very little or not at all (4792)				
	**n**	**%**		**χ^2 (df)**	**P**	**N**	**AOR***	**95% CI Lower**	**95% CI Upper**	**P**
**Gender**										
Male	63	2.6		0.284 (2)	.868	2094	ref			.845
Female	71	2.8				2784	1.13	0.74	1.73	.561
Other	0	0.0				9	0.00	0.00		.999
**Age**										
18-29	31	3.8		12.626 (6)	.049	459	ref			.634
30-39 *v 18-29*	25	2.9				759	0.55	0.24	1.22	.139
40-49 *v 18-39*	22	2.7				835	0.89	0.47	1.67	.713
50-59 *v 18-49*	13	1.5				896	0.85	0.47	1.55	.596
60-69 *v 18-59*	13	1.8				1003	0.71	0.39	1.29	.259
70-79 *v 18-69*	19	2.9				761	1.11	0.62	1.97	.727
80+ *v 18-79*	9	4.2				174	1.26	0.49	3.25	.631
**Education/Highest qualification**										
No qualifications	31	5.1		24.978 (4)	<.001	409	ref			<.001
Degree or equivalent and above	44	2.1				2452	0.24	0.13	0.44	<.001
A levels / Vocational level 3 or equivalent	18	1.6				989	0.32	0.16	0.64	.001
Other qual'ns below A level / Voc level 3	32	3.8				782	0.50	0.26	0.94	.031
Other qualification	8	2.6				255	0.46	0.18	1.18	.106
**Financial Status**										
Living comfortably	34	2.6		26.413 (4)	<.001	1531	ref			.522
Doing alright	38	1.9				1994	0.73	0.43	1.24	.241
Just about getting by	35	3.1				959	0.99	0.55	1.79	.976
Finding it quite difficult	12	3.6				266	1.35	0.60	3.04	.473
Finding it very difficult	14	8.1				137	0.81	0.23	2.84	.746
**Country**										
England	118	2.8		.404(2)	.817	4295	ref			.215
Scotland	10	2.3				384	0.40	0.12	1.27	.120
Wales	6	2.5				208	1.42	0.56	3.58	.463
**Urban/rural**										
Urban	122	3.1		9.660(1)	.002	3724				
Rural	12	1.2				1163	0.57	0.31	1.05	.071
**Ethnicity**										
White British	94	2.4		17.781[#]	.002	4220	ref			.326
Any other white background	7	2.1				318	1.00	0.39	2.53	.994
Mixed or multiple ethnic groups	4	4.1				62	2.52	0.75	8.45	.134
Asian or Asian British	14	4.6				160	1.85	0.77	4.45	.172
Black or Black British	9	8.8				69	2.57	0.76	8.64	.128
Other	1	1.2				60	0.83	0.11	6.20	.855
						Hosmer & Lemeshow χ^2= 5.858, df=8, p=0.663.<br>Final model χ^2=46.839, df=24, p=0.004<br>Nagelkerke = .055<br>Cases correctly classified: 98.1%.<br>91 cases excluded due to missing data on one or more independent variables.				

* adjusted for all other variables in the model, AOR, adjusted odds ratio; ref, reference category; 95% CI, 95% confidence interval. [#] Fisher-Freeman-Halton Exact Test.

For peer review only - http://bmjopen.bmj.com/site/about/guidelines.xhtml

Table S12 Celebrities and social media influencers – Association between trust in sources of information about COVID-19 vaccine and socio-demographic variables – (a) bivariate results and (b) multivariate logistic regression.

	**(a) Bivariate associations between socio-demographics and trusting COVID-19 vaccine info from celebrities and social media influencers** % Trust completely or a great deal (weighted) χ^2 test for differences by demographics					**(b) Logistic regression of trust in COVID-19 vaccine info from celebrities and social media influencers** 1 = Trust completely or a great deal (95), 0 = Trust somewhat, very little or not at all (4795)				
	**n**	**%**		**χ^2 (df)**	**P**	**N**	**AOR***	**95% CI Lower**	**95% CI Upper**	**P**
**Gender**										
Male	57	2.4		1.430 (2)	.489	2097	ref			.996
Female	74	2.9				2784	0.98	0.65	1.49	.928
Other	0	0.0				9	0.00	0.00		.999
**Age**										
18-29	24	2.9		19.156 (6)	.004	459	ref			.348
30-39 *v 18-29*	25	3.0				758	0.90	0.37	2.21	.818
40-49 *v 18-39*	23	2.9				835	1.11	0.58	2.14	.744
50-59 *v 18-49*	18	2.1				895	1.28	0.73	2.25	.390
60-69 *v 18-59*	9	1.3				1004	0.65	0.34	1.25	.192
70-79 *v 18-69*	17	2.6				762	1.26	0.70	2.28	.441
80+ *v 18-79*	14	6.4				177	2.18	0.96	4.98	.064
**Education/Highest qualification**										
No qualifications	35	5.7		58.886 (4)	<.001	409	ref			.002
Degree or equivalent and above	32	1.5				2453	0.31	0.17	0.58	<.001
A levels / Vocational level 3 or equivalent	13	1.1				990	0.31	0.15	0.65	.002
Other qual'ns below A level / Voc level 3	41	4.9				783	0.59	0.31	1.11	.103
Other qualification	10	3.3				255	0.57	0.24	1.40	.221
**Financial Status**										
Living comfortably	23	1.8		53.820 (4)	<.001	1533	ref			.022
Doing alright	27	1.3				1995	1.01	0.56	1.82	.975
Just about getting by	59	5.2				959	2.08	1.13	3.80	.018
Finding it quite difficult	11	3.3				266	2.47	1.08	5.64	.032
Finding it very difficult	10	5.7				137	1.86	0.60	5.77	.284
**Country**										
England	117	2.7		11.948 (2)	.003	4299	ref			.028
Scotland	3	0.7				383	0.40	0.13	1.29	.127
Wales	12	5.0				208	2.26	1.06	4.82	.036
**Urban/rural**										
Urban	119	3.0		9.096 (1)	.003	3725				
Rural	12	1.2				1165	0.64	0.36	1.14	.133
**Ethnicity**										
White British	94	2.4		14.208[#]	.008	4224	ref			.574
Any other white background	11	3.3				318	1.62	0.75	3.47	.217
Mixed or multiple ethnic groups	1	1.0				62	0.84	0.11	6.27	.866
Asian or Asian British	10	3.3				160	1.58	0.61	4.09	.346
Black or Black British	9	8.9				66	2.36	0.70	7.94	.166
Other	1	1.2				60	0.94	0.13	7.04	.956
						Hosmer & Lemeshow χ^2= 9.111, df=8, p=0.333. Final model χ^2=57.132, df=24, p<0.001 Nagelkerke = .067 Cases correctly classified: 98.1%. 88 cases excluded due to missing data on one or more independent variables.				

* adjusted for all other variables in the model, AOR, adjusted odds ratio; ref, reference category; 95% CI, 95% confidence interval.

For peer review only - http://bmjopen.bmj.com/site/about/guidelines.xhtml

Table S13 Family and friends – Association between trust in sources of information about COVID-19 vaccine and socio-demographic variables – (a) bivariate results and (b) multivariate logistic regression.

	**(a) Bivariate associations between socio-demographics and trusting COVID-19 vaccine info from family and friends** % Trust completely or a great deal (weighted) χ^2 test for differences by demographics					**(b) Logistic regression of trust in COVID-19 vaccine info from family and friends** 1 = Trust completely or a great deal (1139), 0 = Trust somewhat, very little or not at all (3752)				
	**n**	**%**		**χ^2 (df)**	**P**	**N**	**AOR***	**95% CI Lower**	**95% CI Upper**	**P**
**Gender**										
Male	557	23.2		4.455 (2)	.108	2098	ref			.053
Female	660	25.8				2784	1.17	1.01	1.34	.030
Other	2	28.6				9	2.39	0.58	9.85	.227
**Age**										
18-29	140	17.0		109.226 (6)	<.001	459	ref			<.001
30-39 *v 18-29*	194	22.8				759	1.28	0.94	1.73	.112
40-49 *v 18-39*	186	23.3				834	1.18	0.94	1.47	.157
50-59 *v 18-49*	182	21.0				896	0.93	0.76	1.15	.504
60-69 *v 18-59*	176	24.8				1004	1.11	0.92	1.33	.283
70-79 *v 18-69*	233	35.6				762	1.85	1.54	2.23	<.001
80+ *v 18-79*	90	41.1				177	2.33	1.69	3.20	<.001
**Education/Highest qualification**										<.001
No qualifications	203	33.0		70.692 (4)	<.001	410	ref			<.001
Degree or equivalent and above	403	19.4				2454	0.61	0.47	0.77	<.001
A levels / Vocational level 3 or equivalent	271	23.9				990	0.81	0.62	1.05	.112
Other qual'ns below A level / Voc level 3	254	30.4				783	0.99	0.76	1.29	.925
Other qualification	86	28.5				254	0.96	0.68	1.36	.812
**Financial Status**										
Living comfortably	330	25.7		6.383 (4)	.172	1533	ref			.667
Doing alright	470	23.1				1997	0.94	0.79	1.10	.424
Just about getting by	300	26.6				958	1.00	0.81	1.22	.976
Finding it quite difficult	81	24.0				266	1.03	0.75	1.42	.856
Finding it very difficult	38	21.8				137	0.75	0.47	1.18	.212
**Country**										
England	1051	24.6		13.592 (2)	.001	4299	ref			.012
Scotland	89	20.1				384	1.00	0.77	1.29	.976
Wales	78	32.9				208	1.59	1.17	2.17	.003
**Urban/rural**										
Urban	979	24.5		.029 (1)	.866	3726				
Rural	239	24.8				1165	0.91	0.77	1.07	.237
**Ethnicity**										
White British	1005	25.1		36.523 (5)	<.001	4226	ref			.029
Any other white background	50	15.0				317	0.79	0.58	1.07	.128
Mixed or multiple ethnic groups	15	15.5				62	0.76	0.38	1.51	.428
Asian or Asian British	100	32.8				160	1.52	1.06	2.18	.024
Black or Black British	27	26.7				67	1.39	0.79	2.46	.250
Other	12	15.2				59	0.53	0.24	1.18	.122
						Hosmer & Lemeshow χ^2= 6.067, df=8, p=0.640. Final model χ^2=153.732, df=24, p<0.001 Nagelkerke = .047 Cases correctly classified: 76.7%. 87 cases excluded due to missing data on one or more independent variables.				

* adjusted for all other variables in the model, AOR, adjusted odds ratio; ref, reference category; 95% CI, 95% confidence interval.

For peer review only

Table S14 Faith or community leaders – Association between trust in sources of information about COVID-19 vaccine and socio-demographic variables – (a) bivariate results and (b) multivariate logistic regression.

	**(a) Bivariate associations between socio-demographics and trusting COVID-19 vaccine info from faith and community leaders** % Trust completely or a great deal (weighted) χ^2 test for differences by demographics					**(b) Logistic regression of trust in COVID-19 vaccine info from faith and community leaders** 1 = Trust completely or a great deal (161), 0 = Trust somewhat, very little or not at all (4724)				
	**n**	**%**		**χ^2 (df)**	**P**	**N**	**AOR***	**95% CI Lower**	**95% CI Upper**	**P**
**Gender**										
Male	118	4.9		17.452 (2)	<.001	2095	ref			.006
Female	135	5.3				2781	1.19	0.86	1.66	.294
Other	3	37.5				9	14.06	2.67	73.92	.002
**Age**										
18-29	47	5.7		20.879 (6)	.002	459	ref			.041
30-39 *v 18-29*	46	5.4				758	0.95	0.47	1.93	.880
40-49 *v 18-39*	31	3.9				834	1.11	0.65	1.89	.710
50-59 *v 18-49*	46	5.3				895	1.70	1.09	2.65	.020
60-69 *v 18-59*	20	2.8				1003	0.98	0.61	1.58	.933
70-79 *v 18-69*	42	6.4				760	1.62	1.03	2.55	.038
80+ *v 18-79*	20	9.2				176	2.28	1.15	4.56	.019
**Education/Highest qualification**										
No qualifications	57	9.3		37.137 (4)	<.001	407	ref			.011
Degree or equivalent and above	76	3.7				2451	0.42	0.25	0.71	<.001
A levels / Vocational level 3 or equivalent	51	4.5				990	0.57	0.33	0.99	.048
Other qual'ns below A level / Voc level 3	58	6.9				782	0.75	0.44	1.28	.290
Other qualification	15	4.9				255	0.50	0.22	1.14	.098
**Financial Status**										
Living comfortably	59	4.6		39.487 (4)	<.001	1530	ref			.042
Doing alright	71	3.5				1997	0.91	0.59	1.39	.663
Just about getting by	84	7.5				955	1.46	0.92	2.33	.110
Finding it quite difficult	20	5.9				266	2.05	1.09	3.84	.025
Finding it very difficult	20	11.5				137	1.44	0.60	3.44	.417
**Country**										
England	239	5.6		12.569 (2)	.002	4294	ref			.592
Scotland	11	2.5				383	0.69	0.33	1.43	.316
Wales	5	2.1				208	1.07	0.46	2.48	.877
**Urban/rural**										
Urban	229	5.7		13.640 (1)	<.001	3722				
Rural	27	2.8				1163	0.66	0.42	1.03	.068
**Ethnicity**										
White British	163	4.1		152.072(5)	<.001	4219	ref			<.001
Any other white background	12	3.6				318	1.22	0.60	2.46	.583
Mixed or multiple ethnic groups	3	3.1				62	2.59	0.90	7.42	.077
Asian or Asian British	59	19.6				159	4.82	2.76	8.42	<.001
Black or Black British	12	11.9				67	4.52	2.04	9.99	<.001
Other	2	2.5				60	1.37	0.32	5.77	.669
						Hosmer & Lemeshow χ^2= 11.202, df=8, p=0.191. Final model χ^2=87.282, df=24, p<0.001 Nagelkerke = .070 Cases correctly classified: 96.7%. 93 cases excluded due to missing data on one or more independent variables.				

* adjusted for all other variables in the model, AOR, adjusted odds ratio; ref, reference category; 95% CI, 95% confidence interval.

For peer review only - http://bmjopen.bmj.com/site/about/guidelines.xhtml

Supplementary material to 'A national survey of attitudes towards and intentions to vaccinate against COVID-19: implications for communications'.

Checklist: STROBE Statement – items that should be included in reports of cross-sectional studies

	Item No	Recommendation	Location where item is reported
**Title and abstract**	1	(*a*) Indicate the study's design with a commonly used term in the title or the abstract	pp. 1-2 *Title and abstract*
		(*b*) Provide in the abstract an informative and balanced summary of what was done and what was found	p.2 *Abstract*
**Introduction**			
Background/rationale	2	Explain the scientific background and rationale for the investigation being reported	p.4 *Introduction*
Objectives	3	State specific objectives, including any prespecified hypotheses	p.4 *Introduction (third paragraph). As this was the first survey of this type which addressed actual, rather than hypothetical, vaccines; we conducted an exploratory investigation rather than testing hypotheses.*
**Methods**			
Study design	4	Present key elements of study design early in the paper	p.5 *Methods (opening paragraph)*
Setting	5	Describe the setting, locations, and relevant dates, including periods of recruitment, exposure, follow-up, and data collection	p.6 *Methods: Sample and data collection*
Participants	6	(*a*) Give the eligibility criteria, and the sources and methods of selection of participants	p.6 *Methods: Sample and data collection;*
Variables	7	Clearly define all outcomes, exposures, predictors, potential confounders, and effect modifiers. Give diagnostic criteria, if applicable	pp.5-6; and pp.S2-S13 (in Supplementary Material) *Methods: Measures; and Methods S1*
Data sources/ measurement	8*	For each variable of interest, give sources of data and details of methods of assessment (measurement). Describe comparability of assessment methods if there is more than one group	pp.5-6; and pp.S2-S13 (in Supplementary Material) *Methods: Measures; and Methods S1*
Bias	9	Describe any efforts to address potential sources of bias	p.6 *Methods: Sample and data collection (Survey used a probability sample; participants accessed online or by telephone, sent reminders–addressing response bias)*
Study size	10	Explain how the study size was arrived at	p.6 *Methods: Sample and data collection*
Quantitative variables	11	Explain how quantitative variables were handled in the analyses. If applicable, describe which groupings were chosen and why	pp.6-7 *Methods: Data analysis*
Statistical methods	12	(*a*) Describe all statistical methods, including those used to control for confounding	pp.6-7 *Methods: Data analysis*
		(*b*) Describe any methods used to examine subgroups and interactions	pp.6-7 *Methods: Data analysis*
		(*c*) Explain how missing data were addressed	pp.6-7 *Methods: Data analysis*
		(*d*) If applicable, describe analytical methods taking account of sampling strategy	pp.6-7 *Methods: Data analysis*
		(*e*) Describe any sensitivity analyses	Not applicable
**Results**			
Participants	13*	(a) Report numbers of individuals at each stage of study—eg numbers potentially eligible, examined for eligibility, confirmed eligible, included in the study, completing follow-up, and analysed	p.S14 (in Supplementary Material) *Table S1*
		(b) Give reasons for non-participation at each stage	p.S14 (in Supplementary Material) *Table S1*
		(c) Consider use of a flow diagram	Not required

For peer review only - http://bmjopen.bmj.com/site/about/guidelines.xhtml

	Item No	Recommendation	Location where item is reported
Descriptive data	14*	(a) Give characteristics of study participants (eg demographic, clinical, social) and information on exposures and potential confounders	p.7; and pp.17-18 *Results: Sample characteristics; and Table 1*
		(b) Indicate number of participants with missing data for each variable of interest	pp.19-22 and pp.S16-S28 (in Supplementary Material) *Tables 2-4 and S2-S14*
Outcome data	15*	Report numbers of outcome events or summary measures	pp.19-22 and pp.S16-S28 (in Supplementary Material) *Tables 2-4 and S2-S14*
Main results	16	(*a*) Give unadjusted estimates and, if applicable, confounder-adjusted estimates and their precision (eg, 95% confidence interval). Make clear which confounders were adjusted for and why they were included	pp.7-9; pp.21-22 and pp.S16-S28 (in Supplementary Material) *Results; Tables 3-4 and S2-S14*
		(*b*) Report category boundaries when continuous variables were categorized	Not applicable (all variables were categorical)
		(*c*) If relevant, consider translating estimates of relative risk into absolute risk for a meaningful time period	Not applicable
Other analyses	17	Report other analyses done—eg analyses of subgroups and interactions, and sensitivity analyses	pp.S16-S28 (in Supplementary Material) *Tables S2-S14 (subgroup analyses of the 13 COVID-19 vaccine information sources)*
**Discussion**			
Key results	18	Summarise key results with reference to study objectives	p.9 *Discussion: Principal findings*
Limitations	19	Discuss limitations of the study, taking into account sources of potential bias or imprecision. Discuss both direction and magnitude of any potential bias	p.11 *Discussion: Strengths and limitations*
Interpretation	20	Give a cautious overall interpretation of results considering objectives, limitations, multiplicity of analyses, results from similar studies, and other relevant evidence	pp.9-11 *Discussion: Comparison with other studies; and Discussion: Strengths and limitations*
Generalisability	21	Discuss the generalisability (external validity) of the study results	p.11 *Discussion: Strengths and limitations*
**Other information**			
Funding	22	Give the source of funding and the role of the funders for the present study and, if applicable, for the original study on which the present article is based	p.12 *Role of the funding source*

*Give information separately for exposed and unexposed groups.

For peer review only - http://bmjopen.bmj.com/site/about/guidelines.xhtml
